# Developmental landscape of computational techniques to explore the potential phytochemicals from *Punica granatum* peels for their antioxidant activity in Alzheimer’s disease

**DOI:** 10.3389/fmolb.2023.1252178

**Published:** 2023-10-11

**Authors:** Shagufta Parveen, Aneeqa Batool, Nusrat Shafiq, Maryam Rashid, Ayesha Sultan, Gezahign Fentahun Wondmie, Yousef A. Bin Jardan, Simone Brogi, Mohammed Bourhia

**Affiliations:** ^1^ Synthetic and Natural Product Drug Discovery Laboratory, Department of Chemistry, Government College Women University, Faisalababd, Pakistan; ^2^ Department of Chemistry, University of Education, Lahore, Pakistan; ^3^ Department of Biology, Bahir Dar University, Bahir Dar, Ethiopia; ^4^ Department of Pharmaceutics, College of Pharmacy, King Saud University, Riyadh, Saudi Arabia; ^5^ Department of Pharmacy, Pisa University, Pisa, Italy; ^6^ Department of Chemistry and Biochemistry, Faculty of Medicine and Pharmacy, Ibn Zohr University, Laayoune, Morocco

**Keywords:** antioxidant, acetylcholine, amyloid beta, Alzheimer’s disease, pharmacophore

## Abstract

Alzheimer’s disease (AD) is more commonly found in women than in men as the risk increases with age. Phytochemicals are screened *in silico* from *Punica granatum* peels for their antioxidant activity to be utilized for Alzheimer’s disease. Alzheimer’s disease is inhibited by the hormone estrogen, which protects the brain from the bad effects of amyloid beta and acetylcholine (ACh), and is important for memory processing. For the purpose, a library of about 1,000 compounds from *P. granatum* were prepared and studied by applying integrated computational calculations like 3D-QSAR, molecular docking, MD simulation, ADMET, and density functional theory (DFT). The 3D-QSAR model screened the active compounds B25, B29, B35, B40, B45, B46, B48, B61, and B66 by the field points and activity atlas model from the prepared library. At the molecular level, docking was performed on active compounds for leading hit compounds such as B25 and B35 that displayed a high MolDock score, efficacy, and compatibility with drug delivery against the antioxidant activity. Optimization of the structure and chemical reactivity parameter of the hit compound was calculated by DFT. Moreover, ADMET prediction was evaluated to check the bioavailability and toxicity of the hit compound. Hesperidin **(B25)** is found to be a hit compound after the whole study and can be synthesized for potent drug discovery in the future.

## Graphical Abstract

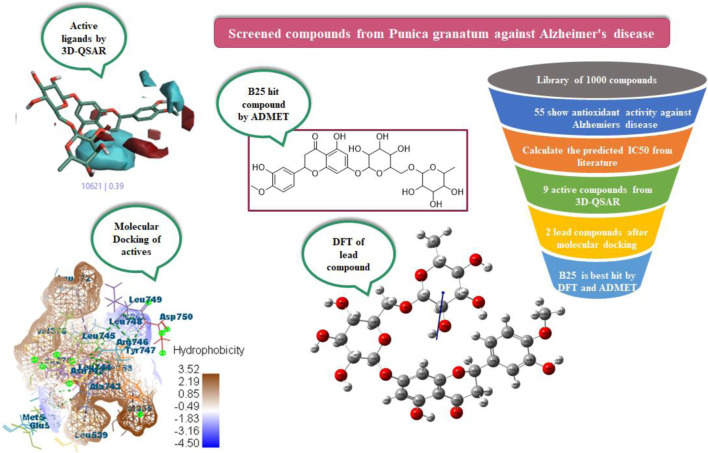

## 1 Introduction

Antioxidants are compounds that prevent the oxidation step from starting and its growth. Numerous disorders, including those affecting the brain, diabetes, Parkinson’s disease, heart, and arthritis, are caused due to deficiency of antioxidants (Young and Woodside, 2001). By reducing oxidative stress, regular use of natural antioxidants offers protection against cancer, age-related diseases, and cardiovascular disorders (Søholm, 1998). Additionally, an excess of free radicals due to oxidative stress is linked to the pathogenesis of Alzheimer’s disease (AD). Several oxidative stress-related disorders, including cancer and neurological diseases, can be avoided by consuming *Punica granatum* because of their phytochemicals exhibiting antioxidant properties (Benchagra et al., 2021). Alzheimer’s disease (AD) is a serious neurological condition that primarily affects elder people. Although the exact causes of AD are unknown, three current mechanisms, namely, the activation of oxidative stress, the termination of cholinergic synapses, and the creation of beta-amyloid, are assumed to cause AD (Kihara and Shimohama, 2004).

The class of drug best examined for potential help in preventing Alzheimer’s disease (AD) is estrogens. These steroids produce actions that are compatible with their potential utility in the inhibition of Alzheimer’s disease and are powerful neuroprotectants both *in vivo* and *in vitro* (Kendall et al., 2005). This includes the avoidance of the conversion of amyloid precursor protein (APP) into beta-amyloid (Russo et al., 2005). Deficiency of acetylcholine (ACh) and the accumulation of β-amyloid plaques in the human brain are the two main contributors to Alzheimer’s disease (Georgiev et al., 2012). Neuronal structural flaws are caused by the slow accumulation of β-amyloid plaques and ongoing oxidative stress (Tuppo and Arias, 2005). Functional, mental, and behavioral abnormalities and death might result from this process. The risk of Alzheimer’s disease is increased by pathophysiological circumstances that speed up the formation of amyloid plaques in the brain (Melo et al., 2015).

In order to cure diseases linked to free radicals, the discovery of antioxidants from natural sources is increasing (Zhang and Tsao, 2016). Reactive oxygen species (ROS) are produced by an organism’s regular biological and physiological activities (Min and Min, 2014). Several oxidative stress-related disorders, including AD, may be caused by the disproportion of radical overproduction (Choi et al., 2014). Reduced synthesis of acetylcholine (ACh), a neurotransmitter in the brain involved in memory, thought, and decision-making, further supports the idea that the loss of cholinergic synapses causes Alzheimer’s disease (AD) (Rao et al., 2007). The two main enzymes involved in the progression of AD are acetylcholinesterase (AChE) and butyrylcholinesterase (BChE). The effectiveness of cholinesterase inhibitors against minor, moderate, and severe forms of AD has been consistent (Anand and Singh, 2013). Treatments using inhibitors of the acetylcholinesterase enzyme, however, are subject to several negative effects. Galantamine is an anti-cholinesterase medication that is derived from plant extracts (Rao et al., 2007). Therefore, expanding the selection of naturally occurring compounds containing acetylcholinesterase inhibitor potential might be beneficial. One of the key pathological characteristics of AD is the β-amyloid protein, which is a pro-inflammatory substance.

*Punica* is a tiny genus of deciduous shrubs that yield fruits. Pomegranate (*P. granatum*) is a more widely known species that belongs to the Punicaceae family (Levin, 1994). Phenolic antioxidants are abundant in pomegranate (*P. granatum*) and its manufacturing byproducts. In current studies, the scientific information demonstrating this fruit’s multiple health benefits increases its interest (Al-Jarallah et al., 2013). In reality, pomegranate bio-actives may lower the chance of developing cancer and Alzheimer’s disease (Ambigaipalan et al., 2016).

Pomegranate (*P. granatum*) is a small, long-lived tree that is grown across the Mediterranean region, Southeast Asia, the Himalayas, California, and the United States. In historical applications, pomegranate is utilized in many medicines for several diseases (Levin, 1994). The combined effects of pomegranate’s components appear to be more effective than their individual effects. Frequent studies on the antioxidant activity of pomegranate components have been published in the last 10 years, with an emphasis on the treatment and avoidance of cancer, diabetes, antibiotic resistance, and skin damage brought on by UV radiation (Amorim et al., 2003). Pomegranate-mediated antioxidant activity has a vast range of additional medicinal benefits, including the treatment and prevention of disorders such as Alzheimer’s disease, arthritis, male infertility, obesity, diabetes, dental issues, and erectile dysfunction (Jurenka, 2008). Pomegranate fruits have been shown to contain bioactives and antioxidants that are essential for enhancing human health. The compounds from pomegranate fruit and juice are beneficial for human health but in higher quantities from its peels (Akhtar et al., 2015). In order to benefit humanity, scientists are interested in finding useful phytochemicals in fruit peels and using them in food processing sectors and pharmaceutical industries. Pomegranate peel contains the highest concentration of phytochemicals including flavonoids, phenolic acid, and tannins (Singh et al., 2017).

*P. granatum* has been the focus of numerous studies examining its characteristics and prospectives in medicinal applications, according to the search results (Rahimi et al., 2012) The *P. granatum* plant study is “excitingly novel” because it is thoroughly studied for its anti-inflammatory, antibacterial, antioxidant, and anticancer properties (Ranjha et al., 2021). Direct intake of pomegranate fruit is also very useful for health instead of using it through medicine.

**FIGURE 1:**
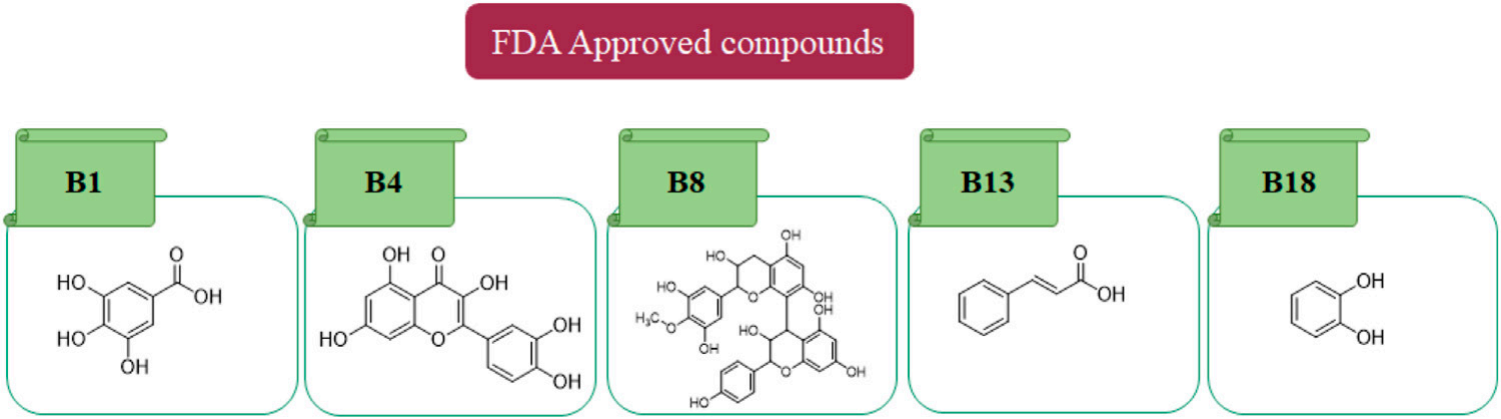
FDA-approved drugs used for drug designing.

*In in silico* studies, phytochemicals previously isolated from the peels of pomegranate were screened to inhibit Alzheimer’s disease. Screening of phytochemicals used for drug design, such as gallic acid **(B1)**, Quercetin **(B4)**, proanthocyanidin **(B8)**, cinnamic acid **(B13)**, and catechol **(B18)**, is FDA-approved (Figure 1). Drug repurposing is a technique used to improve an outdated drug to treat some diseases with pharmacological applications (Rudrapal et al., 2020). Hesperidin **(B61)** is an FDA-approved drug used for the prevention of allergies and high blood pressure but is now used to inhibit Alzheimer’s disease via the process of drug repurposing.

Nowadays, computational techniques are frequently used in biological research. *In silico* studies can assist in discovering pharmacological targets by the use of bioinformatics tools (Krovat et al., 2005). Additionally, they can be used to create candidate compounds, analyze the structure of the target for binding sites, assess how similar they are to drugs, target docks with these molecules, optimize the molecules, and rank them according to their binding affinities (Venkatesan et al., 2010). An *in silico* approach to drug design significantly reduces the expense and amount of time needed for a molecule throughout the drug discovery process. In *in silico* studies with virtual screening has a lot of potential for finding new medication candidates (Laskar et al., 2014). The effective discovery of drugs for treatment of many diseases is critical, but it is an expensive and long-term cycle. So, computational methods are used to find ligands from *P. granatum* peels, which are effective in drug discovery. To learn about the structural properties of active compounds, QSAR is used to check the structure–activity relationship, docking performance for lead compounds with scoring function, DFT for an optimized structure with chemical reaction description, and ADMET to check the bioavailability and toxicity of the hit compound.

## 2 Materials and methods

### 2.1 Approaches used for data collection and structural preparation for 3D-QSAR

Previous literature reports showed that a total of 1,000 phytochemicals were isolated from *P. granatum* peels. Later, from the literature and databases, including PubChem (https://pubchem.ncbi.nlm.nih.gov/), ZINC database (https://zinc12.docking.org/search/), DNP (https://dnp.chemnetbase.com/chemical/ChemicalSearch.xhtml?dswid=-3450), ChEMBL (https://www.ebi.ac.uk/chembl/), Natural II database (https://bioinf-applied.charite.de/supernatural_3/subpages/compounds.php), and published articles, almost 55 ligands were screened with good antioxidant activity (Supplementary Table S1). A 3D-QSAR model was used to learn the antioxidant activity against Alzheimer’s disease. Then, 55 ligands were divided into training and test sets, with ratios of 80% and 20%. The experimental IC_50_ value was changed into the predicted IC_50_ value by using the following formula: pIC_50_ = −log (IC_50_). ChemDraw and Chem3D Ultra were used to draw the 2D and 3D structures by energy minimization performed in two steps (Alam and Khan, 2017). All ligands were included on an Excel sheet along with their experimental and predicted IC_50_ values to generate a CSV file.

In the 3D-QSAR model, the “Flare module of Cresset” tool was used for minimization and pre-processing of all the compounds and pharmacophore generation (Verma et al., 2022a). This tool gives us the best, good-quality, and high-resolution pictures compared to other QSAR tools like Maestro Schrodinger and QSAR-clouds.

### 2.2 Approaches used for pharmacophore generation in the development of 3D-QSAR

In pharmacophore generation, the hypothesis of 3D conformation for active ligands is generated with its field points by using flares (Table 1). Four molecular fields were determined by field points such as geometry, hydrophobicity, and electrostatics (negative and positive) to develop a pharmacophore pattern. Screened phytochemicals from *P. granatum* peels are arranged as the test and training sets using a sphere exclusion algorithm on a flare for a 3D-QSAR study. These 55 screened compounds are divided into 34 compounds in the training set and 20 compounds in the test set, and one reference drug was also added. Edaravone is a reference drug that is also FDA-approved and selected from the literature for antioxidant activity. The PLS (partial least squares) model was also used to develop a good field QSAR model for compounds (Wold et al., 2001).

**TABLE 1:** Screened out active compounds by the 3D-QSAR model.

S. no.	Compound name	Compound structure	IC_50_	Predicted IC_50_	Reference
**1.**	**Edaravone**	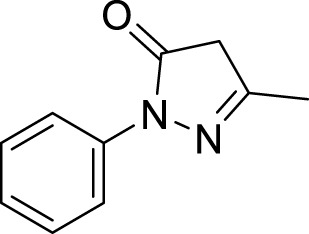	0.3	Reference (standard drug)	Rothstein (2017)
**2.**	**Hesperidin (B25)**	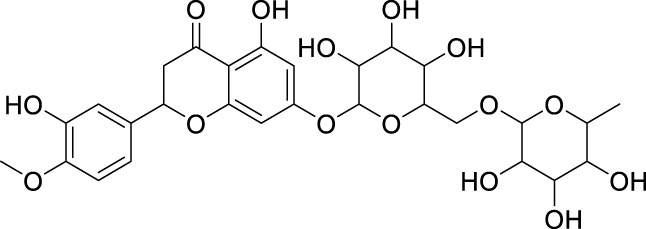	0.43	0.39	Shalaby et al. (2019)
**3.**	**Apigenin (B29)**	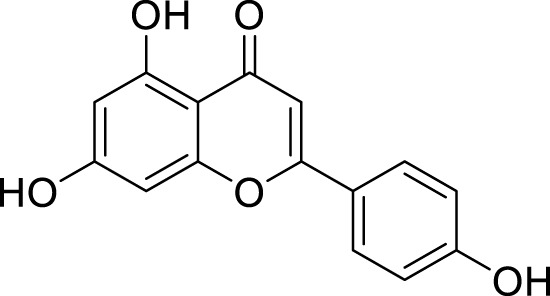	0.824	0.664	Singh et al. (2018)
**4.**	**Corilagin (B35)**	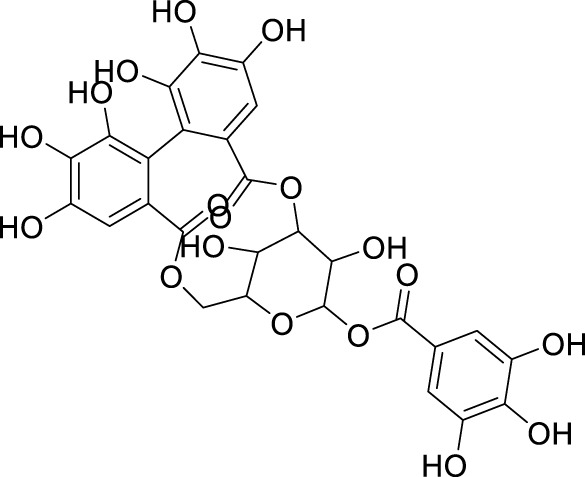	0.32	0.427	Das et al. (2021)
**5.**	**Ellagic acid (B40)**	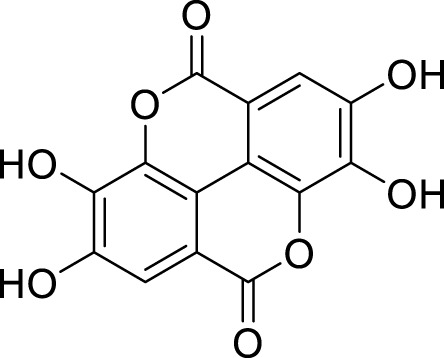	0.70	0.625	Das et al. (2021)
**6.**	**Kaempferol (B45)**	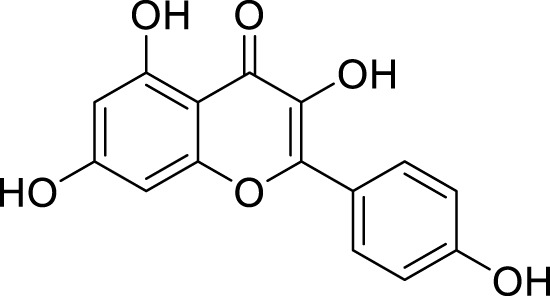	0.85	0.658	Das et al. (2021)
**7.**	**Myricetin (B46)**	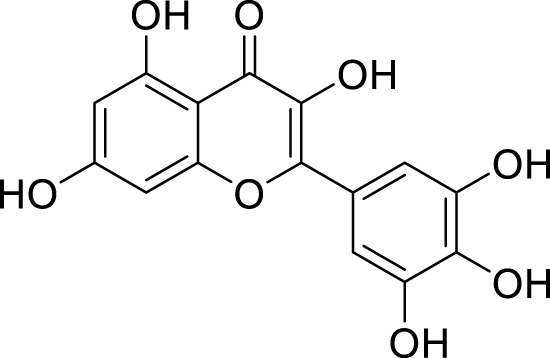	0.092	0.637	Sharma and Maity (2010)
**8.**	**Luteolin (B48)**	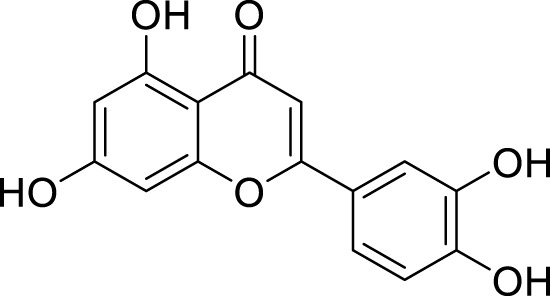	0.59	0.66	Das et al. (2021)
**9.**	**Taxifolin (B61)**	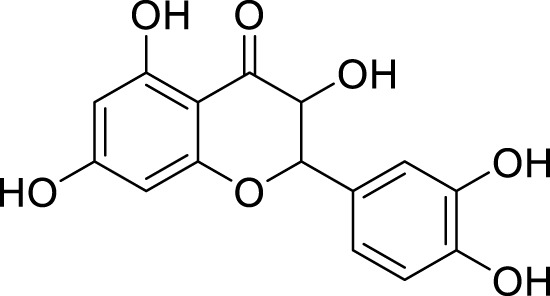	0.31	0.645	Farag et al. (2020)
**10.**	**Quercetin-3-glucoside (B66)**	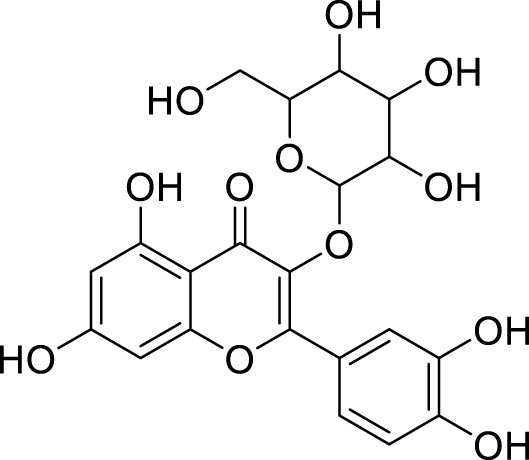	1.9	0.514	Shalaby et al. (2019)

### 2.3 Use of different regression coefficients for 3D-QSAR model validation

The 3D-QSAR model provides us a better understanding of the structure–activity relationship (SAR) of many ligands in terms of the characteristics of shape, positive and negative van der Waals forces, and hydrophobicity. The regression coefficient (r^2^), cross-validated coefficient of determination (q^2^), and the conformer scores for active compounds with respect to the axis were used to validate the best model. The leave-one-out (LOO) method was used to evaluate the developed QSAR model in order to improve the prediction of the activity model. One of the best techniques for the validation of regression models with limited training datasets is LOO cross-validation (Alam and Khan, 2014). The activity atlas model provides data about the cliff summary that shows the positive and negative electrostatics, an average of actives, and hydrophobicity.

### 2.4 Molecular docking technique

Molecular docking of screened compounds from *P. granatum* peels was performed by Molegro Virtual Docker (MVD). MVD is a protein–ligand docking simulation program that enables us to simulate the docking in the full computational environment (Kusumaningrum et al., 2014). MVD includes the entire docking process including the arrangement of protein’s binding sites, the prediction of ligand modes, and the creation of the pose by MolDock re-ranked (Bitencourt-Ferreira and Azevedo, 2019).

MVD software used protein and ligand docking, where structures of proteins were imported and water molecules were removed (Boittier et al., 2020). The MVD tool gives us more reliable results regarding protein–ligand complexes (Ozalp et al., 2018). This tool consumes less time and energy for docking compared to AutoDock 4, AutoDock Vina, and rDock.

#### 2.4.1 Protein–ligand preparation

In MVD, the SDF format of the ligand 3D structure downloaded from PubChem was loaded on the workspace with the targeted protein. Proteins linked to estrogen and acetylcholine hormones were downloaded from RCSB: PDB (https://www.rcsb.org/) (protein structure database) and must be *Homo sapiens* organisms with a resolution of less than 2.50Å for good results (Table 2).

**TABLE 2:** Preparation of proteins for molecular docking.

Sr. no.	PDB ID	Protein name	Ligand	Resolution (Å)	Molecule	Organism	Reference
**1.**	**7KOQ**	Acetylcholine receptor with epibatidine	Epibatidine	3.60	Acetylcholine	*Homo sapiens*	https://www.rcsb.org/structure/7KOQ
**2.**	**5ONP**	Alzheimer’s amyloid-beta complex with thermolysin	Cadmium ion	1.34	Beta-amyloid	*Homo sapiens*	https://www.rcsb.org/structure/5onp
**3.**	**1GWR**	Estrogen receptor ligand complex with estradiol	Estradiol	2.40	Estrogen	*Homo sapiens*	https://www.rcsb.org/structure/1gwr
**4.**	**4AA6**	Estrogen receptor through a side chain	Zinc ion	2.60	Estrogen	*Homo sapiens*	https://www.rcsb.org/structure/4aa6

### 2.5 Molecular dynamics (MD) simulation

MD simulation of protein–ligand complexes was performed on an iMOD server (https://imods.iqfr.csic.es/) that can be used for structural flexibility assessment for all the proteins. The root-mean-square fluctuation (RMSF) was attained on the basis of nuclear magnetic resonance (NMR) with the default option. The simulation time of 10 ns was adjusted, although the other parameters were also adjusted as default values (Yao et al., 2016). The structural dynamics and the molecular motion determination of the docking compounds were analyzed by the iMOD server. The stability of protein–ligand complexes was portrayed with reference to eigenvalue, deformability, covariance map, B-factor, and elastic network.

The 3D structures of corilagin and hesperidin compounds were downloaded from PubChem in the SDF format (Kim et al., 2019). After that, it was converted into the MOL2 formats by opening Babel software. The proteins 1GWR, 4AA6, 5ONP, and 7KOQ were downloaded from the RCSB: PDB server with better resolution scores (Burley et al., 2019). The residues and warnings were removed using Molegro Virtual Docker.

Molecular dynamics simulation was performed using the iMOD online server, which exploits the advantages of normal mode analysis (NMA) classification (Lopéz-Blanco et al., 2011). iMOD can also calculate the vibrational modes of protein’s multiple chains and support the ligand. iMOD is a faster free online tool that consumes less memory than other MD simulation tools like GROMACS and Maestro Schrodinger, which are highly expensive.

### 2.6 Computed approaches employed for the optimization of phytochemical geometries

The phytochemical structure optimized from *P. granatum* peels was evaluated using density functional theory for theoretical studies, and its chemical properties were calculated (Obot et al., 2015). The parameters of DFT show the dipole moment, electronegativity, chemical hardness and softness, chemical potential, and energy gap. DFT was performed on hit leading compounds to check their HOMO and LUMO values and the geometry of the optimized structure.

### 2.7 ADME-toxicity determination to check drug-likeness

In the process of drug discovery, physiochemical, lipophilicity, and pharmacokinetics properties increase productivity through predictive tools (Li, 2001). For the study of compounds, computational methods are developed for absorption, distribution, metabolism, excretion, and toxicity (ADME-Tox) properties. SwissADME is an online free server used to check the absorption and distribution of compounds (http://www.swissadme.ch/). ADMET was performed for the hit compound to check its efficiency and toxicity. ProTox-II is also a free online server used to check the toxicity of compounds (https://tox-new.charite.de/protox_II/).

## 3 Results and discussion

### 3.1 Conformation hunt and pharmacophore generation of active compounds by the 3D-QSAR study

**FIGURE 2:**
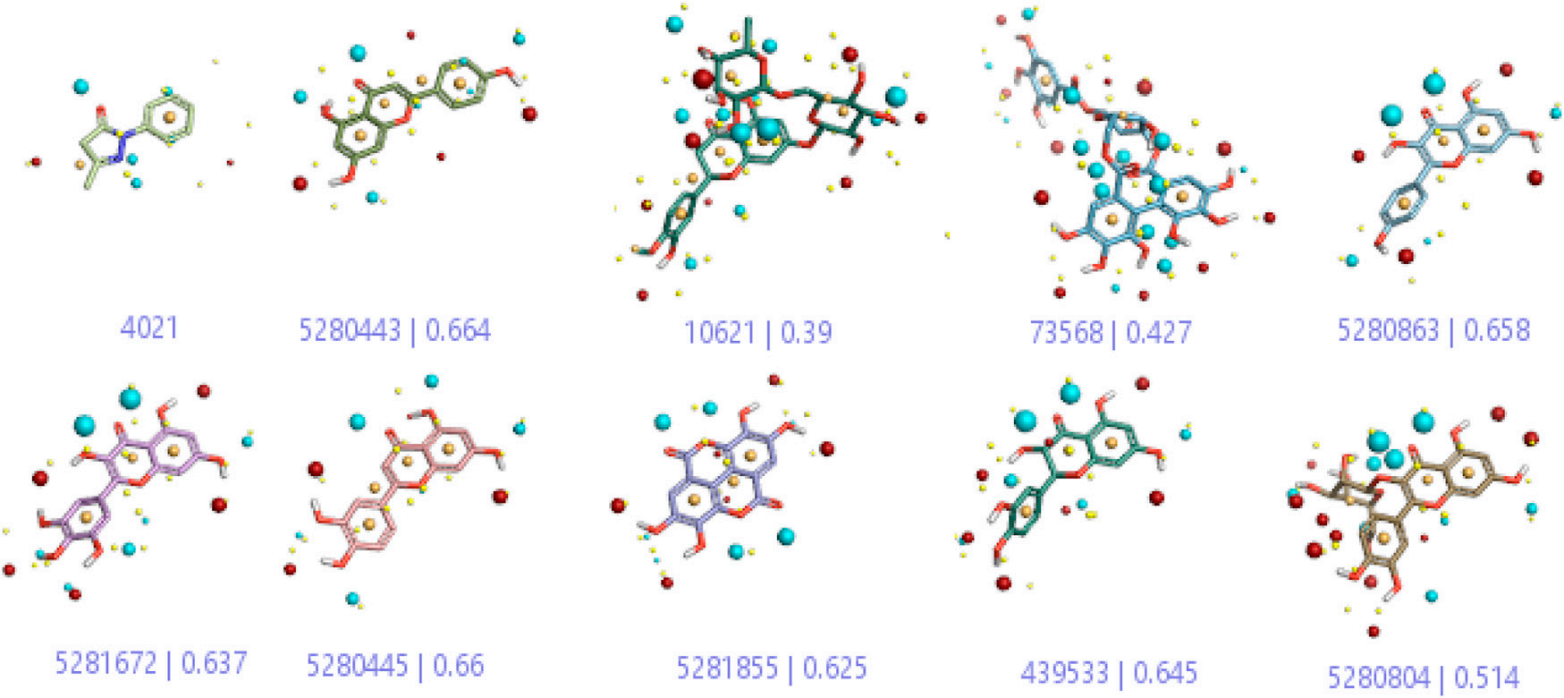
Pharmacophore generation shows hydrophobicity in orange color, negative electrostatics in red, positive electrostatics in blue color, and van der Waals descriptors in yellow color (4,021 is a reference drug).

In a previous study, there was no specific work on antioxidant activity for the inhibition of Alzheimer’s disease that showed the mechanism of action. QSAR is a field-based technique of molecules showing the SAR of compounds using flare software. Nine active compounds, namely, **B25**, **B29**, **B35**, **B40**, **B45**, **B46**, **B48**, **B61**, and **B66** were used as templates to conduct a conformation hunt (Figure 2). The calculated field points for the deduced perception of the active compounds were then interpreted, leading to the discovery of a 3D-field point’s pattern (Sadekar et al., 2021). The XED force field was used to create field points. These active compounds are used to create a template of pharmacophore for further screening.

#### 3.1.1 Alignment of compounds from 3D-QSAR in the training set

All the 55 ligands in the training set of selected field templates for Alzheimer’s disease were aligned for a model of 3D-QSAR. The experimental values were changed into the positive scale logarithm using the equation pIC_50_ = log (IC_50_). The 3D-QSAR model set the data by splitting them into two sets with 34 in the training set, 20 in the test set, and 1 as the reference drug. The activity graph analysis that displayed the predicted or experimental value comparison graph using cross-validation was used to illustrate the robustness of the proposed 3D-QSAR model.

#### 3.1.2 Field points of identification and control by Alzheimer’s disease

**FIGURE 3:**
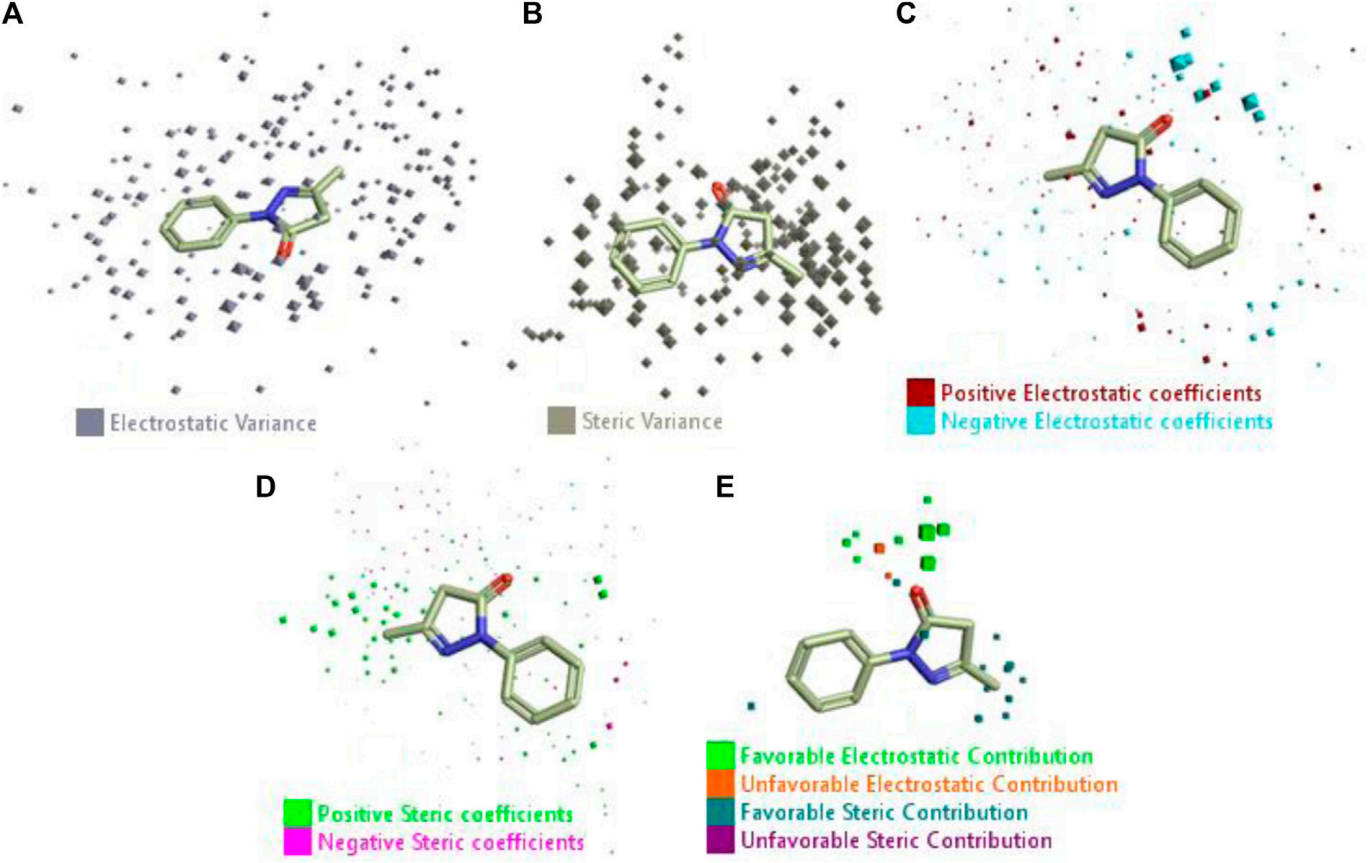
Molecular insight of structures shows electrostatic and steric coefficients, and variance from the QSAR model. **(A)** Electrostatic variance, **(B)** steric variance, **(C)** electrostatic coefficient, **(D)** steric coefficient, and **(E)** field points to predicted activity.

The 3D-QSAR model was generated to study the activity relationship of the structure for understanding the phytochemicals with antioxidant activity. Active ligands in the training set as the coefficient and the variance field points were explored in the three-dimensional structure form. Edaravone (reference drug) was also used to inhibit Alzheimer’s disease by hormones, and model points were compared for a better understanding of field points. The model showed the regions where it appears from the equation that local fields significantly affect the biological activity. There is a strong correlation between the electrostatic and steric fields in this position, and consequently, higher values of affinity enhance the biological activity. The results show that the green color specifies a positive steric coefficient leading to higher activity, while the red color shows a positive coefficient and the cyan color shows a negative coefficient of electrostatics. Electrostatic and steric field points with a large variance indicated significant changes, whereas those with a low variance show no changes (Figure 3).

3D-QSAR is a field-based study that shows the fields to predict activity performed on Alzheimer’s disease fit in a region of structural field points and also regulates the predicted activity. Geometry shows blue-, red-, and purple-colored field points, and the electrostatic and steric field points with positive and negative regulation toward the predicted activity (Figure 3E).

#### 3.1.3 Visualization activity atlas model through the SAR study

**FIGURE 4:**
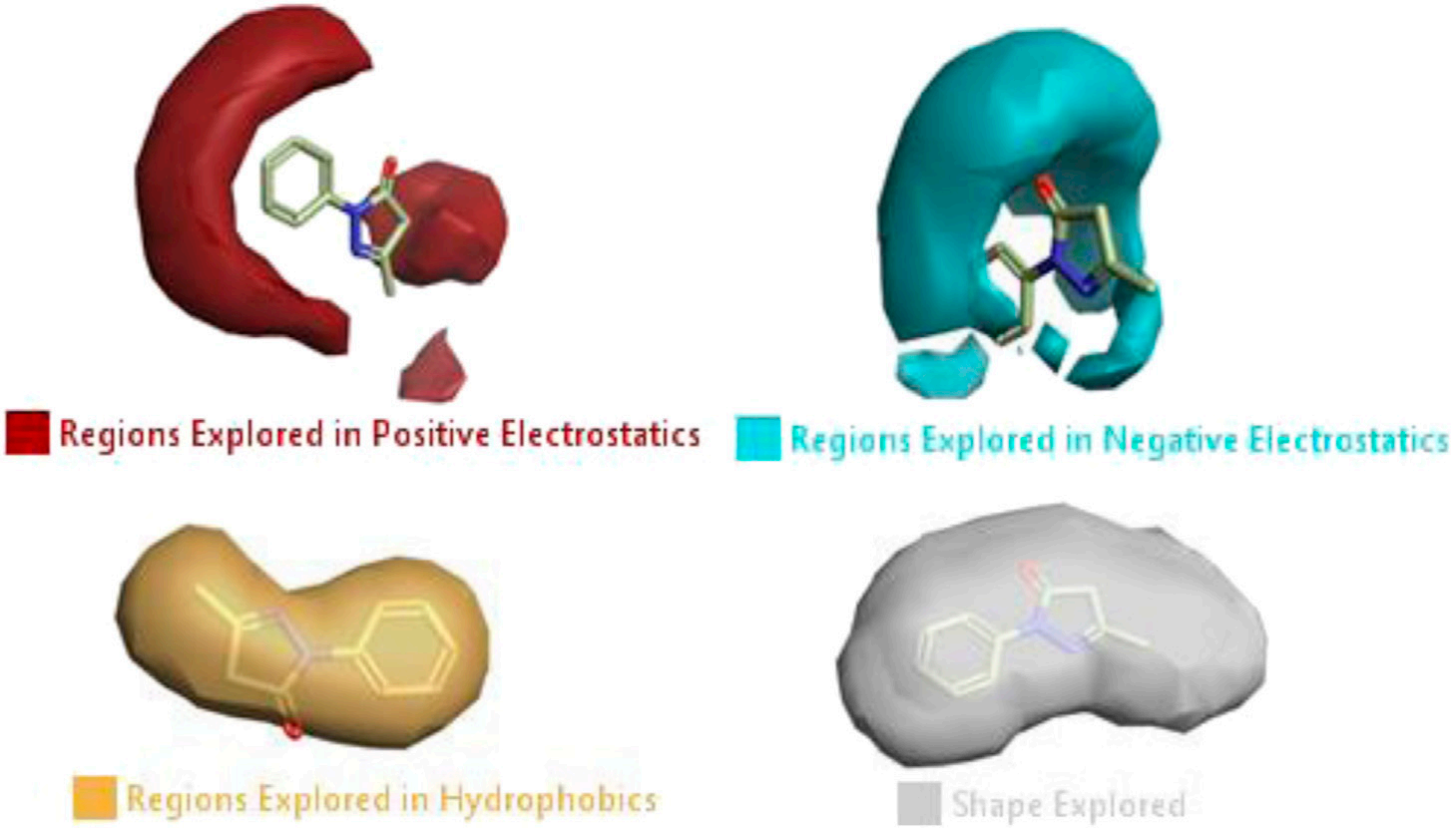
Optimized sites of active compounds by the SAR analysis model.

The activity atlas model of visualization explains the structure–activity relationships that show the features of antioxidant-active phytochemicals and lead to the optimization and designing of a drug for new analogs. A cliff summary of the average actives and activity of Alzheimer’s disease was studied. Inside the structure of active compounds, antioxidant activity shows a region of the positive field in red color as this region is higher than the activity, the average shape region is shown in white color, and hydrophobic interactions are shown in yellow color that control the antioxidant activity (Figure 4). Results also show the favorable, unfavorable, and weak regions the in SAR mechanism.

#### 3.1.4 Validation of the model by the use of activities in the training and test sets

In the SAR model, the molecular properties of active ligands are used to control Alzheimer’s disease through hormones with antioxidant activity, and compounds were screened for further prediction of antioxidant activity. According to the literature, in the QSAR model, binding targets are identified for ligand fields that are applied in virtual screening later (Table 3).

**TABLE 3:** Activity cliff summary of electrostatics, hydrophobics, and shapes along with average electrostatics, hydrophobics, and shapes of actives.

Compound name	Activity cliff summary of electrostatics	Activity cliff summary of hydrophobics	Activity cliff summary of shape	Average electrostatics of active	Average hydrophobics of active	Average shape of actives
**Edaravone (reference drug)**	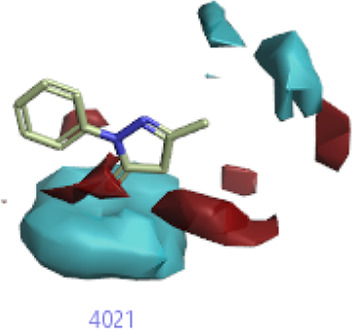	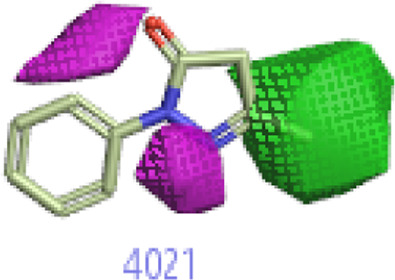	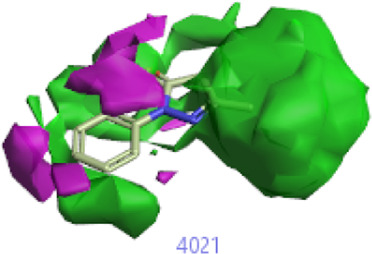	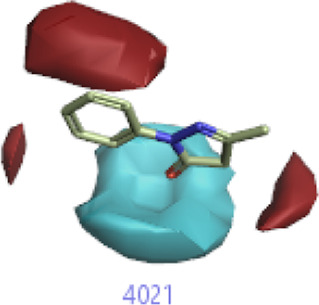	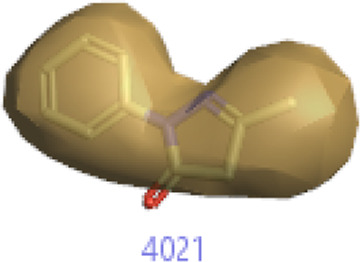	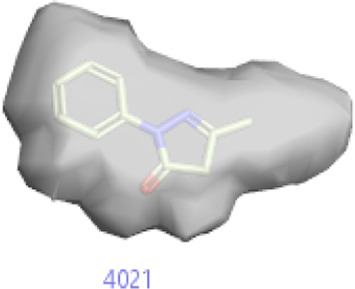
**Hesperidin (B25)**	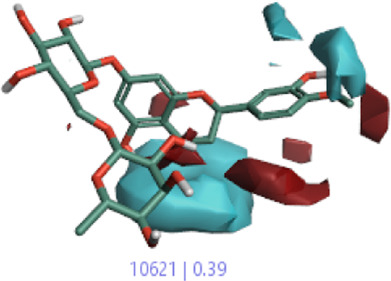	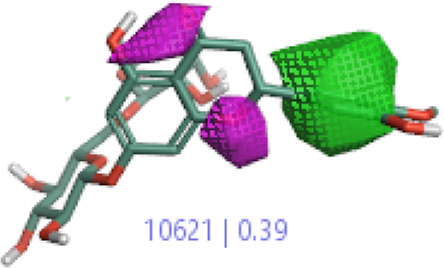	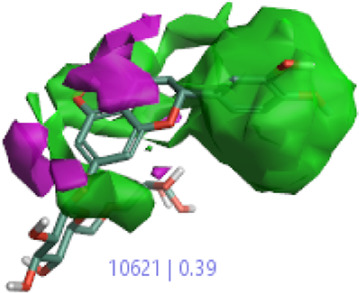	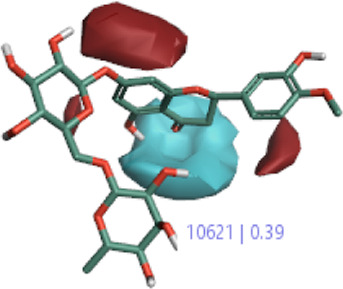	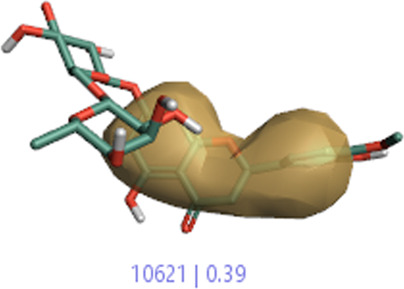	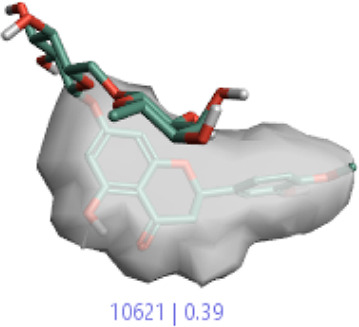
**Apigenin (B29)**	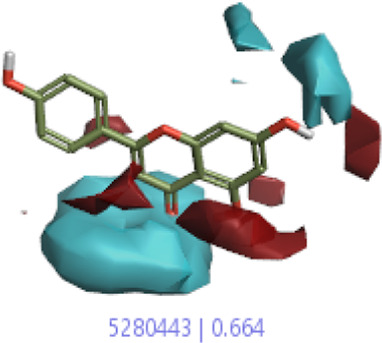	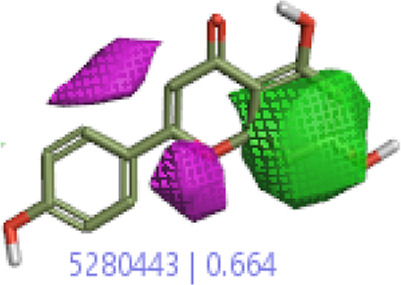	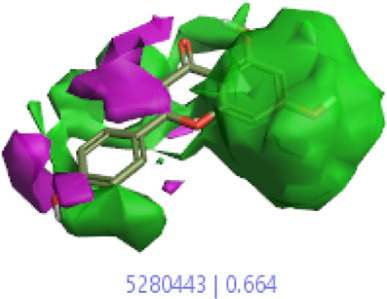	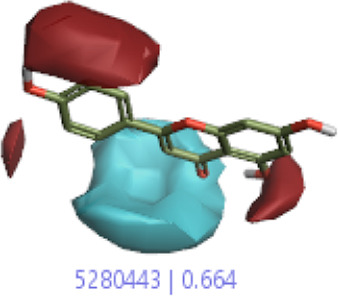	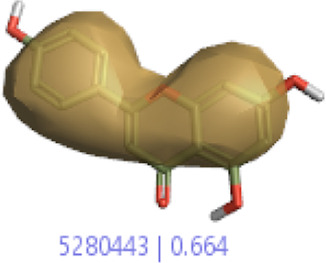	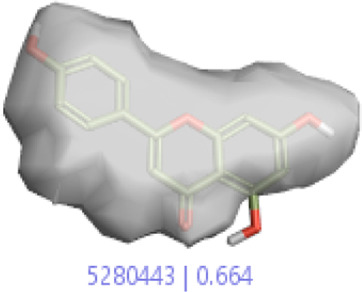
**Corilagin (B35)**	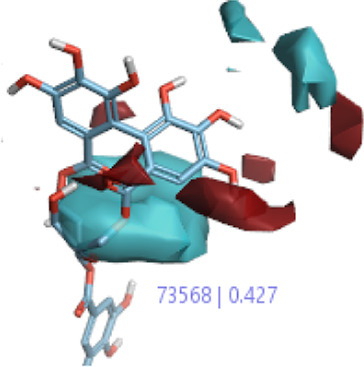	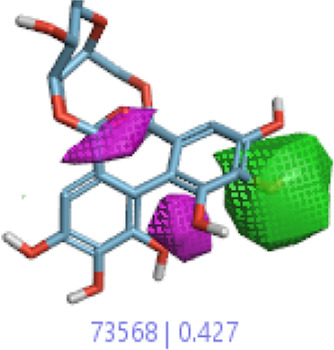	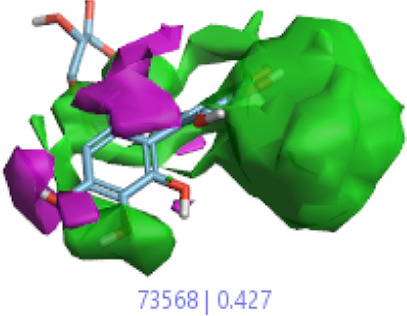	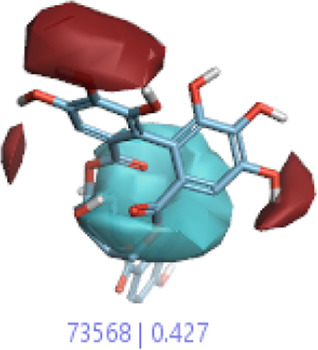	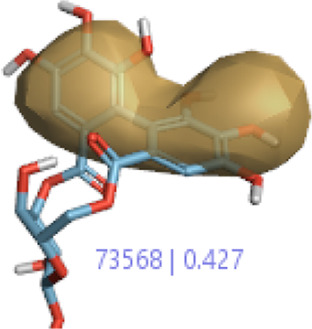	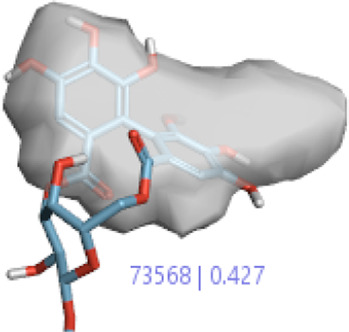
**Ellagic acid (B40)**	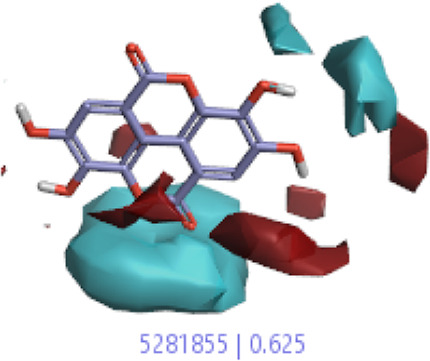	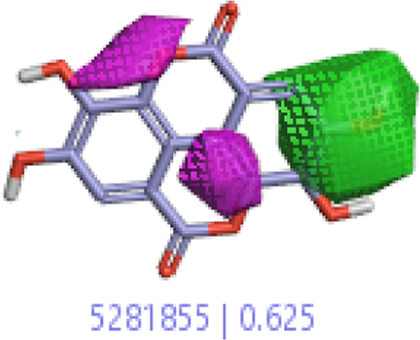	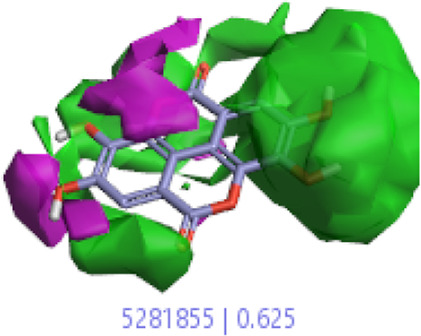	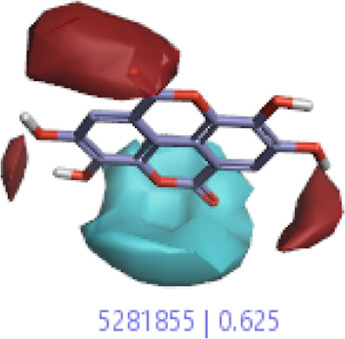	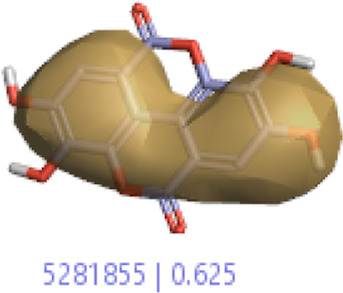	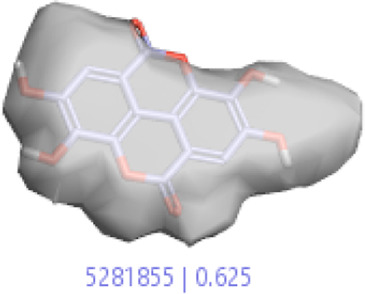
**Kaempferol (B45)**	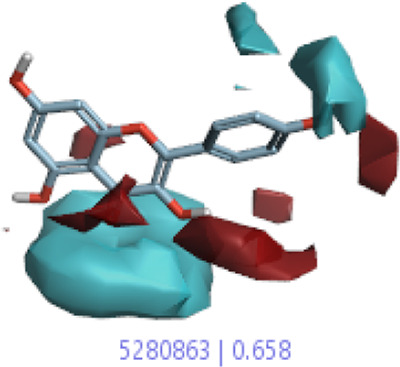	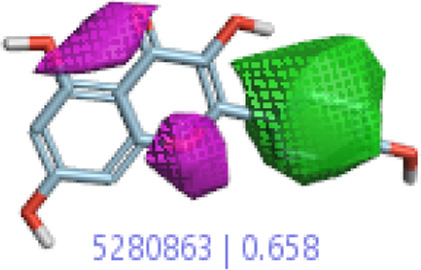	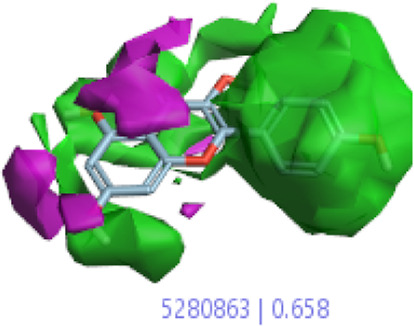	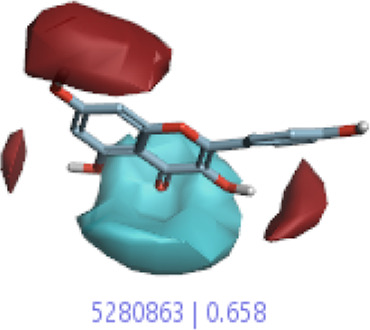	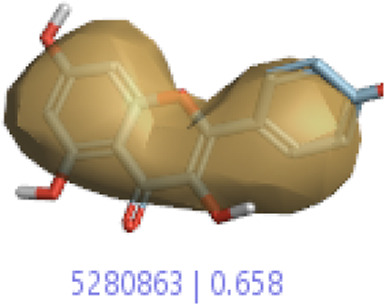	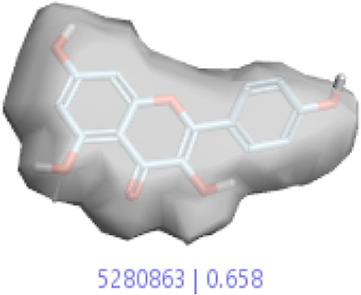
**Myricetin (B46)**	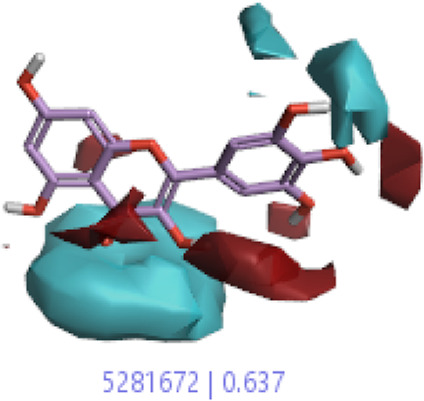	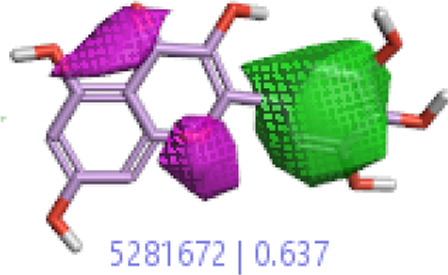	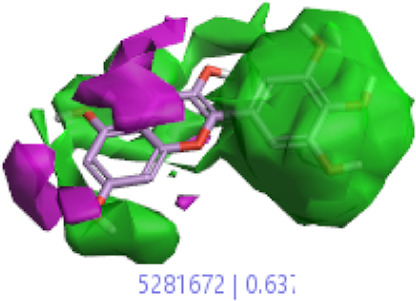	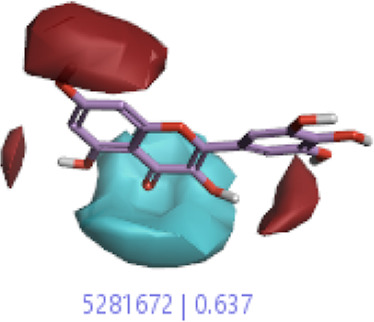	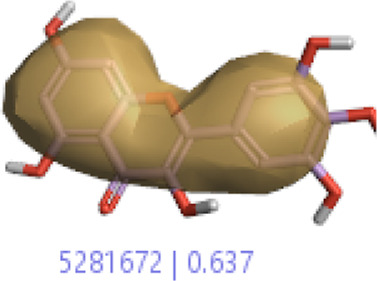	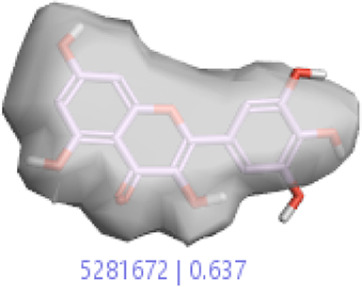
**Luteolin (B48)**	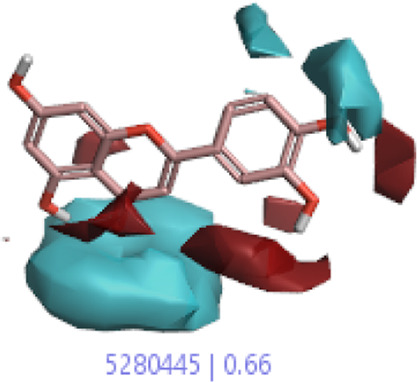	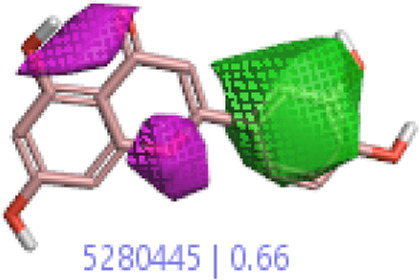	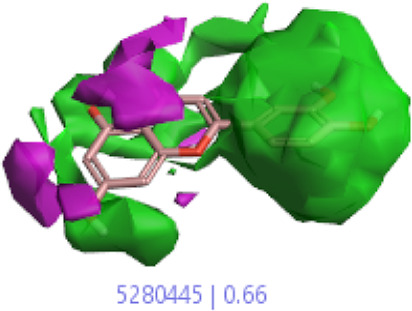	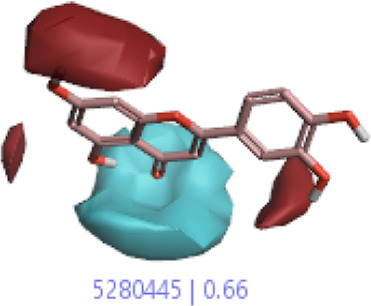	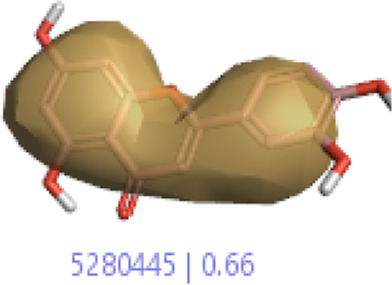	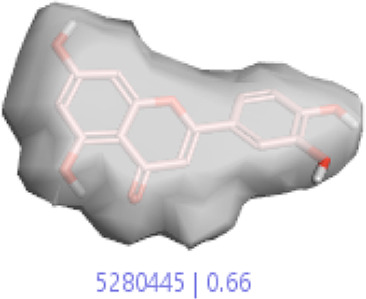
**Taxifolin (B61)**	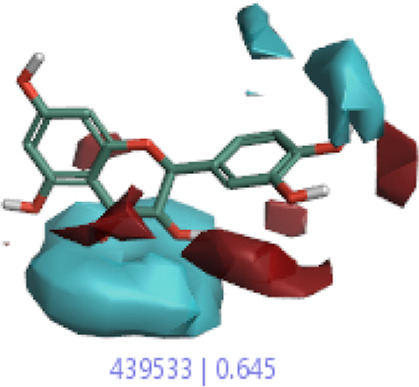	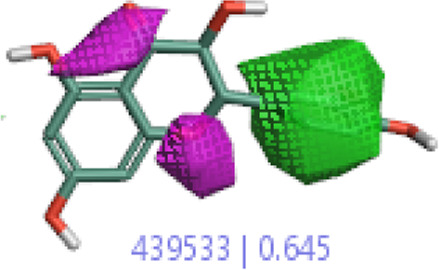	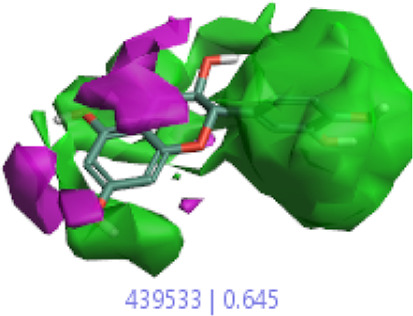	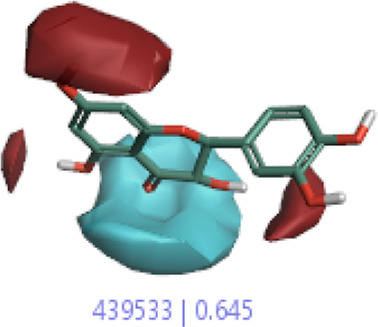	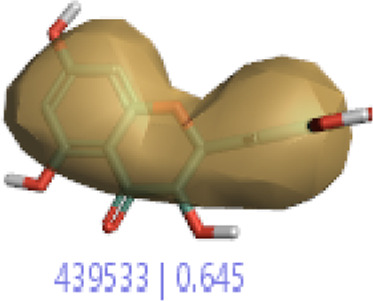	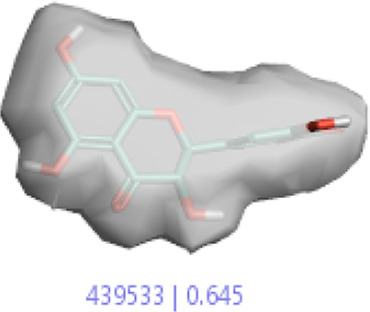
**Quercetin-3-glucoside (B66)**	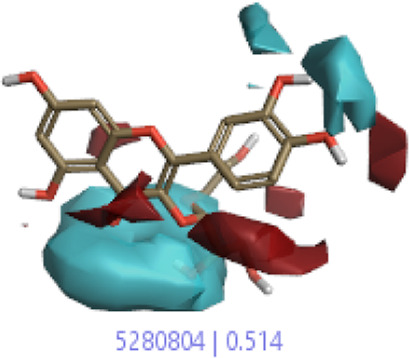	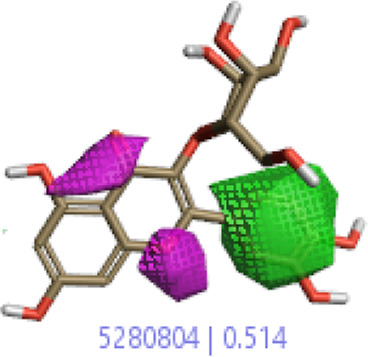	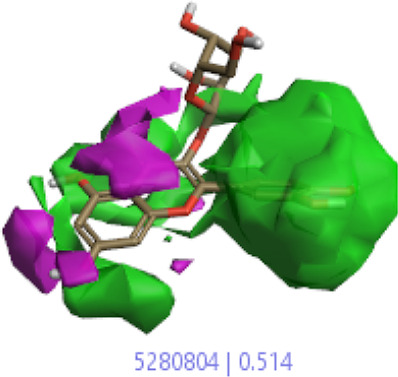	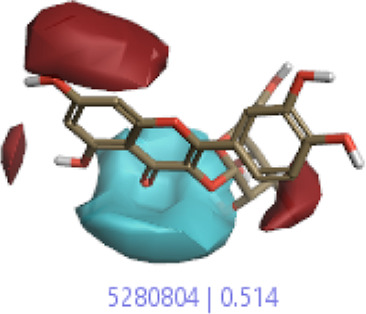	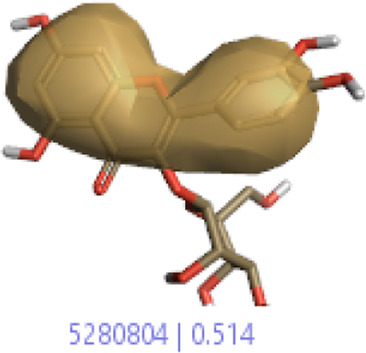	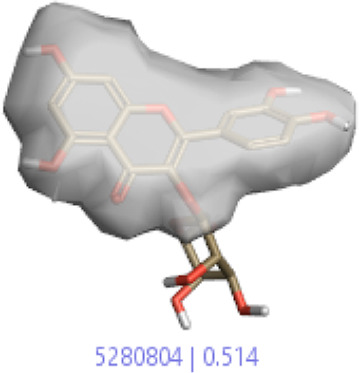

### 3.2 Molecular docking using MVD

All active compounds from the 3D-QSAR model undergo docking by using the Molegro Virtual Docker that gives hit lead compounds. Molecular docking is performed to check the binding mode of the predicted activity of compounds as a pose of the highest rank and with MolDock score in between −60 and −200. It also gives information about proteins and the ligand and binding sites where they attach to inhibit Alzheimer’s disease. Auspicious poses and binding sites are automatically identified by docking of actives.

#### 3.2.1 Protein–ligand interaction

During visualization of the protein–ligand interaction of active compounds, reference drugs show a low MolDock score as compared to other compounds with proteins. For this, interaction proteins are selected against Alzheimer’s disease, such as **7KOQ** from acetylcholine, **5ONP** from beta-amyloid, and **1GWR** and **4AA6** from estrogen hormones.

##### 3.2.1.1 Protein of acetylcholine (Ach) hormone

**FIGURE 5:**
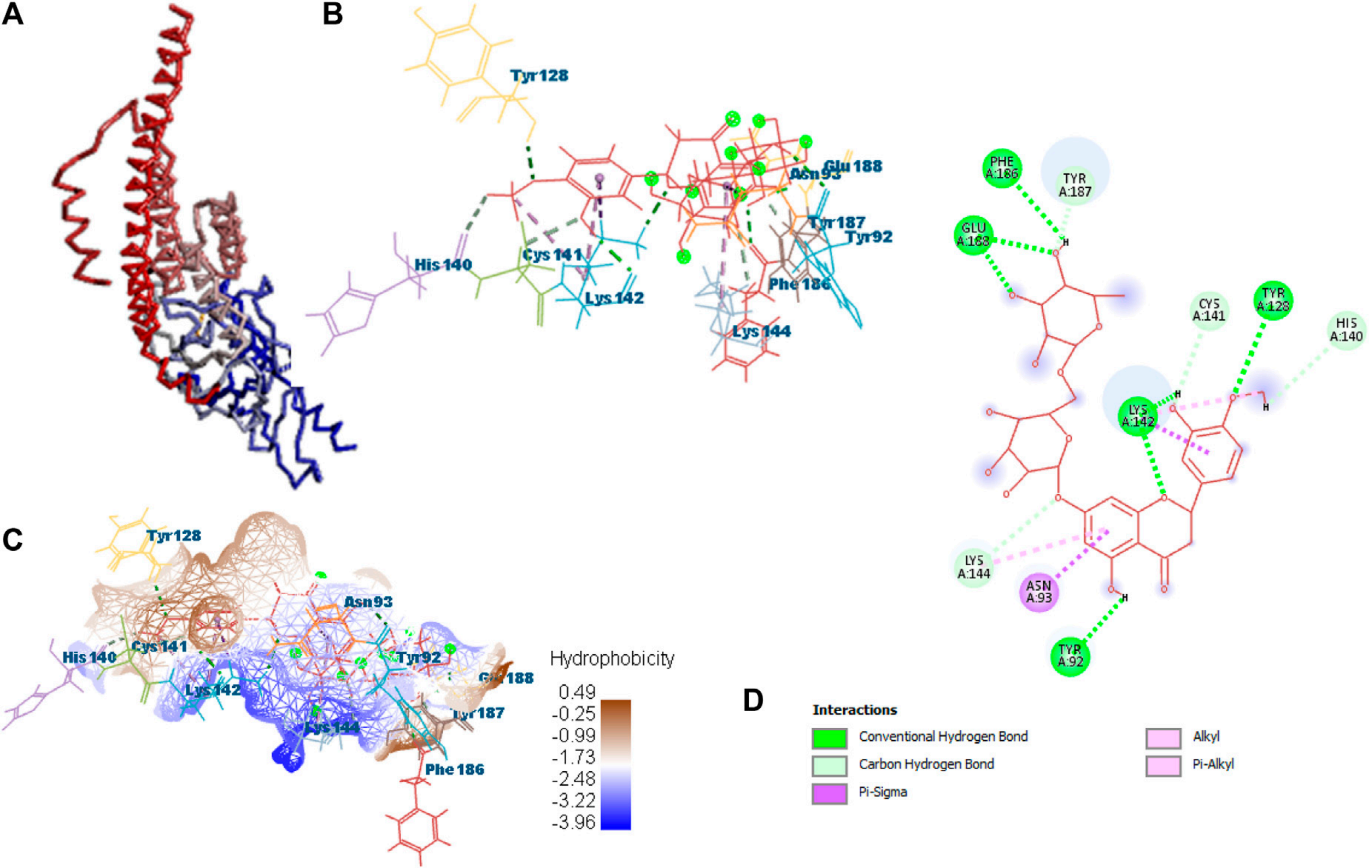
Protein–ligand interaction of B25 with 7KOQ. **(A)** Protein; **(B)** protein–ligand interaction; **(C)** hydrophobicity; and **(D)** 2D diagram.

Docking was performed for all the active ligands, namely, **B25**, **B29**, **B35**, **B40**, **B45**, **B46**, **B48**, **B61**, and **B66,** with protein 7KOQ. **B25** (hesperidin) showed the highest MolDock score of −151.961 in comparison to the reference drug that showed the lowest MolDock score of −86.768 with 16 interactions. Hesperidin (**B25**) showed 11 interactions with hydrogen bonding (TYR128, LYS142, GLU188, GLU128, PHE186, TYR92, LYS142, CYS141, LYS144, TYR187, and HIS140) and five with van der Waal interactions (ASN93, LYS142, LYS142, LYS144, and LYS142). Ligand interaction, hydrophobicity, and 2D structure are shown in Figure 5 (Supplementary Table S2).

##### 3.2.1.2 Protein of beta-amyloid.

**FIGURE 6:**
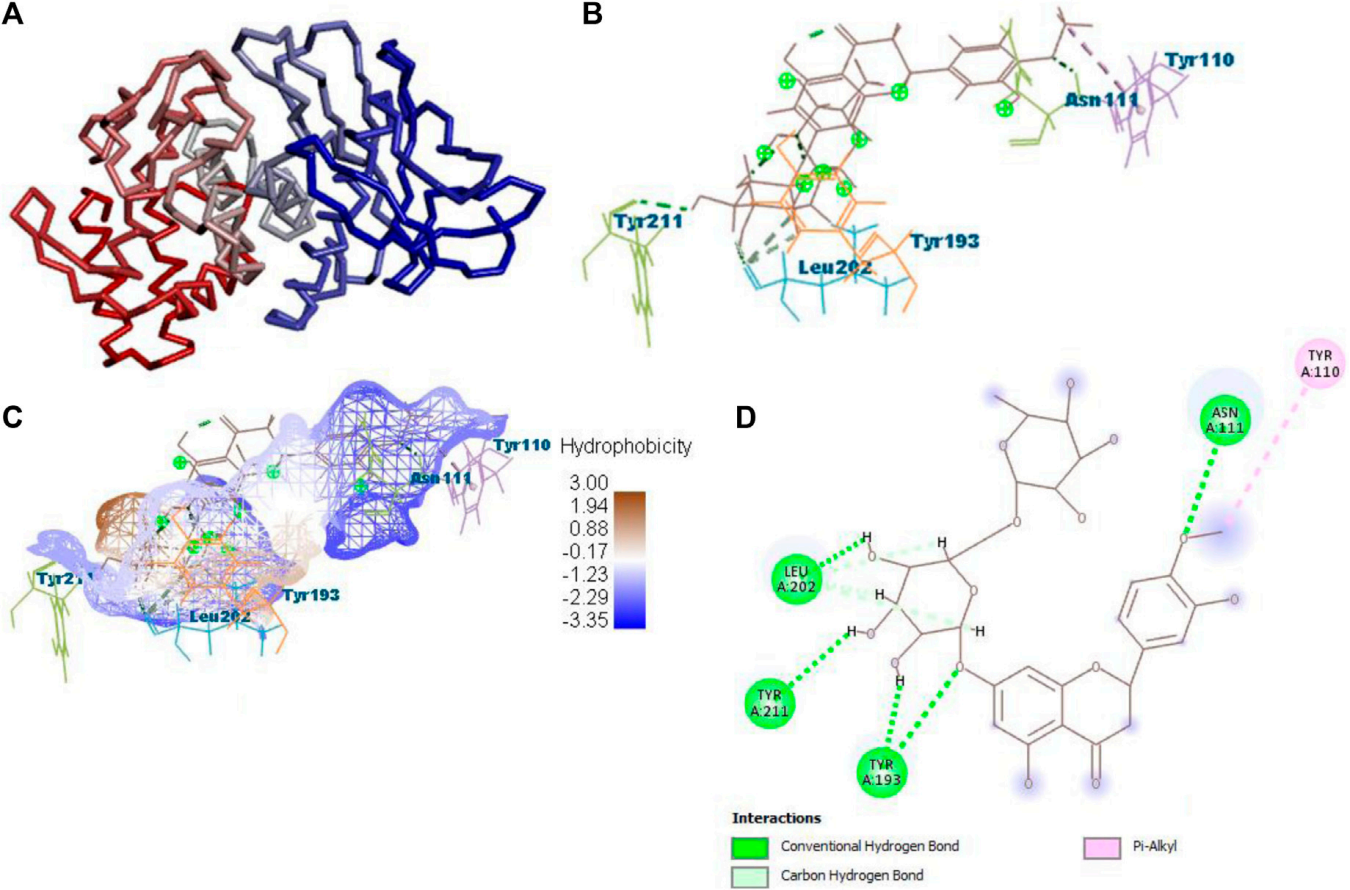
Protein–ligand interaction of B25 with 5ONP. **(A)** Protein; **(B)** protein–ligand interaction; **(C)** hydrophobicity; and **(D)** 2D diagram.

After docking with all active compounds, **B25** was found as a lead compound with the highest MolDock score by interacting with **5ONP** protein. Hesperidin (**B25**) displayed nine interactions with hydrogen bonding, namely, ASN111, TYR193, LEU202, TYR211, TYR193, LIG1:H29, LEU202, LEU202, and LEU202, and one with van der Waals force, namely, TYR110 (Figure 6) (Supplementary Table S2). **B25** also showed the highest MolDock score (−111.712), while the reference drug showed a MolDock score (−66.613) with zero interaction.

##### 3.2.1.3 Proteins of estrogen hormone

**FIGURE 7:**
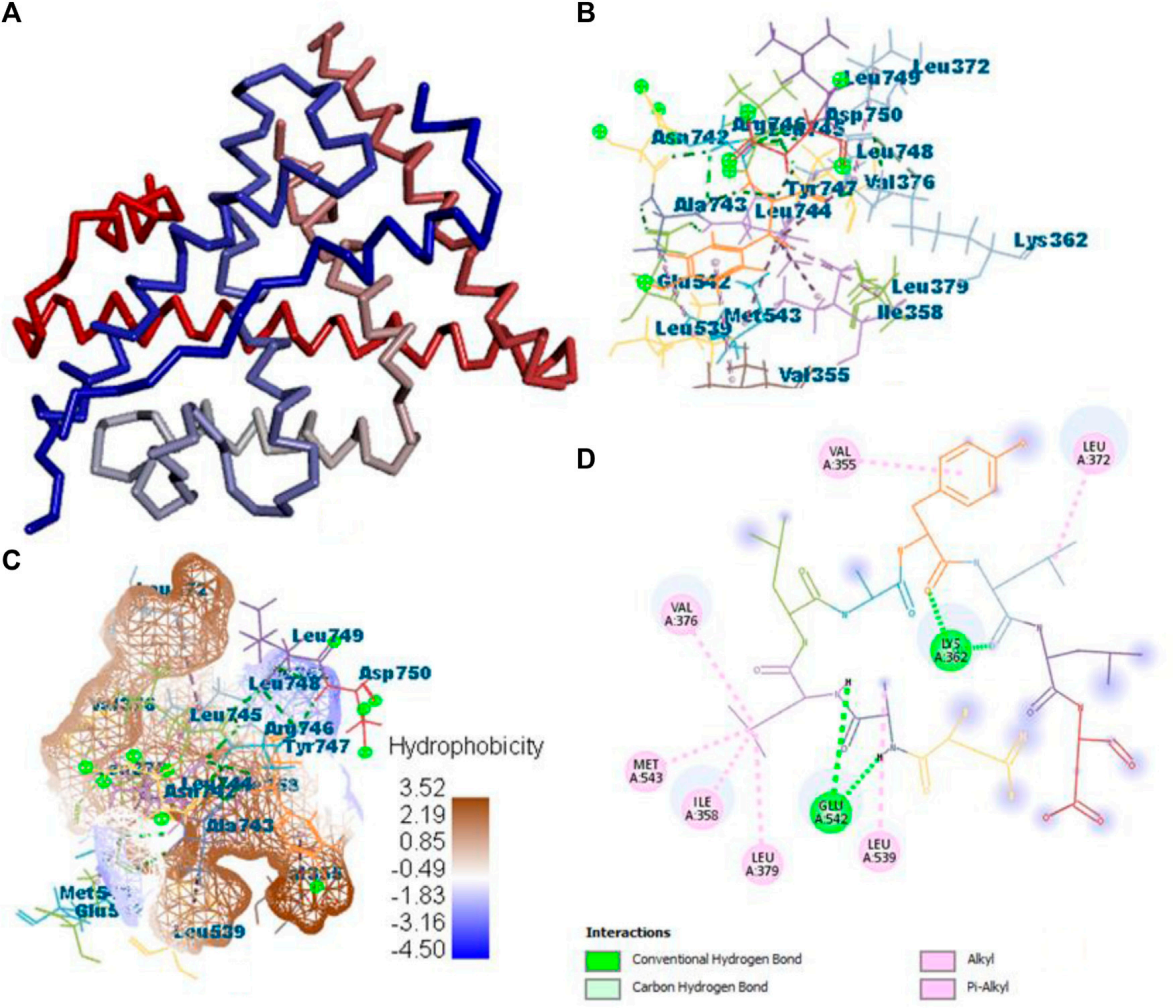
Protein–ligand interaction of B35 with 1GWR. **(A)** Protein; **(B)** protein–ligand interaction; **(C)** hydrophobicity; and **(D)** 2D diagram.

All the active compounds, namely, **B25**, **B29**, **B35**, **B40**, **B45**, **B46**, **B48**, **B61**, and **B66** are docked with **1GWR** protein to get the hit compound. **B35** (corilagin) showed the highest MolDock score (−149.02), while **B25** (hesperidin) showed a little less MolDock score (−144.915) and also a lead compound in comparison to the reference drug (−82.238). Corilagin (B35) compound showed 14 hydrogen interactions, namely, LYS362, LYS362, ALA743, LEU744, ARG746, ARG746, TYR747, TYR747, LEU748, LEU748, LEU749, LEU749, ASP750, and LYS362, and eight interactions with van der Waals forces, namely, ILE358, LEU372, VAL376, LEU379, MET543, ALA743, LEU744, and TYR747 (Figure 7) (Supplementary Table S2).

**FIGURE 8:**
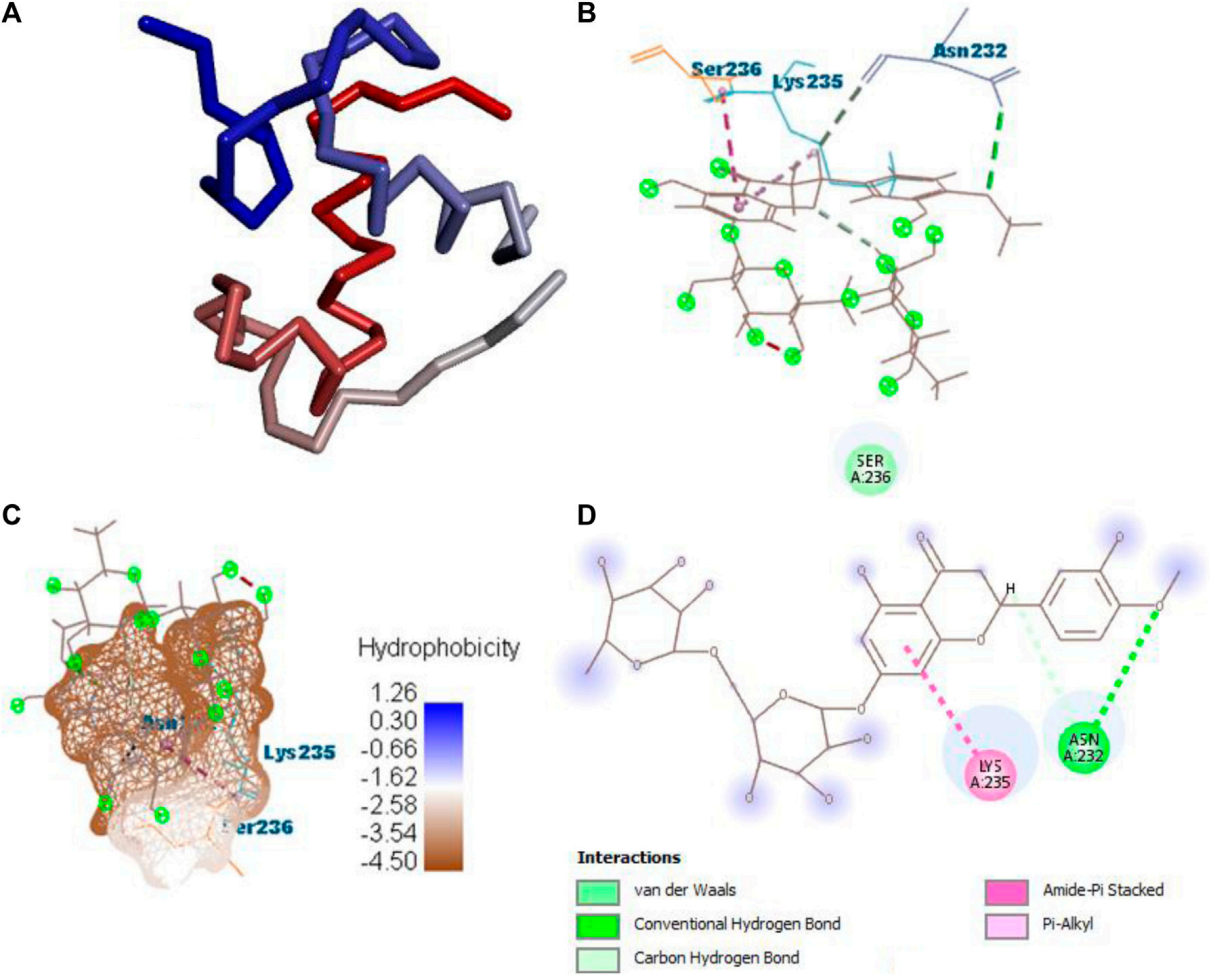
Protein–ligand interaction of B25 with 4AA6. **(A)** Protein; **(B)** protein–ligand interaction; **(C)** hydrophobicity; and **(D)** 2D diagram.

After docking all active compounds with **4AA6**, **B25** (hesperidin) was found to be the hit lead compound with the MolDock score (−154.322), and the reference drug showed a MolDock score (−68.848) with zero interactions. Hesperidin (B25) showed three interactions with hydrogen bonding, namely, ASN232, LIG1:H5, and ASN232, and two with van der Waals interactions, namely, LYS235 and LYS235 (Figure 8) Supplementary Table S2.

After docking all active compounds, hesperidin showed good MolDock scores with 7KOQ, 5ONP, and 4AAP proteins, while corilagin showed the best MolDock score with 1GWR protein against Alzheimer’s disease (Table 4).

**TABLE 4:** Results of molecular docking with MolDock scores and H-bonding.

Compound name	Hesperidin (B25)	Hesperidin (B25)	Corilagin (B35)	Hesperidin (B25)
**Proteins**	7KOQ	5ONP	1GWR	4AA6
**MolDock score**	−151.96	−111.71	−149.02	−154.32
**H-bonding**	−20.801	−10.861	−18.877	−4.997

### 3.3 MD simulation analysis

Molecular dynamics (MD) was performed for checking the stability of protein–ligand complexes, and it also evaluated the ligand-induced modification in the structure of proteins. The root-mean-square fluctuation (RMSF) profile for proteins was created using the iMOD server to check amino acid flexibility. The minimum value of RMSF shows less motion of the system, and the maximum value of RMSF shows more flexibility throughout the MD simulation.

**FIGURE 9:**
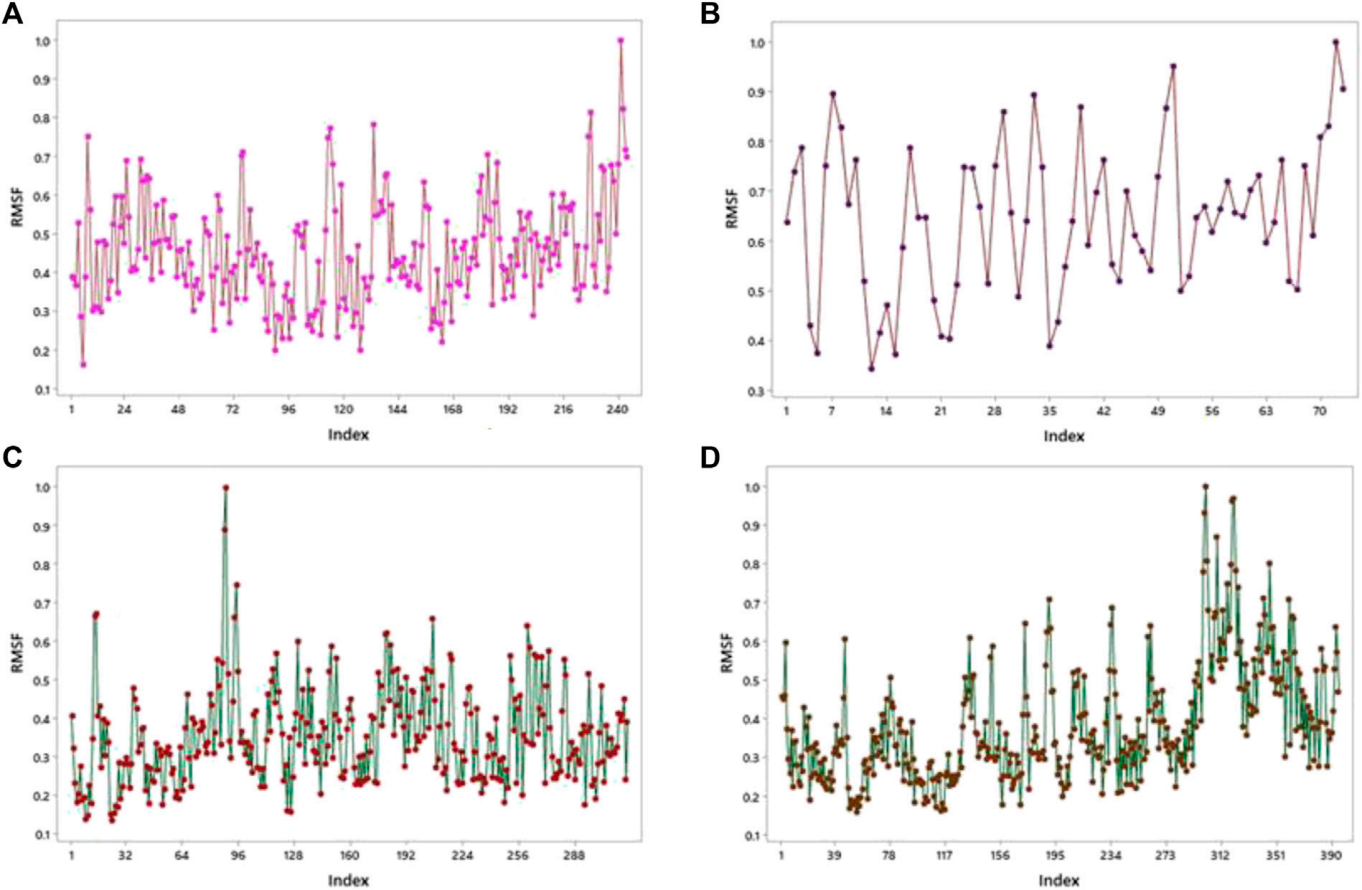
RMSF profiles by MD simulation of **(A)** 1GWR, **(B)** 4AA6, **(C)** 5ONP, and **(D)** 7KOQ.

This server shows the model structure with output files and RMSF in a graph for the calculation of fluctuation in proteins. 1GWR showed the maximum value of fluctuation (8.23057E-01Å) at 242 residue numbers and the minimum value of fluctuation (1.61593E-01Å) at 6 residue numbers (Figure 9A). 4AA6 indicated the maximum value of fluctuation (9.05256E-01Å) at 73 residue numbers and the minimum value of fluctuation (1.00000E-01Å) at 72 residue numbers (Figure 9B). 5ONP exhibits the maximum value of fluctuation (8.88434E-01Å) at 88 residue numbers and the minimum value of fluctuation (1.32875E-01Å) at 24 residue numbers (Figure 9C). 7KOQ demonstrated the maximum value of fluctuation (9.33859E-01Å) at 300 residue numbers, whereas the minimum value of fluctuation (1.56757E-01Å) occurred at 54 residue numbers (Figure 9D).

**FIGURE 10:**
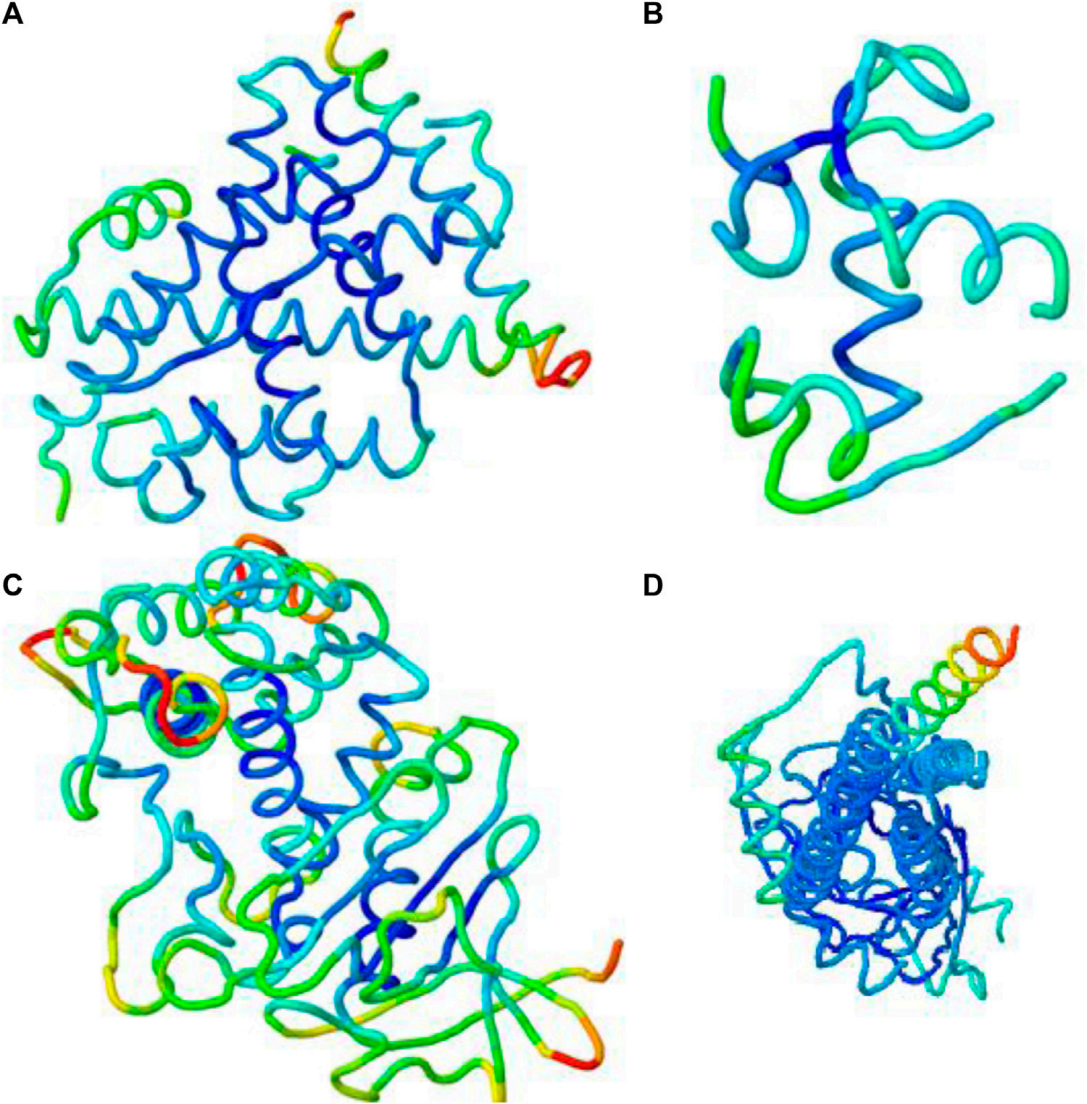
Molecular mobility of docked complexes by NMA. **(A)** 1GWR-corilagin, **(B)** 4AA6-hesperidin, **(C)** 5ONP-hesperidin, and **(D)** 7KOQ-hesperidin complexes.

By using the iMOD server, MD simulation was performed to assess the physical properties and stability of docked compounds. Normal mode analysis (NMA) was applied to check the slow dynamics for docked compounds and the exhibited conformational fluctuations with high amplitude. NMA for these docked complexes, such as the 1GWR-corilagin complex, 4AA6-hesperidin complex, 5ONP-hesperidin complex, and 7KOQ-hesperidin complex, is given in Figure 10.

**FIGURE 11:**
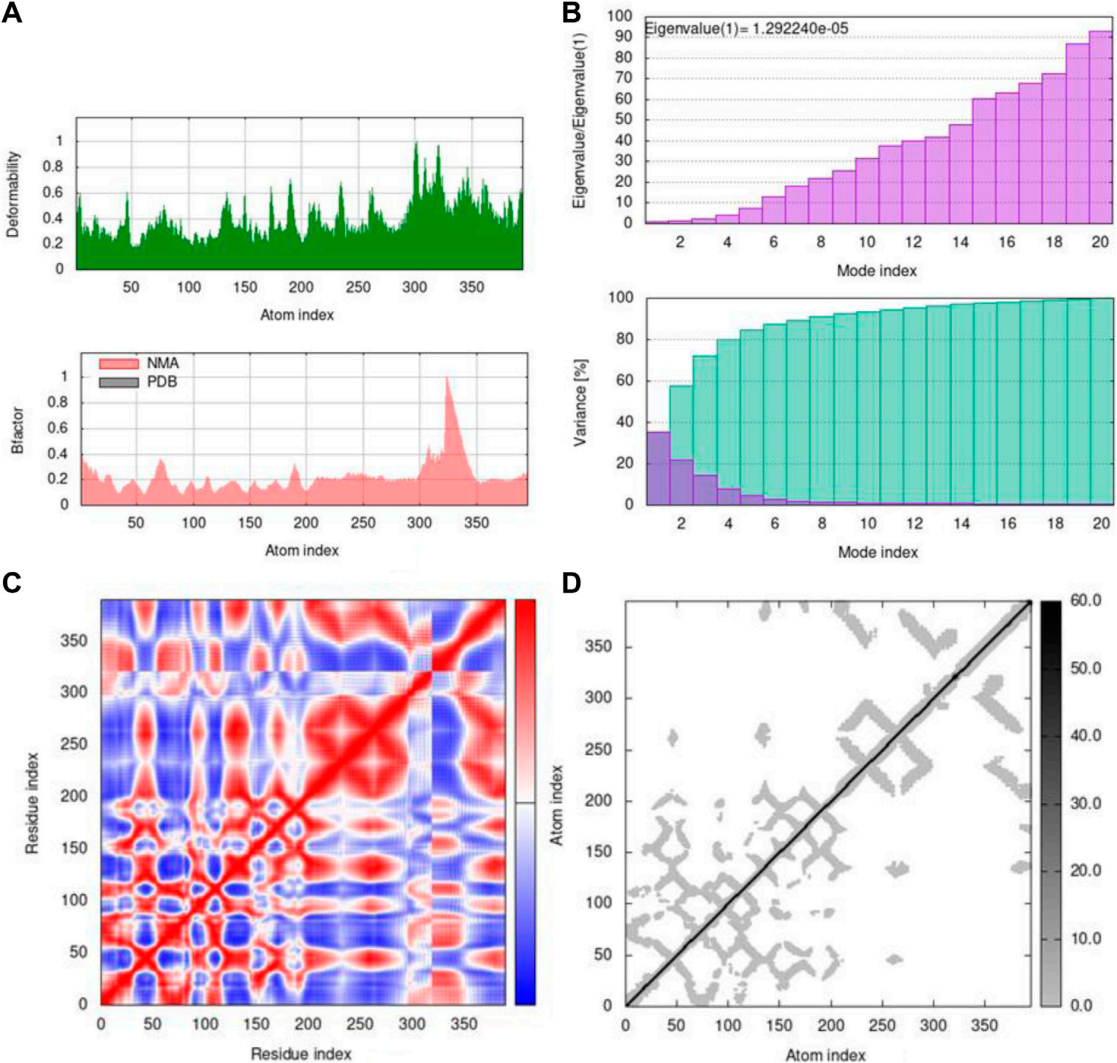
Outputs by MD simulation for 7KOQ: **(A)** B-factor and deformability, **(B)** variance and eigenvalue graph, **(C)** network model, and **(D)** co-variance map.

Deformability indicates the flexibility of proteins, and its main chain is a measure having the capability to deform the given molecule at its each residue. The site of the main chain “hinges” can be regulated from the high region of deformability. The flexibility of proteins is shown by deformability, and the mobility of proteins is shown by B-factor. The peaks that show the hinge regions have higher deformability, as shown in Figure 11A. The B-factor is measured to quantify the uncertainty and flexibility in the proteins. The values of B-factor can be calculated by NMA, and the graph shows a clear visualization of docked complexes between NMA and PDB (López-Blanco et al., 2014). The NMA of proteins depends on the assumption that the low frequencies shown by a vibrational normal mode designate the maximum movement of proteins, which are functionally significant. The NMA of protein–ligand complexes deals with vibrational and dynamic modes rather than colored properties.

Mobility profiles of docked complexes are obtained by B-factor and deformability. The B-factor and deformability of 7KOQ-hesperidin and 5ONP-hesperidin complexes showed peaks that relate to the protein region. The top peaks demonstrated high deformability in that region. The B-factor and deformability graphs for 7KOQ are relatable, having a high peak at approximately residues 310–340, in both the graphs. However, in 5ONP, the deformability graph has a high peak at residues ranging from 80 to 90, while the B-factor graph has a high peak between residues 230 and 300. A high B-factor for a particular region of protein denotes an increase in the mobility of that region. The B-factor and deformability of 7KOQ-hesperidin are shown in Figures 11A,B.

The variance and eigenvalue were inversely related in every mode. The purple-colored bars displayed the individual variance, and the green-colored bars displayed the cumulative variance in the graph of variance of 7KOQ-hesperidin and 5ONP-hesperidin. The eigenvalue of each complex is given in Supplementary Table S3.

**FIGURE 12:**
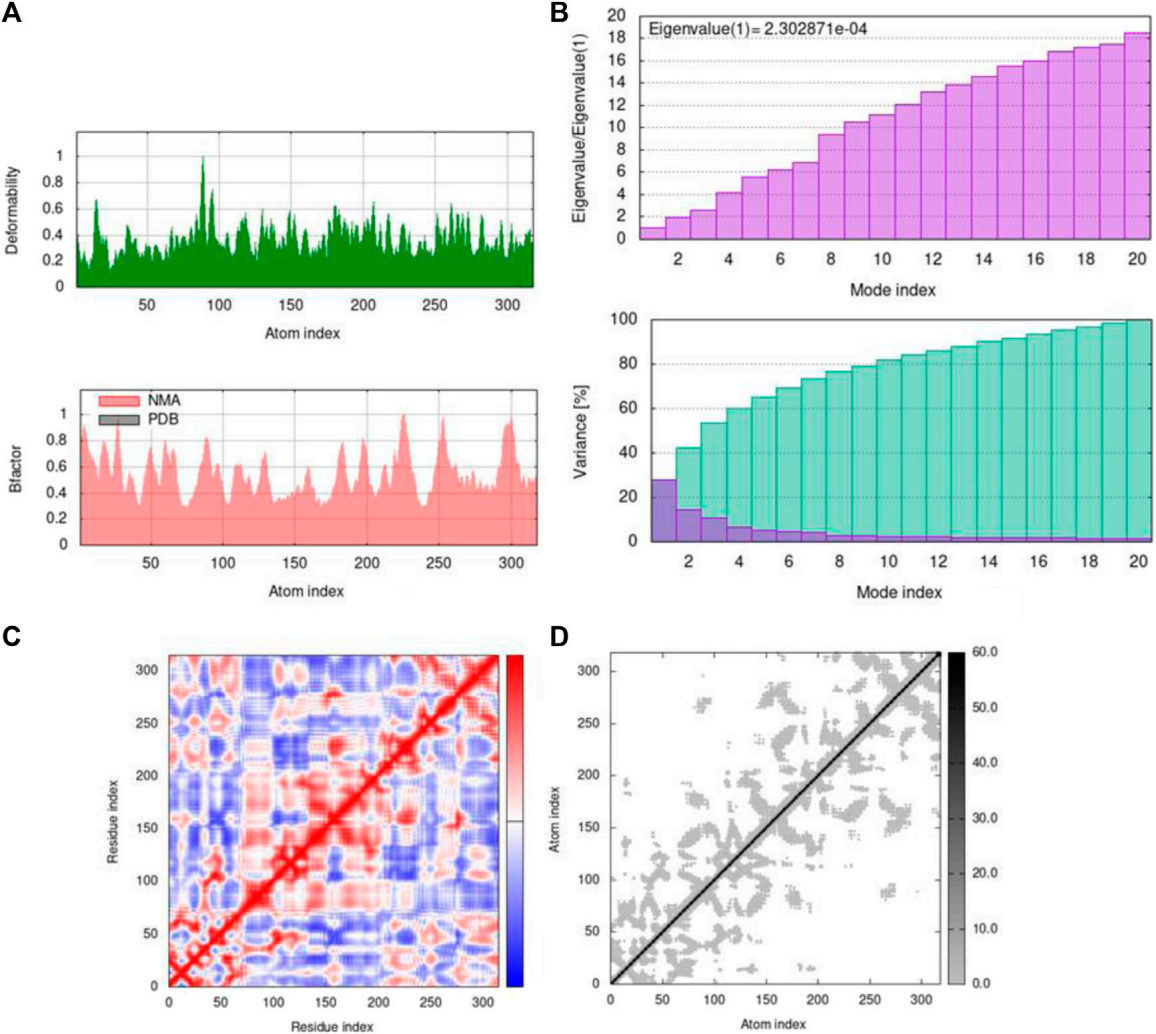
Outputs by MD simulation for 5ONP: **(A)** B-factor and deformability, **(B)** variance and eigenvalue graph, **(C)** network model, and **(D)** co-variance map.

The correlations among the complex residue obtained by the covariance matrix of 7KOQ-hesperidin and 5ONP-hesperidin are shown in Figures 12A,B. A decent correlation is represented by the red color between residues, whereas uncorrelated motion is represented by white color. A high correlation indicated that the complexes were good. The dark gray color in the elastic maps showed that a stiffer portion was specified.

#### 3.3.1 RMSF overlapping

**FIGURE 13:**
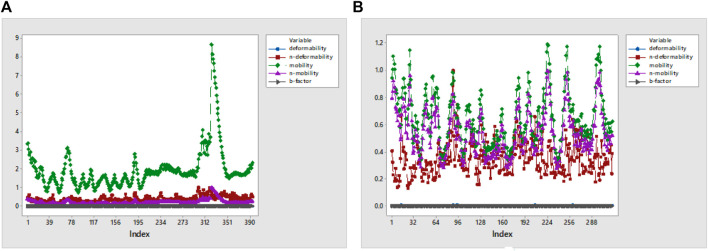
RMSF overlapping graph of **(A)** 7KOQ and **(B)** 5ONP.

RMSF analysis was executed to measure the flexibility of each atom, and very similar fluctuations of complexes appeared that showed high flexibility. In addition, they also demonstrate the interaction of active-site residues in contrast to reference compounds. RMSF overlapping of 7KOQ-hesperidin and 5ONP-hesperidin complexes is shown in Figure 13. 5ONP-hesperidin showed the highest variation among both proteins that indicates greater flexibility.

#### 3.3.2 Root-mean-square deviation (RMSD)

**FIGURE 14:**
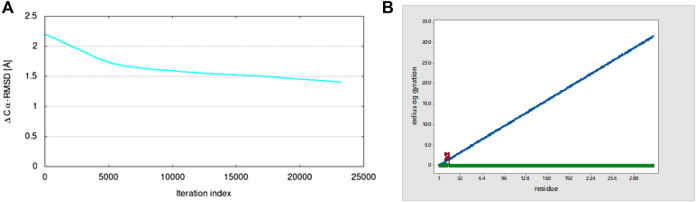
**(A)** Plot of the Cα-RMSD value from iMOD and **(B)** radius of gyration.

RMSD was found to be low in the targeted structure, while the starting structure was deformed through the lowest modes that stimulate the transition. For studying RMSD, the coarse-grained (Cα) backbone atoms of protein–ligand complexes were determined for conformational variations and dynamic stability in the dynamic simulation. The ligand binding at the protein-binding site was shown to be stable and had no effect on the protein’s C-alpha backbone stability. After attaining the lead targeted protein 5ONP chains and intermediate structure, the conformational transition pathways were started. After the calculation is finished, the graph indicates the 1.4 Å Cα-RMSD value, as shown in Figure 14A.

#### 3.3.3 Radius of gyration (Rg)

The radius of gyration (Rg) shows the overall compression of the structure of proteins during molecular dynamics simulation. During a given period of time, it calculates the distance between the terminal and the center of mass of each protein atom. A protein structure’s dynamic stability is determined by a slightly lower change in the radius of gyration, which is also a sign of a stable folded structure of protein. For the 5ONP protein, the graph shows a high value of the radius of gyration of 28.264 at residue number 10, as shown in Figure 14B.

### 3.4 Frontier molecular orbital (FMO) studies

**FIGURE 15:**
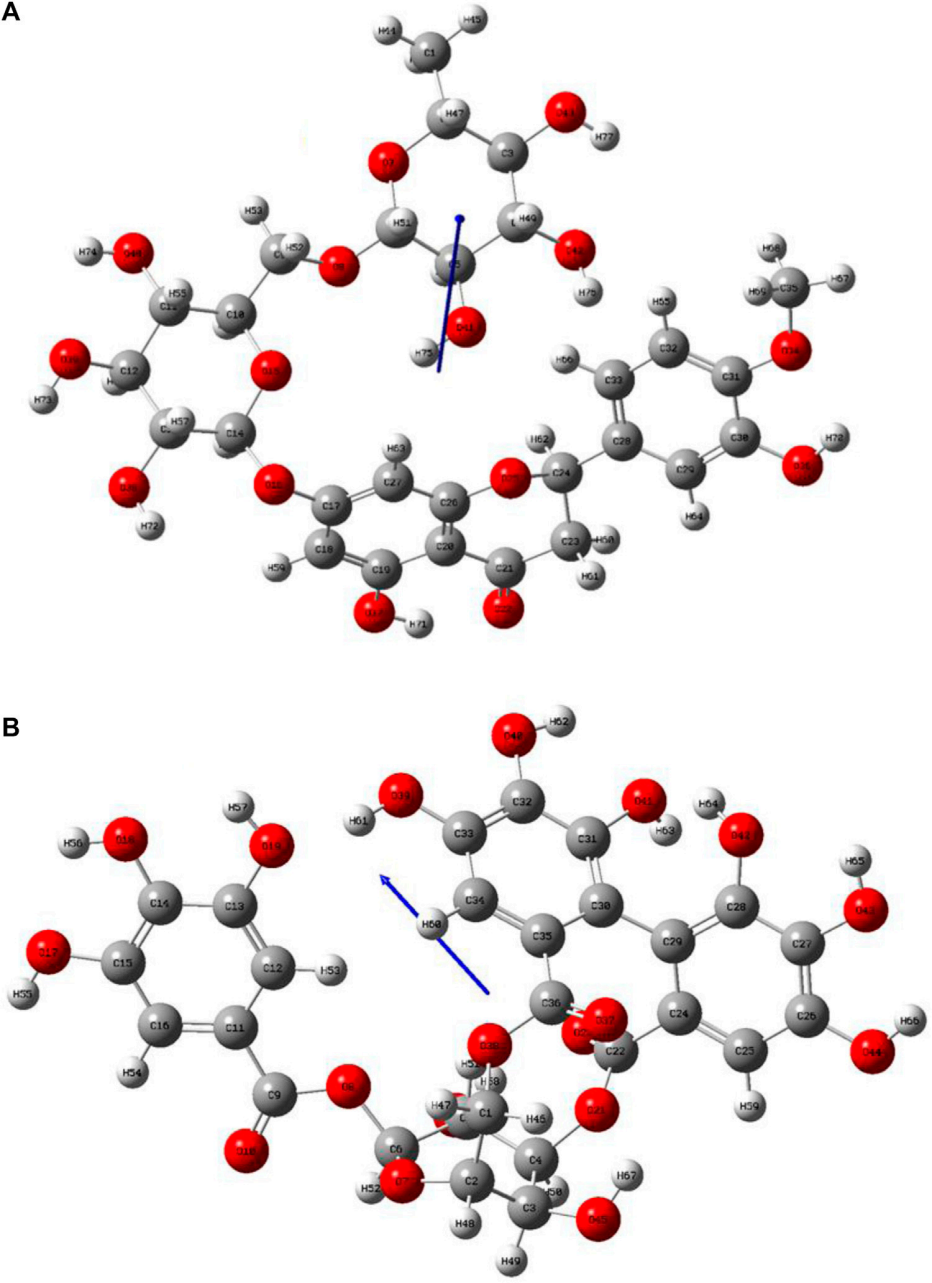
Optimization structure of **(A)** hesperidin and **(B)** corilagin.

Lead compounds from docking **B25** and **B35** undergo further screening by DFT analysis. The highest occupied molecular orbital (HOMO), the lowest unoccupied molecular orbital (LUMO), nucleophilicity, hardness, and softness are directly related to the inhibitors that have the reactive ability. DFT is a theoretical approach that provides precise, fundamental, and important parameter values for extremely complicated molecules at a low cost (Obot et al., 2015). All chemical descriptors in DFT were measured using a basic set (6–311G B3LYP) (Supplementary Table S4). Optimized structures of **B25** and **B35** along with their vectors are shown in Figure 15.

**FIGURE 16:**
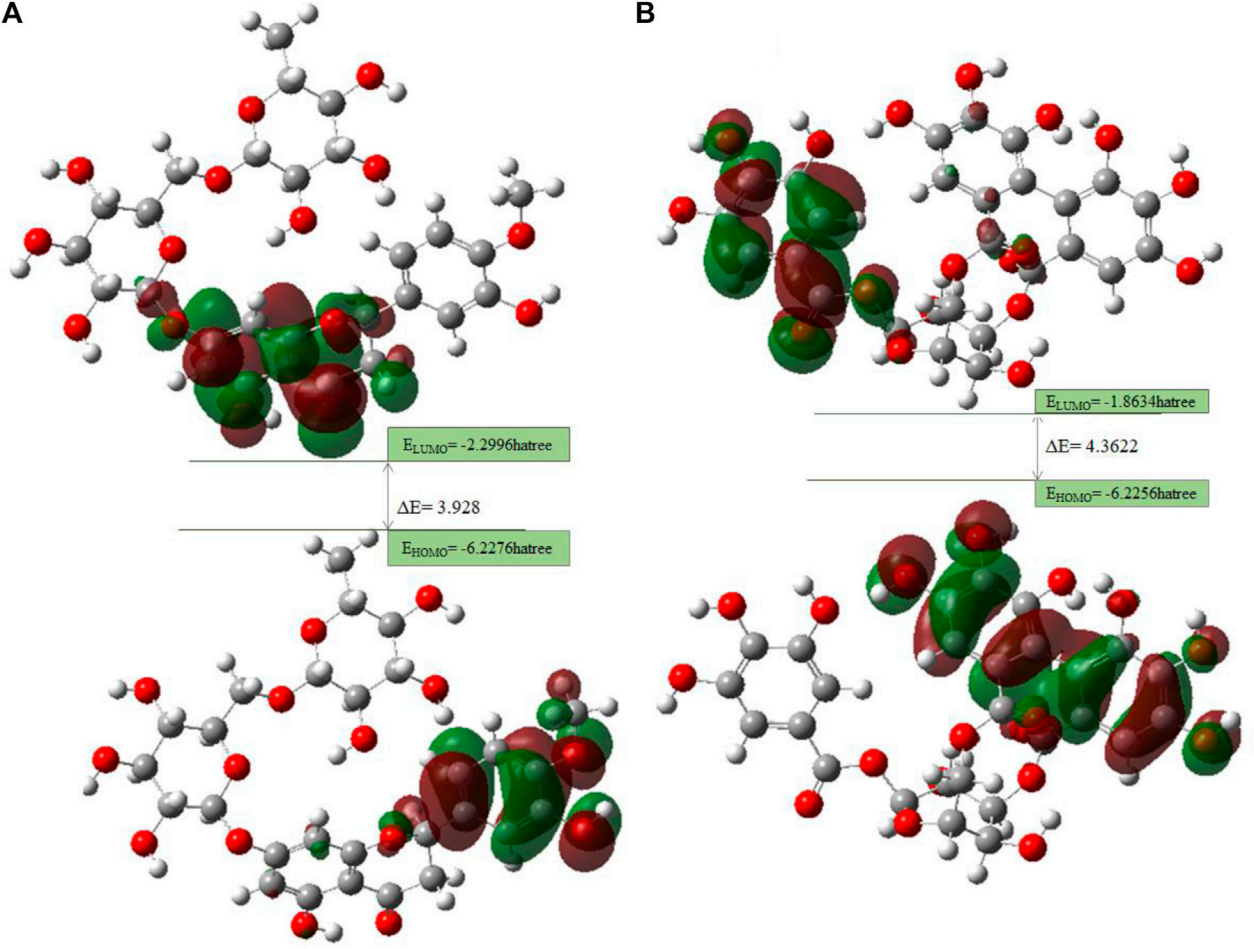
FMO diagrams along with the energy gap: **(A)** hesperidin and **(B)** corilagin.

The HOMO is a strong electron donor that donates electrons that are accepted by the LUMO to increase biological activity. The energy gap between **B25** and **B35** shows the stability of the compound as the gap is smaller than the molecule, which is more stable and reactive chemically (Figure 16.). Based on chemical descriptors, both corilagin and corilagin compounds are ranked as:

Lead compounds from docking **B25** and **B35** undergo further screening by DFT analysis. Both hesperidin and corilagin show good results through DFT optimization. All chemical descriptors in DFT were measured using the basic set (6–311G B3LYP) Supplementary Table S4.

#### 3.4.1 MEP analysis

**FIGURE 17:**
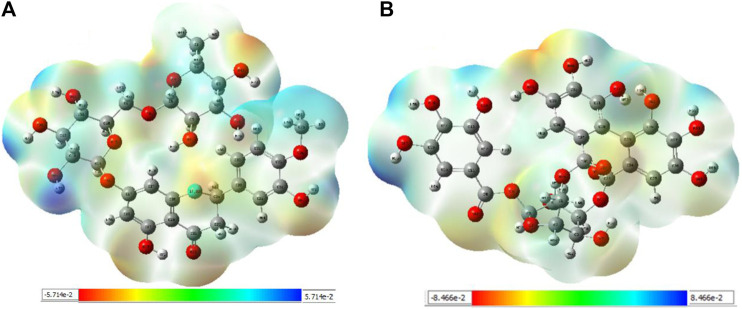
MEP structure and scale: **(A)** hesperidin and **(B)** corilagin.

Interpretations about nucleophilicity, electrophilicity, hydrogen-bond interaction, and drug reactivity are predicted by molecular electrostatic potential studies. This model is used to determine the behavior and response of molecules toward a binding substrate with other compounds. In addition, the relative polarity of molecules was also checked through the visual scheme. In the MEP scale, the positive region of electrostatic potential in the blue color shows the nucleophilic site, and the negative region of electrostatic potential in red and yellow colors shows the electrophilic site. The blue color shows the site of nucleophilic attacks where hydrogen atoms are capable of reacting with the nucleophile. The red and yellow colors show the site of electrophilic attack where oxygen atoms are capable of reacting with the electrophile. However, the green color shows the reactivity where the benzene ring remains neutral. The results show that **B25** is the hit leading compound that undergoes ADMET studies. The MEP structure and scales of **B25** and **B35** compounds are shown in Figure 17.

### 3.5 ADME toxicity

A sufficient drug quantity must be distributed to the target area with fewer side effects and toxicity for a medicine to be considered an effective quality one. Pharmaceutical companies select important ligands for experimentation based on ADME toxicity analysis by envisaging pharmacokinetic and physicochemical qualities before starting an expensive clinical study (Li, 2001). So, ADMET is performed against lead compounds from DFT to check their toxicity for the safety of the drug by using the ProTox-II online server. Table 5 shows that hesperidin **(B25)** does not affect cytotoxicity, hepatotoxicity, and carcinogenicity.

**TABLE 5:** Toxicity analysis of lead compounds.

Sr. no.	Compound name	Hepatotoxicity	Carcinogenicity	Mutagenicity	Cytotoxicity
**1.**	**Hesperidin**	**-(0.81)**	**-(0.93)**	**-(0.90)**	**-(0.52)**

## 4 Conclusion

Of all the active compounds, hesperidin **(B25)** is the hit lead compound from the phytochemical screening of *P. granatum* against antioxidant activity to inhibit Alzheimer’s disease by the hormones acetylcholine (Ach) and estrogen (beta-amyloid). The 3D-QSAR model gives us information about the structure–activity relationship of screened phytochemicals and field-point generation that are generated by the alignment of the training and test set compounds. The nine active compounds, namely, **B25**, **B29**, **B35**, **B40**, **B45**, **B46**, **B48**, **B61**, **and B66**, are screened from this model and can be visualized by the activity atlas model. These active compounds undergo further screening by molecular docking with four proteins **(7KOQ**, **5ONP**, **1GWR**, and **4AA6)** using Molegro Virtual Docker software. We obtained two lead compounds **(B25** and **B35)** with the best MolDock score along with hydrophobicity and ligand interaction. DFT gives us the optimized structure, chemical reactivity descriptors, and molecular electrostatic potential structure of active compounds. So, DFT is performed with these lead compounds to obtain the one-hit compound, and the low energy gap shows that the **B25** compound is more chemically reactive because of its stability. In the end, ADMET was performed for the **B25** compound to check its toxicity and efficiency against the inhibition of Alzheimer’s disease. **Hesperidin (B25)** is the best hit-lead compound for drug repurposing against Alzheimer’s disease with zero toxicity.

### 5 Future perspectives

The hesperidin compound is screened from the library of the *P. granatum* plant against antioxidant actions. Hesperidin is a bioflavonoid compound studied for pharmacological actions and several health diseases. *In silico* studies of hesperidin can increase the chances of drug discovery for antioxidant activity against Alzheimer’s disease. The hesperidin (**B25**) compound will be used as a drug with zero toxicity in the future. Furthermore, retrosynthesis and synthesis of hesperidin can be performed for effective drug discovery (Binkowska, 2020).

## Data Availability

The original contributions presented in the study are included in the article/Supplementary Material; further inquiries can be directed to the corresponding author.
