# Supplementary material for: Developmental landscape of computational techniques to explore the potential phytochemicals from Punica granatum peels for their antioxidant activity in Alzheimer’s disease
Source: Front Mol Biosci. 2023 Oct 11;10:1252178. doi: 10.3389/fmolb.2023.1252178 (PMC10598865; doi:10.3389/fmolb.2023.1252178)
Supplement: Supplementary file 1 [file DataSheet1.docx]

**Table S1:** Screened Phytochemicals from *Punica granatum* with their compound IDs, structures, IC_50_, sources and references

| **Sr. No.** | **Compound**  **name** | **Conical smiles** | **Compound structure** | **Compound ID** | **Molecular weight** | **Sources** | **IC_50_**  **values** | **References** |
| --- | --- | --- | --- | --- | --- | --- | --- | --- |
| **1.** | **Gallic acid**  **(B1)** | C1=C(C=C(C(=C1O)O)O)C(=O)O |  | 370 | 170.12 | Green Tea | 2.2 μM | (Das *et al.*, 2021, Badhani *et al.*, 2015, Padumadasa *et al.*, 2016). |
| **2.** | **Catechin**  **(B2)** | C1C(C(OC2=CC(=CC(=C21)O)O)C3=CC(=C(C=C3)O)O)O |  | 9064 | 290.27 | Green Tea | 0.8µM | (Das *et al.*, 2021, Munteanu and Apetrei, 2022, Ahmadi *et al.*, 2020). |
| **3.** | **Epicatechin**  **(B3)** | C1C(C(OC2=CC(=CC(=C21)O)O)C3=CC(=C(C=C3)O)O)O |  | 72276 | 290.27 | Green Tea | 1.8 µM | (Das *et al.*, 2021, Jug *et al.*, 2021, Kamiyama *et al.*, 2010). |
| **4.** | **Quercetin**  **(B4)** | C1=CC(=C(C=C1C2=C(C(=O)C3=C(C=C(C=C3O2)O)O)O)O)O |  | 5280343 | 302.23 | *Coleus tuberosus* | 0.5 µg/mL | (Das *et al.*, 2021, Xu *et al.*, 2019, Yang *et al.*, 2008, Zuo *et al.*, 2011). |
| **5.** | **Rutin**  **(B5)** | CC1C(C(C(C(O1)OCC2C(C(C(C(O2)OC3=C(OC4=CC(=CC(=C4C3=O)O)O)C5=CC(=C(C=C5)O)O)O)O)O)O)O)O |  | 5280805 | 610.5 | human pyruvate kinase isoenzyme erM2 | 7.80 µM | (Das *et al.*, 2021, Yang *et al.*, 2008, Girsang *et al.*, 2020). |
| **6.** | **Flavonols**  **(B6)** | C1=CC=C(C=C1)C2=C(C(=O)C3=CC=CC=C3O2)O |  | 11349 | 238.24 | human pyruvate kinase isoenzyme M2 | 1 µM | (Magangana *et al.*, 2020, Pietta, 2000). |
| **7.** | **Flavones**  **(B7)** | C1=CC=C(C=C1)C2=CC(=O)C3=CC=CC=C3O2 |  | 10680 | 222.24 | Berry & citrus | 0.17 µM | (Magangana *et al.*, 2020, Pietta, 2000). |
| **8.** | **Proanthocyanidins**  **(B8)** | C1C(C(OC2=CC(=CC(=C21)O)O)C3=CC(=C(C=C3)O)O)OC4(C(C(C5=C(C=C(C=C5O4)O)O)O)O)C6=CC(=C(C=C6)O)O |  | 107876 | 594.5 | Litchi *(Litchi chinensis Sonn.)* | 1.88 ± 0.01 μg/ml | (Magangana *et al.*, 2020, Pietta, 2000, Padumadasa *et al.*, 2016). |
| **9.** | **Anthocyanidins**  **(B9)** | C1=CC=C(C=C1)C2=[O+]C3=CC=CC=C3C=C2 |  | 145858 | 207.25 | Diabetes Mellitus | 4.11 µM | (Magangana *et al.*, 2020, Garcia and Blesso, 2021). |
| **10.** | **Saponins**  **(B10)** | CC1(C2CCC3(C(C2(CCC1OC4C(C(C(CO4)OC5C(C(C(CO5)O)O)O)OC6C(C(C(C(O6)CO)O)O)O)OC7C(C(C(C(O7)CO)O)O)OC8C(C(C(C(O8)CO)O)O)O)C)CCC91C3(CC(C2(C9CC(CC2)(C)C=O)CO1)O)C)C)C |  | 198016 | 1223.3 | *Terminalia schimeperiana* | 2.273± 0.036 µg/mL | (Das *et al.*, 2021, Chen *et al.*, 2014). |
| **11.** | **Triterpenoid**  **(B11)** | CC1(CCC2(CCC3(C(=CCC4C3(CCC5C4(CCC(C5(C)COS(=O)(=O)O)O)C)C)C2C1)C)C(=O)O)C |  | 451674 | 552.8 | *Dracocephalum heterophyllum Benth.* | 0.47 ± 0.18 µg/mL | (Das *et al.*, 2021, Nzogong *et al.*, 2018). |
| **12.** | **Ascorbic acid**  **(B12)** | C(C(C1C(=C(C(=O)O1)O)O)O)O |  | 54670067 | 176.12 | Hydroethanolic  Extract of *ruellia tuberosa L,* | 3.18 μg/mL | (Das *et al.*, 2021, Gęgotek and Skrzydlewska, 2022). |
| **13.** | **Cinnamic acid**  **(B13)** | C1=CC=C(C=C1)C=CC(=O)O |  | 444539 | 148.16 | mushroom *(Agaricus bisporus)* tyrosinase | 2.10 µM | (Singh *et al.*, 2018, Sova, 2012, Peperidou *et al.*, 2017). |
| **14.** | **Protocatechuic acid**  **(B14)** | C1=CC(=C(C=C1C(=O)O)O)O |  | 72 | 154.12 | bee products | 0.134 ± 0.005 µg/mL | (Yassin *et al.*, 2021, Zhang *et al.*, 2021). |
| **15.** | **Pyrogallol**  **(B15)** | C1=CC(=C(C(=C1)O)O)O |  | 1057 | 126.11 | Acethylcholinesterase inhibitory | 10.22 µM | (Shalaby *et al.*, 2019, Sarikaya, 2015). |
| **16.** | **4-Amino benzoic acid**  **(B16)** | C1=CC(=CC=C1C(=O)O)N |  | 978 | 137.14 | tyrosinase | 3.8 µM | (Shalaby *et al.*, 2019, Akberova *et al.*, 201). |
| **17.** | **Vanillic acid**  **(B17)** | COC1=C(C=CC(=C1)C(=O)O)O |  | 8468 | 168.15 | Taiwan | 26µg/mL | (Singh *et al.*, 2018, Kumar *et al.*, 2011). |
| **18.** | **Catechol**  **(B18)** | C1=CC=C(C(=C1)O)O |  | 289 | 110.11 | PC-12 cells | 3.0±0.8 µM | (Singh *et al.*, 2018, Smolyaninov *et al.*, 2022). |
| **19.** | **p-Hydroxybenzoic acid**  **(B19)** | C1=CC(=CC=C1C(=O)O)O |  | 135 | 138.12 | Zinc(II) mineral | 15.1 µΜ | (Singh *et al.*, 2018, Rani *et al.*, 2018, Tshane *et al.*, 2021). |
| **20.** | **Ferulic acid**  **(B20)** | COC1=C(C=CC(=C1)C=CC(=O)O)O |  | 445858 | 194.18 | Sugar date palm *[Phoenix sylvestris (L.) Roxb.]* | 0.52 μM | (Singh *et al.*, 2018, Rani *et al.*, 2018, Ergün *et al.*, 2011). |
| **21.** | **Iso-ferulic acid**  **(B21)** | COC1=C(C=C(C=C1)C=CC(=O)O)O |  | 736186 | 194.18 | *Rhizoma Cimicifugae* | 1.08±0.01 µg/mL | (Singh *et al.*, 2018, Wang *et al.*, 2011). |
| **22.** | **Rosmarinic acid**  **(B22)** | C1=CC(=C(C=C1CC(C(=O)O)OC(=O)C=CC2=CC(=C(C=C2)O)O)O)O |  | 5281792 | 360.3 | MARK4 Inhibition | 6.20 µM | (Dawi *et al.*, 2021, Adomako-Bonsu *et al.*, 2017, Zhu *et al.*, 2014). |
| **23.** | **Benzoic acid**  **(B23)** | C1=CC=C(C=C1)C(=O)O |  | 243 | 122.12 | Sugar date palm *[Phoenix sylvestris (L.) Roxb.]* | 2.74 µM | (Shalaby *et al.*, 2019, Natella *et al.*, 1999). |
| **24.** | **Salicylic acid**  **(B24)** | C1=CC=C(C(=C1)C(=O)O)O |  | 338 | 138.12 | mouse | 0.1 µM | (Shalaby *et al.*, 2019, Islam *et al.*, 2021, Alrashdi *et al.*, 2017). |
| **25.** | **Hesperidine**  **(B25)** | CC1C(C(C(C(O1)OCC2C(C(C(C(O2)OC3=CC(=C4C(=O)CC(OC4=C3)C5=CC(=C(C=C5)OC)O)O)O)O)O)O)O)O |  | 10621 | 610.6 | human pyruvate kinase isoenzyme M2 | 17.30 µM | (Shalaby *et al.*, 2019, Parhiz *et al.*, 2015, Kalpana *et al.*, 2009). |
| **26.** | **Naringenin**  **(B26)** | C1C(OC2=CC(=CC(=C2C1=O)O)O)C3=CC=C(C=C3)O |  | 932 | 272.25 | *Citrus maxima Merr.* | 0.24 µM | (Farag *et al.*, 2020, Cavia-Saiz *et al.*, 2010, Brodowska *et al.*, 2016). |
| **27.** | **Hesperitin**  **(B27)** | COC1=C(C=C(C=C1)C2CC(=O)C3=C(C=C(C=C3O2)O)O)O |  | 72281 | 302.28 | *Citrus aurantium L.* | 0.28 µM | (Shalaby *et al.*, 2019, Parhiz *et al.*, 2015). |
| **28.** | **Rhamnetin**  **(B28)** | COC1=CC(=C2C(=C1)OC(=C(C2=O)O)C3=CC(=C(C=C3)O)O)O |  | 5281691 | 316.26 | *Guiera senegalensis* | 0.7 μM | (Shalaby *et al.*, 2019, Park *et al.*, 2014). |
| **29.** | **Apigenin**  **(B29)** | C1=CC(=CC=C1C2=CC(=O)C3=C(C=C(C=C3O2)O)O)O |  | 5280443 | 270.24 | *Acacia auriculiformis A. Cunn. ex Benth.* | 0.14 µM | (Singh *et al.*, 2018, Tian *et al.*, 2021). |
| **30.** | **Syringic acid**  **(B30)** | COC1=CC(=CC(=C1O)OC)C(=O)O |  | 10742 | 198.17 | human pyruvate kinase isoenzyme M2 | 13.8 µM | (Singh *et al.*, 2018). |
| **31.** | **Sinapic acid**  **(B31)** | COC1=CC(=CC(=C1O)OC)C=CC(=O)O |  | 637775 | 224.21 | Brassicaceae family | 3.80 µM | (Dawi *et al.*, 2021, Gaspar *et al.*, 2010). |
| **32.** | **Castalagin**  **(B32)** | C1C2C(C3C4C(C5=C(C(=C(C(=C5C(=O)O4)C6=C(C(=C(C(=C6C(=O)O3)C7=C(C(=C(C=C7C(=O)O2)O)O)O)O)O)O)O)O)O)O)OC(=O)C8=CC(=C(C(=C8C9=C(C(=C(C=C9C(=O)O1)O)O)O)O)O)O |  | 168165 | 934.6 | Vertebrate Squalene Epoxidase | 30 µM | (Singh *et al.*, 2018). |
| **33.** | **Casuarinin**  **(B33)** | C1C(C(OC(=O)C2=CC(=C(C(=C2C3=C(C(=C(C=C3C(=O)O1)O)O)O)O)O)O)C4C5C(C6=C(C(=C(C(=C6C(=O)O5)C7=C(C(=C(C=C7C(=O)O4)O)O)O)O)O)O)O)OC(=O)C8=CC(=C(C(=C8)O)O)O |  | 157395 | 936.6 | Vertebrate Squalene Epoxidase | 32 µM | (Das *et al.*, 2021). |
| **34.** | **Pedunculagin**  **(34)** | C1C2C(C3C(C(O2)O)OC(=O)C4=CC(=C(C(=C4C5=C(C(=C(C=C5C(=O)O3)O)O)O)O)O)O)OC(=O)C6=CC(=C(C(=C6C7=C(C(=C(C=C7C(=O)O1)O)O)O)O)O)O |  | 442688 | 784.5 | Vertebrate Squalene Epoxidase | 2.0 µM | (Das *et al.*, 2021). |
| **35.** | **Corilagin**  **(B35)** | C1C2C(C(C(C(O2)OC(=O)C3=CC(=C(C(=C3)O)O)O)O)OC(=O)C4=CC(=C(C(=C4C5=C(C(=C(C=C5C(=O)O1)O)O)O)O)O)O)O |  | 73568 | 634.5 | Vertebrate Squalene Epoxidase | 4.0 µM | (Das *et al.*, 2021, Kinoshita *et al.*, 2007). |
| **36.** | **Tellimagrandin**  **(B36)** | C1C2C(C(C(C(O2)O)OC(=O)C3=CC(=C(C(=C3)O)O)O)OC(=O)C4=CC(=C(C(=C4)O)O)O)OC(=O)C5=CC(=C(C(=C5C6=C(C(=C(C=C6C(=O)O1)O)O)O)O)O)O |  | 442690 | 786.6 | *Oenothera erythrosepala* | 1.7 µΜ | (Das *et al.*, 2021). |
| **37.** | **Punicalin**  **(B37)** | C1C2C(C(C(C(O2)O)O)O)OC(=O)C3=CC(=C(C(=C3C4=C(C(=C5C6=C4C(=O)OC7=C(C(=C(C8=C(C(=C(C=C8C(=O)O1)O)O)O)C(=C67)C(=O)O5)O)O)O)O)O)O)O |  | 5388496 | 782.5 | *Terminalia triflora* | 0.14 µM | (Das *et al.*, 2021, Oudane *et al.*, 2018). |
| **38.** | **Punicalagin**  **(B38)** | C1C2C(C3C(C(O2)O)OC(=O)C4=CC(=C(C(=C4C5=C(C(=C(C=C5C(=O)O3)O)O)O)O)O)O)OC(=O)C6=CC(=C(C(=C6C7=C(C(=C8C9=C7C(=O)OC2=C(C(=C(C3=C(C(=C(C=C3C(=O)O1)O)O)O)C(=C92)C(=O)O8)O)O)O)O)O)O)O |  | 44584733 | 1084.7 | *Punica granatum* | 4.6 ± 0.4 µM | (Das *et al.*, 2021, Oudane *et al.*, 2018). |
| **39.** | **Methyl Gallate**  **(B39)** | COC(=O)C1=CC(=C(C(=C1)O)O)O |  | 7428 | 184.15 | *Caesalpinia pulcherrima* | 4.62 μM | (Sharma and Maity, 2010, Asnaashari *et al.*, 2014). |
| **40.** | **Ellagic acid**  **(B40)** | C1=C2C3=C(C(=C1O)O)OC(=O)C4=CC(=C(C(=C43)OC2=O)O)O |  | 5281855 | 302.19 | Vertebrate Squalene Epoxidase | 2.0 µM | (Das *et al.*, 2021, Han *et al.*, 2006). |
| **41.** | **caffeic acid**  **(B41)** | C1=CC(=C(C=C1C=CC(=O)O)O)O |  | 689043 | 180.16 | Sugar date palm *[Phoenix sylvestris (L.) Roxb.]* | 1.42 µM | (Das *et al.*, 2021, Rani *et al.*, 2018, Girsang *et al.*, 2020). |
| **42.** | **Chlorogenic acid**  **(B42)** | C1C(C(C(CC1(C(=O)O)O)OC(=O)C=CC2=CC(=C(C=C2)O)O)O)O |  | 1794427 | 354.31 | Rat liver COMT | 6.17 ± 2.23 µM | (Das *et al.*, 2021, Kweon *et al.*, 2001). |
| **43.** | **p-coumaric acid**  **(B43)** | C1=CC(=CC=C1C=CC(=O)O)O |  | 637542 | 164.16 | pomegranate *(Punica granatum)* leaves | 2.5 µM | (Das *et al.*, 2021, Zang *et al.*, 2000). |
| **44.** | **Quinic acid**  **(B44)** | C1C(C(C(CC1(C(=O)O)O)O)O)O |  | 6508 | 192.17 | Sugar date palm *[Phoenix sylvestris (L.) Roxb.]* | 4.91 µM | (Das *et al.*, 2021, Yang *et al.*, 2013). |
| **45.** | **Kaempferol**  **(B45)** | C1=CC(=CC=C1C2=C(C(=O)C3=C(C=C(C=C3O2)O)O)O)O |  | 5280863 | 286.24 | human pyruvate kinase isoenzyme M2 | 9.88 µM | (Das *et al.*, 2021, Rani *et al.*, 2018). |
| **46.** | **Myricetin**  **(B46)** | C1=C(C=C(C(=C1O)O)O)C2=C(C(=O)C3=C(C=C(C=C3O2)O)O)O |  | 5281672 | 318.23 | human pyruvate kinase isoenzyme M2 | 0.51 µM | (Sharma and Maity, 2010). |
| **47.** | **Quercetin 3-O-rutinoside**  **(B47)** | CC1C(C(C(C(O1)OCC2C(C(C(C(O2)OC3=C(OC4=CC(=CC(=C4C3=O)O)O)C5=CC(=C(C=C5)O)O)O)O)O)O)O)O |  | 5280805 | 610.5 | Inhibits Protein Disulfide Isomerase | 10 µM | (Sharma and Maity, 2010, Rani *et al.*, 2018). |
| **48.** | **Luteolin**  **(B48)** | C1=CC(=C(C=C1C2=CC(=O)C3=C(C=C(C=C3O2)O)O)O)O |  | 5280445 | 286.24 | *Brassica oleracea L*. | 0.12 µM | (Das *et al.*, 2021, Ahmadi *et al.*, 2020). |
| **49.** | **Luteolin 7-O-glucoside**  **(B49)** | C1=CC(=C(C=C1C2=CC(=O)C3=C(C=C(C=C3O2)OC4C(C(C(C(O4)CO)O)O)O)O)O)O |  | 5280637 | 448.4 | *Cuminum cyminum fruits* | 3.98 µg/mL | (Das *et al.*, 2021, Boudoukha *et al.*, 2018). |
| **50.** | **Epigallocatechin 3-gallate**  **(B50)** | C1C(C(OC2=CC(=CC(=C21)O)O)C3=CC(=C(C(=C3)O)O)O)OC(=O)C4=CC(=C(C(=C4)O)O)O |  | 65064 | 458.4 | Green Tea | 3.8 µM | (Das *et al.*, 2021, Kamiyama *et al.*, 2010). |
| **51.** | **Naringin**  **(B51)** | CC1C(C(C(C(O1)OC2C(C(C(OC2OC3=CC(=C4C(=O)CC(OC4=C3)C5=CC=C(C=C5)O)O)CO)O)O)O)O)O |  | 442428 | 580.5 | human pyruvate kinase isoenzyme M2 | 16.60 µM | (Das *et al.*, 2021, Cavia‐Saiz *et al.*, 2010). |
| **52.** | **Delphinidin**  **(B52)** | C1=C(C=C(C(=C1O)O)O)C2=[O+]C3=CC(=CC(=C3C=C2O)O)O.[Cl-] |  | 68245 | 338.69 | *Diabetes Mellitus* | 0.078 µM | (Sharma and Maity, 2010, Sauer *et al.*, 2021). |
| **53.** | **Cyanidin**  **(B53)** | C1=CC(=C(C=C1C2=[O+]C3=CC(=CC(=C3C=C2O)O)O)O)O |  | 128861 | 287.24 | *Rubus fruticosus L.* | 1.41 µM | (Sharma and Maity, 2010, Akkarachiyasit *et al.*, 2010). |
| **54.** | **Pelargonidin**  **(B54)** | C1=CC(=CC=C1C2=[O+]C3=CC(=CC(=C3C=C2O)O)O)O |  | 440832 | 271.24 | *Diabetes Mellitus* | 31.36 µM | (Sharma and Maity, 2010). |
| **55.** | **Chrysin**  **(B55)** | C1=CC=C(C=C1)C2=CC(=O)C3=C(C=C(C=C3O2)O)O |  | 5281607 | 254.24 | bee products | 0.017 ± 0.001 µg/mL | (Dawi *et al.*, 2021, Fonseca *et al.*, 2015, Arts *et al.*, 2003). |
| **56.** | **Delphinidin-3-glucoside**  **(B56)** | C1=C(C=C(C(=C1O)O)O)C2=[O+]C3=CC(=CC(=C3C=C2OC4C(C(C(C(O4)CO)O)O)O)O)O.[Cl-] |  | 165558 | 500.8 | *Diabetes Mellitus* | 59.83 µM | (Vučić *et al.*, 2019, Sauer *et al.*, 2021). |
| **57.** | **Cyanidin-3-glucoside**  **(B57)** | C1=CC(=C(C=C1C2=[O+]C3=CC(=CC(=C3C=C2OC4C(C(C(C(O4)CO)O)O)O)O)O)O)O.[Cl-] |  | 12303220 | 484.8 | *Santalum album L. berries* | 0.1 μg/mL | (Vučić *et al.*, 2019). |
| **58.** | **Gallocatechin**  **(B58)** | C1C(C(OC2=CC(=CC(=C21)O)O)C3=CC(=C(C(=C3)O)O)O)O |  | 65084 | 306.27 | *Caesalpinia pulcherrima* | 5.26 µM | (Lansky and Newman, 2007, Kamiyama *et al.*, 2010). |
| **59.** | **Hydroxycinnamic acid**  **(B59)** | C1=CC(=CC=C1C=CC(=O)O)O |  | 637542 | 164.16 | mushroom *(Agaricus bisporus)* tyrosinase | 0.50 µM | (Lansky and Newman, 2007). |
| **60.** | **Valoneic acid dilactone**  **(B60)** | C1=C2C3=C(C(=C1O)O)OC(=O)C4=CC(=C(C(=C43)OC2=O)O)OC5=C(C(=C(C=C5C(=O)O)O)O)O |  | 10151874 | 470.3 | Syzygium cumini seeds | 0.075 µg/mL | (Lansky and Newman, 2007). |
| **61.** | **Taxifolin**  **(B61)** | C1=CC(=C(C=C1C2C(C(=O)C3=C(C=C(C=C3O2)O)O)O)O)O |  | 439533 | 304.25 | sugar date palm | 0.31 ± 0.01 µM | (Farag *et al.*, 2020, Topal *et al.*, 2016). |
| **62.** | **Quercetrin**  **(B62)** | CC1C(C(C(C(O1)OC2=C(OC3=CC(=CC(=C3C2=O)O)O)C4=CC(=C(C=C4)O)O)O)O)O |  | 5280459 | 448.4 | Syzygium cumini seeds | 0.70 ± 0.10 µg/mL | (Shalaby *et al.*, 2019, Rani *et al.*, 2018). |
| **63.** | **3,4,5-trimethoxy cinnamic acid**  **(B63)** | COC1=CC(=CC(=C1OC)OC)C=CC(=O)O |  | 735755 | 238.24 | Polygala tenuifolia Wild | 6.4 µM | (Shalaby *et al.*, 2019). |
| **64.** | **Glucogallin**  **(B64)** | COC1C(C(C(C(O1)OC(=O)C2=CC(=C(C(=C2)O)O)O)O)O)O |  | 124375 | 332.26 | *Emblica officinalis* | 17 µM | (Shalaby *et al.*, 2019). |
| **65.** | **Quercimeritrin**  **(B65)** | C1=CC(=C(C=C1C2=C(C(=O)C3=C(C=C(C=C3O2)OC4C(C(C(C(O4)CO)O)O)O)O)O)O)O |  | 5282160 | 464.4 | extracts of *Cassia angustifolia Vahl* | 4.0 µg/mL | (Sharma and Maity, 2010). |
| **66.** | **Quercetrin-3-glucoside**  **(B66)** | C1=CC(=C(C=C1C2=C(C(=O)C3=C(C=C(C=C3O2)O)O)OC4C(C(C(C(O4)CO)O)O)O)O)O |  | 5280804 | 464.4 | Moringa oleifera Leaf Extract | 1.9 ± 0.1 µg/mL | (Shalaby *et al.*, 2019, Rani *et al.*, 2018). |
| **67.** | **Kaempferol 3-O-glucoside**  **(B67)** | C1=CC(=CC=C1C2=C(C(=O)C3=C(C=C(C=C3O2)O)O)OC4C(C(C(C(O4)CO)O)O)O)O |  | 5282102 | 448.4 | Moringa oleifera Leaf Extract | 3.2 ± 0.4 µg/mL | (Das *et al.*, 2021, Rani *et al.*, 2018, Tian *et al.*, 2021). |
| **68.** | **Strictinin**  **(B68)** | C1C2C(C(C(C(O2)OC(=O)C3=CC(=C(C(=C3)O)O)O)O)O)OC(=O)C4=CC(=C(C(=C4C5=C(C(=C(C=C5C(=O)O1)O)O)O)O)O)O |  | 73330 | 634.5 | green tea extract | 0.07 µM | (Singh *et al.*, 2018). |
| **69.** | **Pseudopelletierine**  **(B69)** | CN1C2CCCC1CC(=O)C2 |  | 11096 | 153.22 | Seaweeds and sponge | 4.8±1.84 µg/mL | (Usha *et al.*, 2015). |
| **70.** | **Acacetin**  **(B70)** | COC1=CC=C(C=C1)C2=CC(=O)C3=C(C=C(C=C3O2)O)O |  | 5280442 | 284.26 | *Atrial Fibrillation* | 0.8 µM | (Shalaby *et al.*, 2019). |
| **71.** | **Anthocyanin**  **(B71)** | C1=CC=C(C=C1)C2=[O+]C3=CC=CC=C3C=C2 |  | 145858 | 207.25 | purple sweet potato *(Ipomoea batatas L.)* | 3.68 ± 0.01 µg/mL | (Singh *et al.*, 2018). |
| **72.** | **Granatin A (B72)** | C1C2C3C(C(C(O2)C(=O)C4=CC(=C(C(=C4C5=C(C(=C(C=C5C(=O)O1)O)O)O)O)O)O)OC(=O)C6=CC(=O)C7(C(C6C8=C(O7)C(=C(C=C8C(=O)O3)O)O)(O)O)O)O |  | 131752596 | 784.5 | *Punica granatum* | 6.2µM | (Bellesia *et al.*, 2015). |
| **73.** | **Granatin B (B73)** | C1C2C3C(C(C(O2)OC(=O)C4=CC(=C(C(=C4)O)O)O)OC(=O)C5=CC(=O)C(C6(C5C7=C(O6)C(=C(C=C7C(=O)O3)O)O)O)(O)O)OC(=O)C8=CC(=C(C(=C8C9=C(C(=C(C=C9C(=O)O1)O)O)O)O)O)O |  | 50903199 | 952.6 | *Punica granatum* | 0.37µM | (Bellesia *et al.*, 2015). |

**Table S2:** Molecular docking analysis of active ligands

| **TYR** | **Compound** | **Moldock Score** | **H-bond** | **No of Interaction** | **Protein-ligand interaction by H-bonding & Van der Waals forces** |
| --- | --- | --- | --- | --- | --- |

| **TYR** | **Compound** | **Moldock Score** | **H-bond** | **No of Interaction** | **Protein-ligand interaction by H-bonding & Van der Waals forces** | | | | |
| --- | --- | --- | --- | --- | --- | --- | --- | --- | --- |
|  |  |  |  |  | **Amino acid**  **residues** | **Types of interaction by** | **Category** | **Types** | **Distance** |
| **1GWR** | Edaravone | -82.238 | -2.133 | 1  2  3  4  5  6  7  8  9  10  11  12  13  14  15  16  17  18  19  20  21  22 | LYS362  LYS362  ALA743  LEU744  ARG746  ARG746  TYR747  TYR747  LEU748  LEU748  LEU749  LEU749  ASP750  LYS362  ILE358  LEU372  VAL376  LEU379  MET543  ALA743  LEU744  TYR747 | H-Bond  H-Bond  H-Bond  H-Bond  H-Bond  H-Bond  H-Bond  H-Bond  H-Bond  H-Bond  H-Bond  H-Bond  H-Bond  H-Bond  Van der waal forces  Van der waal forces  Van der waal forces  Van der waal forces  Van der waal forces  Van der waal forces  Van der waal forces  Van der waal forces | Hydrogen Bond  Hydrogen Bond  Hydrogen Bond  Hydrogen Bond  Hydrogen Bond  Hydrogen Bond  Hydrogen Bond  Hydrogen Bond  Hydrogen Bond  Hydrogen Bond  Hydrogen Bond  Hydrogen Bond  Hydrogen Bond  Hydrogen Bond  Hydrophobic  Hydrophobic  Hydrophobic  Hydrophobic  Hydrophobic  Hydrophobic  Hydrophobic  Hydrophobic | Conventional Hydrogen Bond  Conventional Hydrogen Bond  Conventional Hydrogen Bond  Conventional Hydrogen Bond  Conventional Hydrogen Bond  Conventional Hydrogen Bond  Conventional Hydrogen Bond  Conventional Hydrogen Bond  Conventional Hydrogen Bond  Conventional Hydrogen Bond  Conventional Hydrogen Bond  Conventional Hydrogen Bond  Conventional Hydrogen Bond  Carbon Hydrogen Bond  Alkyl  Alkyl  Alkyl  Alkyl  Alkyl  Alkyl  Alkyl  Pi-Alkyl | 2.84508  1.9678  2.12112  2.39077  2.21727  2.35464  2.27591  2.88625  2.39738  2.02815  2.15584  2.96346  2.36224  2.83971  5.48097  5.29648  4.62044  5.39576  5.14131  4.30467  5.47831  5.15298 |
|  | Hesperidine | -144.915 | -10.836 | 1  2  3  4  5  6  7  8  9  10  11  12  13  14  15  16  17  18  19  20  21  22 | LYS362  LYS362  ALA743  LEU744  ARG746  ARG746  TYR747  TYR747  LEU748  LEU748  LEU749  LEU749  ASP750  LYS362  ILE358  LEU372  VAL376  LEU379  MET543  ALA743  LEU744  TYR747 | H-Bond  H-Bond  H-Bond  H-Bond  H-Bond  H-Bond  H-Bond  H-Bond  H-Bond  H-Bond  H-Bond  H-Bond  H-Bond  H-Bond  Van der waal forces  Van der waal forces  Van der waal forces  Van der waal forces  Van der waal forces  Van der waal forces  Van der waal forces  Van der waal forces | Hydrogen Bond  Hydrogen Bond  Hydrogen Bond  Hydrogen Bond  Hydrogen Bond  Hydrogen Bond  Hydrogen Bond  Hydrogen Bond  Hydrogen Bond  Hydrogen Bond  Hydrogen Bond  Hydrogen Bond  Hydrogen Bond  Hydrogen Bond  Hydrophobic  Hydrophobic  Hydrophobic  Hydrophobic  Hydrophobic  Hydrophobic  Hydrophobic  Hydrophobic | Conventional Hydrogen Bond  Conventional Hydrogen Bond  Conventional Hydrogen Bond  Conventional Hydrogen Bond  Conventional Hydrogen Bond  Conventional Hydrogen Bond  Conventional Hydrogen Bond  Conventional Hydrogen Bond  Conventional Hydrogen Bond  Conventional Hydrogen Bond  Conventional Hydrogen Bond  Conventional Hydrogen Bond  Conventional Hydrogen Bond  Carbon Hydrogen Bond  Alkyl  Alkyl  Alkyl  Alkyl  Alkyl  Alkyl  Alkyl  Pi-Alkyl | 2.84508  1.9678  2.12112  2.39077  2.21727  2.35464  2.27591  2.88625  2.39738  2.02815  2.15584  2.96346  2.36224  2.83971  5.48097  5.29648  4.62044  5.39576  5.14131  4.30467  5.47831  5.15298 |
|  | Apigenin | -90.733 | -11.090 | 1  2  3  4  5  6  7  8  9  10  11  12  13  14  15  16  17  18  19  20  21  22 | LYS362  LYS362  ALA743  LEU744  ARG746  ARG746  TYR747  TYR747  LEU748  LEU748  LEU749  LEU749  ASP750  LYS362  ILE358  LEU372  VAL376  LEU379  MET543  ALA743  LEU744  TYR747 | H-Bond  H-Bond  H-Bond  H-Bond  H-Bond  H-Bond  H-Bond  H-Bond  H-Bond  H-Bond  H-Bond  H-Bond  H-Bond  H-Bond  Van der waal forces  Van der waal forces  Van der waal forces  Van der waal forces  Van der waal forces  Van der waal forces  Van der waal forces  Van der waal forces | Hydrogen Bond  Hydrogen Bond  Hydrogen Bond  Hydrogen Bond  Hydrogen Bond  Hydrogen Bond  Hydrogen Bond  Hydrogen Bond  Hydrogen Bond  Hydrogen Bond  Hydrogen Bond  Hydrogen Bond  Hydrogen Bond  Hydrogen Bond  Hydrophobic  Hydrophobic  Hydrophobic  Hydrophobic  Hydrophobic  Hydrophobic  Hydrophobic  Hydrophobic | Conventional Hydrogen Bond  Conventional Hydrogen Bond  Conventional Hydrogen Bond  Conventional Hydrogen Bond  Conventional Hydrogen Bond  Conventional Hydrogen Bond  Conventional Hydrogen Bond  Conventional Hydrogen Bond  Conventional Hydrogen Bond  Conventional Hydrogen Bond  Conventional Hydrogen Bond  Conventional Hydrogen Bond  Conventional Hydrogen Bond  Carbon Hydrogen Bond  Alkyl  Alkyl  Alkyl  Alkyl  Alkyl  Alkyl  Alkyl  Pi-Alkyl | 2.84508  1.9678  2.12112  2.39077  2.21727  2.35464  2.27591  2.88625  2.39738  2.02815  2.15584  2.96346  2.36224  2.83971  5.48097  5.29648  4.62044  5.39576  5.14131  4.30467  5.47831  5.15298 |
|  | Corilagin | -149.02 | -18.877 | 1  2  3  4  5  6  7  8  9  10  11  12  13  14  15  16  17  18  19  20  21  22 | LYS362  LYS362  ALA743  LEU744  ARG746  ARG746  TYR747  TYR747  LEU748  LEU748  LEU749  LEU749  ASP750  LYS362  ILE358  LEU372  VAL376  LEU379  MET543  ALA743  LEU744  TYR747 | H-Bond  H-Bond  H-Bond  H-Bond  H-Bond  H-Bond  H-Bond  H-Bond  H-Bond  H-Bond  H-Bond  H-Bond  H-Bond  H-Bond  Van der waal forces  Van der waal forces  Van der waal forces  Van der waal forces  Van der waal forces  Van der waal forces  Van der waal forces  Van der waal forces | Hydrogen Bond  Hydrogen Bond  Hydrogen Bond  Hydrogen Bond  Hydrogen Bond  Hydrogen Bond  Hydrogen Bond  Hydrogen Bond  Hydrogen Bond  Hydrogen Bond  Hydrogen Bond  Hydrogen Bond  Hydrogen Bond  Hydrogen Bond  Hydrophobic  Hydrophobic  Hydrophobic  Hydrophobic  Hydrophobic  Hydrophobic  Hydrophobic  Hydrophobic | Conventional Hydrogen Bond  Conventional Hydrogen Bond  Conventional Hydrogen Bond  Conventional Hydrogen Bond  Conventional Hydrogen Bond  Conventional Hydrogen Bond  Conventional Hydrogen Bond  Conventional Hydrogen Bond  Conventional Hydrogen Bond  Conventional Hydrogen Bond  Conventional Hydrogen Bond  Conventional Hydrogen Bond  Conventional Hydrogen Bond  Carbon Hydrogen Bond  Alkyl  Alkyl  Alkyl  Alkyl  Alkyl  Alkyl  Alkyl  Pi-Alkyl | 2.84508  1.9678  2.12112  2.39077  2.21727  2.35464  2.27591  2.88625  2.39738  2.02815  2.15584  2.96346  2.36224  2.83971  5.48097  5.29648  4.62044  5.39576  5.14131  4.30467  5.47831  5.15298 |
|  | Ellagic acid | -95.035 | -10.422 | 1  2  3  4  5  6  7  8  9  10  11  12  13  14  15  16  17  18  19  20  21  22 | LYS362  LYS362  ALA743  LEU744  ARG746  ARG746  TYR747  TYR747  LEU748  LEU748  LEU749  LEU749  ASP750  LYS362  ILE358  LEU372  VAL376  LEU379  MET543  ALA743  LEU744  TYR747 | H-Bond  H-Bond  H-Bond  H-Bond  H-Bond  H-Bond  H-Bond  H-Bond  H-Bond  H-Bond  H-Bond  H-Bond  H-Bond  H-Bond  Van der waal forces  Van der waal forces  Van der waal forces  Van der waal forces  Van der waal forces  Van der waal forces  Van der waal forces  Van der waal forces | Hydrogen Bond  Hydrogen Bond  Hydrogen Bond  Hydrogen Bond  Hydrogen Bond  Hydrogen Bond  Hydrogen Bond  Hydrogen Bond  Hydrogen Bond  Hydrogen Bond  Hydrogen Bond  Hydrogen Bond  Hydrogen Bond  Hydrogen Bond  Hydrophobic  Hydrophobic  Hydrophobic  Hydrophobic  Hydrophobic  Hydrophobic  Hydrophobic  Hydrophobic | Conventional Hydrogen Bond  Conventional Hydrogen Bond  Conventional Hydrogen Bond  Conventional Hydrogen Bond  Conventional Hydrogen Bond  Conventional Hydrogen Bond  Conventional Hydrogen Bond  Conventional Hydrogen Bond  Conventional Hydrogen Bond  Conventional Hydrogen Bond  Conventional Hydrogen Bond  Conventional Hydrogen Bond  Conventional Hydrogen Bond  Carbon Hydrogen Bond  Alkyl  Alkyl  Alkyl  Alkyl  Alkyl  Alkyl  Alkyl  Pi-Alkyl | 2.84508  1.9678  2.12112  2.39077  2.21727  2.35464  2.27591  2.88625  2.39738  2.02815  2.15584  2.96346  2.36224  2.83971  5.48097  5.29648  4.62044  5.39576  5.14131  4.30467  5.47831  5.15298 |
|  | Kaempferol | -90.303 | -9.894 | 1  2  3  4  5  6  7  8  9  10  11  12  13  14  15  16  17  18  19  20  21  22 | LYS362  LYS362  ALA743  LEU744  ARG746  ARG746  TYR747  TYR747  LEU748  LEU748  LEU749  LEU749  ASP750  LYS362  ILE358  LEU372  VAL376  LEU379  MET543  ALA743  LEU744  TYR747 | H-Bond  H-Bond  H-Bond  H-Bond  H-Bond  H-Bond  H-Bond  H-Bond  H-Bond  H-Bond  H-Bond  H-Bond  H-Bond  H-Bond  Van der waal forces  Van der waal forces  Van der waal forces  Van der waal forces  Van der waal forces  Van der waal forces  Van der waal forces  Van der waal forces | Hydrogen Bond  Hydrogen Bond  Hydrogen Bond  Hydrogen Bond  Hydrogen Bond  Hydrogen Bond  Hydrogen Bond  Hydrogen Bond  Hydrogen Bond  Hydrogen Bond  Hydrogen Bond  Hydrogen Bond  Hydrogen Bond  Hydrogen Bond  Hydrophobic  Hydrophobic  Hydrophobic  Hydrophobic  Hydrophobic  Hydrophobic  Hydrophobic  Hydrophobic | Conventional Hydrogen Bond  Conventional Hydrogen Bond  Conventional Hydrogen Bond  Conventional Hydrogen Bond  Conventional Hydrogen Bond  Conventional Hydrogen Bond  Conventional Hydrogen Bond  Conventional Hydrogen Bond  Conventional Hydrogen Bond  Conventional Hydrogen Bond  Conventional Hydrogen Bond  Conventional Hydrogen Bond  Conventional Hydrogen Bond  Carbon Hydrogen Bond  Alkyl  Alkyl  Alkyl  Alkyl  Alkyl  Alkyl  Alkyl  Pi-Alkyl | 2.84508  1.9678  2.12112  2.39077  2.21727  2.35464  2.27591  2.88625  2.39738  2.02815  2.15584  2.96346  2.36224  2.83971  5.48097  5.29648  4.62044  5.39576  5.14131  4.30467  5.47831  5.15298 |
|  | Myricetin | -96.832 | -8.951 | 1  2  3  4  5  6  7  8  9  10  11  12  13  14  15  16  17  18  19  20  21  22 | LYS362  LYS362  ALA743  LEU744  ARG746  ARG746  TYR747  TYR747  LEU748  LEU748  LEU749  LEU749  ASP750  LYS362  ILE358  LEU372  VAL376  LEU379  MET543  ALA743  LEU744  TYR747 | H-Bond  H-Bond  H-Bond  H-Bond  H-Bond  H-Bond  H-Bond  H-Bond  H-Bond  H-Bond  H-Bond  H-Bond  H-Bond  H-Bond  Van der waal forces  Van der waal forces  Van der waal forces  Van der waal forces  Van der waal forces  Van der waal forces  Van der waal forces  Van der waal forces | Hydrogen Bond  Hydrogen Bond  Hydrogen Bond  Hydrogen Bond  Hydrogen Bond  Hydrogen Bond  Hydrogen Bond  Hydrogen Bond  Hydrogen Bond  Hydrogen Bond  Hydrogen Bond  Hydrogen Bond  Hydrogen Bond  Hydrogen Bond  Hydrophobic  Hydrophobic  Hydrophobic  Hydrophobic  Hydrophobic  Hydrophobic  Hydrophobic  Hydrophobic | Conventional Hydrogen Bond  Conventional Hydrogen Bond  Conventional Hydrogen Bond  Conventional Hydrogen Bond  Conventional Hydrogen Bond  Conventional Hydrogen Bond  Conventional Hydrogen Bond  Conventional Hydrogen Bond  Conventional Hydrogen Bond  Conventional Hydrogen Bond  Conventional Hydrogen Bond  Conventional Hydrogen Bond  Conventional Hydrogen Bond  Carbon Hydrogen Bond  Alkyl  Alkyl  Alkyl  Alkyl  Alkyl  Alkyl  Alkyl  Pi-Alkyl | 2.84508  1.9678  2.12112  2.39077  2.21727  2.35464  2.27591  2.88625  2.39738  2.02815  2.15584  2.96346  2.36224  2.83971  5.48097  5.29648  4.62044  5.39576  5.14131  4.30467  5.47831  5.15298 |
|  | Luteolin | -97.768 | -9.660 | 1  2  3  4  5  6  7  8  9  10  11  12  13  14  15  16  17  18  19  20  21  22 | LYS362  LYS362  ALA743  LEU744  ARG746  ARG746  TYR747  TYR747  LEU748  LEU748  LEU749  LEU749  ASP750  LYS362  ILE358  LEU372  VAL376  LEU379  MET543  ALA743  LEU744  TYR747 | H-Bond  H-Bond  H-Bond  H-Bond  H-Bond  H-Bond  H-Bond  H-Bond  H-Bond  H-Bond  H-Bond  H-Bond  H-Bond  H-Bond  Van der waal forces  Van der waal forces  Van der waal forces  Van der waal forces  Van der waal forces  Van der waal forces  Van der waal forces  Van der waal forces | Hydrogen Bond  Hydrogen Bond  Hydrogen Bond  Hydrogen Bond  Hydrogen Bond  Hydrogen Bond  Hydrogen Bond  Hydrogen Bond  Hydrogen Bond  Hydrogen Bond  Hydrogen Bond  Hydrogen Bond  Hydrogen Bond  Hydrogen Bond  Hydrophobic  Hydrophobic  Hydrophobic  Hydrophobic  Hydrophobic  Hydrophobic  Hydrophobic  Hydrophobic | Conventional Hydrogen Bond  Conventional Hydrogen Bond  Conventional Hydrogen Bond  Conventional Hydrogen Bond  Conventional Hydrogen Bond  Conventional Hydrogen Bond  Conventional Hydrogen Bond  Conventional Hydrogen Bond  Conventional Hydrogen Bond  Conventional Hydrogen Bond  Conventional Hydrogen Bond  Conventional Hydrogen Bond  Conventional Hydrogen Bond  Carbon Hydrogen Bond  Alkyl  Alkyl  Alkyl  Alkyl  Alkyl  Alkyl  Alkyl  Pi-Alkyl | 2.84508  1.9678  2.12112  2.39077  2.21727  2.35464  2.27591  2.88625  2.39738  2.02815  2.15584  2.96346  2.36224  2.83971  5.48097  5.29648  4.62044  5.39576  5.14131  4.30467  5.47831  5.15298 |
|  | Taxifolin | -90.961 | -9.966 | 1  2  3  4  5  6  7  8  9  10  11  12  13  14  15  16  17  18  19  20  21  22 | LYS362  LYS362  ALA743  LEU744  ARG746  ARG746  TYR747  TYR747  LEU748  LEU748  LEU749  LEU749  ASP750  LYS362  ILE358  LEU372  VAL376  LEU379  MET543  ALA743  LEU744  TYR747 | H-Bond  H-Bond  H-Bond  H-Bond  H-Bond  H-Bond  H-Bond  H-Bond  H-Bond  H-Bond  H-Bond  H-Bond  H-Bond  H-Bond  Van der waal forces  Van der waal forces  Van der waal forces  Van der waal forces  Van der waal forces  Van der waal forces  Van der waal forces  Van der waal forces | Hydrogen Bond  Hydrogen Bond  Hydrogen Bond  Hydrogen Bond  Hydrogen Bond  Hydrogen Bond  Hydrogen Bond  Hydrogen Bond  Hydrogen Bond  Hydrogen Bond  Hydrogen Bond  Hydrogen Bond  Hydrogen Bond  Hydrogen Bond  Hydrophobic  Hydrophobic  Hydrophobic  Hydrophobic  Hydrophobic  Hydrophobic  Hydrophobic  Hydrophobic | Conventional Hydrogen Bond  Conventional Hydrogen Bond  Conventional Hydrogen Bond  Conventional Hydrogen Bond  Conventional Hydrogen Bond  Conventional Hydrogen Bond  Conventional Hydrogen Bond  Conventional Hydrogen Bond  Conventional Hydrogen Bond  Conventional Hydrogen Bond  Conventional Hydrogen Bond  Conventional Hydrogen Bond  Conventional Hydrogen Bond  Carbon Hydrogen Bond  Alkyl  Alkyl  Alkyl  Alkyl  Alkyl  Alkyl  Alkyl  Pi-Alkyl | 2.84508  1.9678  2.12112  2.39077  2.21727  2.35464  2.27591  2.88625  2.39738  2.02815  2.15584  2.96346  2.36224  2.83971  5.48097  5.29648  4.62044  5.39576  5.14131  4.30467  5.47831  5.15298 |
|  | Quercetin-3-glucoside | -116.042 | -13.557 | 1  2  3  4  5  6  7  8  9  10  11  12  13  14  15  16  17  18  19  20  21  22 | LYS362  LYS362  ALA743  LEU744  ARG746  ARG746  TYR747  TYR747  LEU748  LEU748  LEU749  LEU749  ASP750  LYS362  ILE358  LEU372  VAL376  LEU379  MET543  ALA743  LEU744  TYR747 | H-Bond  H-Bond  H-Bond  H-Bond  H-Bond  H-Bond  H-Bond  H-Bond  H-Bond  H-Bond  H-Bond  H-Bond  H-Bond  H-Bond  Van der waal forces  Van der waal forces  Van der waal forces  Van der waal forces  Van der waal forces  Van der waal forces  Van der waal forces  Van der waal forces | Hydrogen Bond  Hydrogen Bond  Hydrogen Bond  Hydrogen Bond  Hydrogen Bond  Hydrogen Bond  Hydrogen Bond  Hydrogen Bond  Hydrogen Bond  Hydrogen Bond  Hydrogen Bond  Hydrogen Bond  Hydrogen Bond  Hydrogen Bond  Hydrophobic  Hydrophobic  Hydrophobic  Hydrophobic  Hydrophobic  Hydrophobic  Hydrophobic  Hydrophobic | Conventional Hydrogen Bond  Conventional Hydrogen Bond  Conventional Hydrogen Bond  Conventional Hydrogen Bond  Conventional Hydrogen Bond  Conventional Hydrogen Bond  Conventional Hydrogen Bond  Conventional Hydrogen Bond  Conventional Hydrogen Bond  Conventional Hydrogen Bond  Conventional Hydrogen Bond  Conventional Hydrogen Bond  Conventional Hydrogen Bond  Carbon Hydrogen Bond  Alkyl  Alkyl  Alkyl  Alkyl  Alkyl  Alkyl  Alkyl  Pi-Alkyl | 2.84508  1.9678  2.12112  2.39077  2.21727  2.35464  2.27591  2.88625  2.39738  2.02815  2.15584  2.96346  2.36224  2.83971  5.48097  5.29648  4.62044  5.39576  5.14131  4.30467  5.47831  5.15298 |
| **4AA6** | Edaravone | -68.848 | 0 | NO | NO | NO | N0 | NO | NO |
|  | Hesperidine | -154.322 | -4.997 | 1  2  3  4  5 | ASN232  LIG1:H5  ASN232  LYS235  LYS235 | H-Bond  H-Bond  H-Bond  Van der waal forces  Van der waal forces | Hydrogen Bond  Hydrogen Bond  Hydrogen Bond  Hydrophobic  Hydrophobic | Conventional Hydrogen Bond  Carbon Hydrogen Bond  Carbon Hydrogen Bond  Amide-Pi Stacked  Pi-Alkyl | 2.99293  2.63923  3.03879  4.40473  4.27133 |
|  | Apigenin | -90.037 | -6.071 | 1 | ASN232 | H-Bond | Hydrogen Bond | Conventional Hydrogen Bond | 2.32093 |
|  | Corilagin | -138.785 | -10.989 | 1  2  3  4  5 | ASN232  LIGI:O4  LIGI:O2  LIGI:O5  LYS235 | H-Bond  H-Bond  H-Bond  H-Bond  Van der waal forces | Hydrogen Bond  Hydrogen Bond  Hydrogen Bond  Hydrogen Bond  Hydrophobic | Conventional Hydrogen Bond  Conventional Hydrogen Bond  Carbon Hydrogen Bond  Carbon Hydrogen Bond  Pi-Alkyl | 2.31894  2.22443  2.66513  2.38899  4.78842 |
|  | Ellagic acid | -91.051 | -5.263 | 1  2  3  4  5 | SER236  LYS235  LYS235  LYS235  LYS235 | H-Bond  Van der waal forces  Van der waal forces  Van der waal forces  Van der waal forces | Hydrogen Bond  Hydrophobic  Hydrophobic  Hydrophobic  Hydrophobic | Carbon Hydrogen Bond  Pi-Alkyl  Pi-Alkyl  Pi-Alkyl  Pi-Alkyl | 3.59715  5.02048  4.22339  4.46873  5.38929 |
|  | Kaempferol | -90.650 | -6.689 | NO | NO | NO | NO | NO | NO |
|  | Myricetin | -93.106 | -14.881 | 1  2  3  4 | LYS235  SER236  LYS235  LYS235 | Van der waal forces  Van der waal forces  Van der waal forces  Van der waal forces | Hydrophobic  Hydrophobic  Hydrophobic  Hydrophobic | Amide-Pi Stacked  Amide-Pi Stacked  Pi-Alkyl  Pi-Alkyl | 4.99434  4.3425  4.7448  4.42582 |
|  | Luteolin | -95.731 | -9.758 | 1  2  3  4 | LIGI:O3  SER236  LYS235  LYS235 | H-Bond  Van der waal forces  Van der waal forces  Van der waal forces | Hydrogen Bond  Other  Hydrophobic  Hydrophobic | Conventional Hydrogen Bond  Pi-Lone Pair  Amide-Pi Stacked  Pi-Alkyl | 1.8006  2.75231  4.69094  5.46411 |
|  | Taxifolin | -89.494 | -12.094 | 1  2  3  4 | LYS235  SER236  LYS235  LYS235 | H-Bond  H-Bond  Van der waal forces  Van der waal forces | Hydrogen Bond  Hydrogen Bond  Hydrophobic  Hydrophobic | Conventional Hydrogen Bond  Carbon Hydrogen Bond  Amide-Pi Stacked  Pi-Alkyl | 2.13645  2.40987  5.54673  4.86252 |
|  | Quercetin-3-glucoside | -116.716 | -11.278 | 1  2  3  4  4 | SER236  ASN232  LYS235  LYS235  LYS235 | H-Bond  H-Bond  Van der waal forces  Van der waal forces  Van der waal forces | Hydrogen Bond  Hydrogen Bond  Hydrophobic  Hydrophobic  Hydrophobic | Conventional Hydrogen Bond  Conventional Hydrogen Bond  Pi-Alkyl  Pi-Alkyl  Pi-Alkyl | 2.4686  1.97299  4.7573  4.00137  4.94566 |
| **7KOQ** | Edaravone | -86.768 | 0.237 | 1  2  3 | TRP54  LEU118  LEU118 | Van der waal forces  Van der waal forces  Van der waal forces | Hydrophobic  Hydrophobic  Hydrophobic | Pi-Pi T-shaped  Alkyl  Pi-Alkyl | 5.01041  4.62837  5.36644 |
|  | Hesperidine | -151.961 | -20.801 | 1  2  3  4  5  6  7  8  9  10  11  12  13  14  15  16 | TYR128  LYS142  GLU188  GLU128  PHE186  TYR92  LYS142  CYS141  LYS144  TYR187  HIS140  ASN93  LYS142  LYS142  LYS144  LYS142 | H-Bond  H-Bond  H-Bond  H-Bond  H-Bond  H-Bond  H-Bond  H-Bond  H-Bond  H-Bond  H-Bond  Van der waal forces  Van der waal forces  Van der waal forces  Van der waal forces  Van der waal forces | Hydrogen Bond  Hydrogen Bond  Hydrogen Bond  Hydrogen Bond  Hydrogen Bond  Hydrogen Bond  Hydrogen Bond  Hydrogen Bond  Hydrogen Bond  Hydrogen Bond  Hydrogen Bond  Hydrophobic  Hydrophobic  Hydrophobic  Hydrophobic  Hydrophobic | Conventional Hydrogen Bond  Conventional Hydrogen Bond  Conventional Hydrogen Bond  Conventional Hydrogen Bond  Conventional Hydrogen Bond  Conventional Hydrogen Bond  Conventional Hydrogen Bond  Carbon Hydrogen Bond  Carbon Hydrogen Bond  Carbon Hydrogen Bond  Carbon Hydrogen Bond  Pi-Sigma  Pi-Sigma  Alkyl  Pi-Alkyl  Pi-Alkyl | 2.33757  2.26957  1.85872  1.9775  2.40044  2.29878  2.10278  2.90006  2.60017  1.59983  3.02534  2.70935  2.76142  4.53897  5.11404  4.20824 |
|  | Apigenin | -80.696 | -3.722 | NO | NO | NO | NO | NO | NO |
|  | Corilagin | -100.758 | -5.949 | NO | NO | NO | NO | NO | NO |
|  | Ellagic acid | -93.698 | -6.389 | 1  2  3  4  5  6 | LEU118  ASN106  LEU118  LEU118  LEU118  LEU118 | H-Bond  H-Bond  Van der waal forces  Van der waal forces  Van der waal forces  Van der waal forces | Hydrogen Bond  Hydrogen Bond  Hydrophobic  Hydrophobic  Hydrophobic  Hydrophobic | Conventional Hydrogen Bond  Conventional Hydrogen Bond  Pi-Alkyl  Pi-Alkyl  Pi-Alkyl  Pi-Alkyl | 1.83412  2.12675  4.72201  3.38603  4.64606  4.26008 |
|  | Kaempferol | -94.939 | -4.807 | 1  2  3  4  5 | ARG185  TYR187  TYR187  TYR187  LYS144 | H-Bond  Van der waal forces  Van der waal forces  Van der waal forces  Van der waal forces | Hydrogen Bond  Hydrophobic  Hydrophobic  Hydrophobic  Hydrophobic | Carbon Hydrogen Bond  Pi-Sigma  Pi-Pi T-shaped  Pi-Pi T-shaped  Pi-Alkyl | 2.76282  2.55291  4.3605  4.79433  5.40323 |
|  | Myricetin | -136.819 | -13.546 | 1  2  3  4  5 | GLN116  ASN106  LEU118  LEU118  LEU118 | H-Bond  H-Bond  H-Bond  Van der waal forces  Van der waal forces | Hydrogen Bond  Hydrogen Bond  Hydrogen Bond  Hydrophobic  Hydrophobic | Conventional Hydrogen Bond  Conventional Hydrogen Bond  Pi-Donor Hydrogen Bond  Pi-Sigma  Pi-Alkyl | 1.73914  1.73851  2.51636  2.97284  4.51847 |
|  | Luteolin | -130.862 | -10.523 | 1  2  3  4  5 | GLN116  ASN106  LEU118  LEU118  LEU118 | H-Bond  H-Bond  H-Bond  Van der waal forces  Van der waal forces | Hydrogen Bond  Hydrogen Bond  Hydrogen Bond  Hydrophobic  Hydrophobic | Conventional Hydrogen Bond  Conventional Hydrogen Bond  Pi-Donor Hydrogen Bond  Pi-Sigma  Pi-Alkyl | 1.76381  1.65444  2.42079  2.93899  4.48472 |
|  | Taxifolin | -132.978 | -13.699 | 1  2  3  4 | GLN116  ASN106  LEU118  LEU118 | H-Bond  H-Bond  H-Bond  Van der waal forces | Hydrogen Bond  Hydrogen Bond  Hydrogen Bond  Hydrophobic | Conventional Hydrogen Bond  Conventional Hydrogen Bond  Pi-Donor Hydrogen Bond  Pi-Alkyl | 2.06041  1.71667  2.62738  4.52597 |
|  | Quercetin-3-glucoside | -114.088 | -5.432 | 1  2  3  4  5  6 | LIG1:O8  ASN106  GLN116  LEU118  LEU118  LEU118 | H-Bond  H-Bond  H-Bond  H-Bond  Van der waal forces  Van der waal forces | Hydrogen Bond  Hydrogen Bond  Hydrogen Bond  Hydrogen Bond  Hydrophobic  Hydrophobic | Conventional Hydrogen Bond  Conventional Hydrogen Bond  Conventional Hydrogen Bond  Pi-Donor Hydrogen Bond  Pi-Alkyl  Pi-Alkyl | 2.32388  2.14344  1.73819  2.90647  4.07557  4.48104 |
| **5ONP** | Edaravone | -66.613 | 0 | 1  2 | TRP115  HIS146 | Van der waal forces  Van der waal forces | Hydrophobic  Hydrophobic | Pi-Alkyl  Pi-Alkyl | 5.08949  4.71607 |
|  | Hesperidine | -111.712 | -10.861 | 1  2  3  4  5  6  7  8  9  10 | ASN111  TYR193  LEU202  TYR211  TYR193  LIG1:H29  LEU202  LEU202  LEU202  TYR110 | H-Bond  H-Bond  H-Bond  H-Bond  H-Bond  H-Bond  H-Bond  H-Bond  H-Bond  Van der waal forces | Hydrogen Bond  Hydrogen Bond  Hydrogen Bond  Hydrogen Bond  Hydrogen Bond  Hydrogen Bond  Hydrogen Bond  Hydrogen Bond  Hydrogen Bond  Hydrophobic | Conventional Hydrogen Bond  Conventional Hydrogen Bond  Conventional Hydrogen Bond  Conventional Hydrogen Bond  Conventional Hydrogen Bond  Conventional Hydrogen Bond  Carbon Hydrogen Bond  Carbon Hydrogen Bond  Carbon Hydrogen Bond  Pi-Alkyl | 2.21404  2.4376  2.22058  2.34088  2.14047  1.78795  2.68205  1.37662  2.98193  4.64371 |
|  | Apigenin | -73.347 | -5.128 | 1  2  3  4  5  6  7 | ASP213  ASP213  LEU202  GLN225  ASP226  ILE232  ARG203 | H-Bond  H-Bond  H-Bond  H-Bond  Van der waal forces  Van der waal forces  Van der waal forces | Hydrogen Bond  Hydrogen Bond  Hydrogen Bond  Hydrogen Bond  Hydrophobic  Hydrophobic  Hydrophobic | Conventional Hydrogen Bond  Conventional Hydrogen Bond  Conventional Hydrogen Bond  Conventional Hydrogen Bond  Pi-Sigma  Pi-Alkyl  Pi-Alkyl | 3.31478  1.63417  2.87208  2.29758  2.0865  4.73304  5.36326 |
|  | Corilagin | -106.857 | -11.357 | 1  2  3  4  5  6  7  8 | ARG203  LEU202  HIS231  LIG1:O9  LIG1:O8  LIG1:O2  LIG1:O5  LIG1 | H-Bond  H-Bond  H-Bond  H-Bond  H-Bond  H-Bond  H-Bond  Van der waal forces | Hydrogen Bond  Hydrogen Bond  Hydrogen Bond  Hydrogen Bond  Hydrogen Bond  Hydrogen Bond  Hydrogen Bond  Hydrophobic | Conventional Hydrogen Bond  Conventional Hydrogen Bond  Conventional Hydrogen Bond  Conventional Hydrogen Bond  Carbon Hydrogen Bond  Carbon Hydrogen Bond  Carbon Hydrogen Bond  Pi-Pi Stacked | 2.00336  1.78535  2.22965  2.46595  1.62064  2.6655  2.38908  4.36827 |
|  | Ellagic acid | -87.520 | -6.939 | 1  2  3  4 | TYR157  ASN165  ASP150  ASP150 | H-Bond  H-Bond  H-Bond  H-Bond | Hydrogen Bond  Hydrogen Bond  Hydrogen Bond  Hydrogen Bond | Conventional Hydrogen Bond  Conventional Hydrogen Bond  Conventional Hydrogen Bond  Conventional Hydrogen Bond | 2.39252  1.80534  1.63103  2.25553 |
|  | Kaempferol | -72.720 | -5.018 | 1  2  3  4  5  6  7 | HIS146  LIG1:O4  HIS146  ASP150  ASP150  PHE114  TYR157 | H-Bond  H-Bond  H-Bond  H-Bond  H-Bond  Van der waal forces  Van der waal forces | Hydrogen Bond  Hydrogen Bond  Hydrogen Bond  Hydrogen Bond  Hydrogen Bond  Hydrophobic  Hydrophobic | Conventional Hydrogen Bond  Conventional Hydrogen Bond  Conventional Hydrogen Bond  Conventional Hydrogen Bond  Pi-Donor Hydrogen Bond  Pi-Pi Stacked  Pi-Pi T-shaped | 2.23727  1.79241  2.86339  2.53777  3.82306  4.99223  5.40346 |
|  | Myricetin | -74.497 | -7.208 | 1  2  3  4  5 | ASP213  LIG1:O4  HIS231  GLN225  GLN225 | H-Bond  H-Bond  H-Bond  H-Bond  H-Bond | Hydrogen Bond  Hydrogen Bond  Hydrogen Bond  Hydrogen Bond  Hydrogen Bond | Conventional Hydrogen Bond  Conventional Hydrogen Bond  Conventional Hydrogen Bond  Conventional Hydrogen Bond  Conventional Hydrogen Bond | 2.66968  1.79245  1.60494  1.98245  1.66433 |
|  | Luteolin | -75.564 | -7.413 | 1  2  3  4  5 | ARG203  ARG203  ASP226  LEU202  LEU202 | H-Bond  H-Bond  H-Bond  H-Bond  Van der waal forces | Hydrogen Bond  Hydrogen Bond  Hydrogen Bond  Hydrogen Bond  Hydrophobic | Conventional Hydrogen Bond  Conventional Hydrogen Bond  Conventional Hydrogen Bond  Conventional Hydrogen Bond  Pi-Alkyl | 2.38888  2.18748  2.80356  1.92211  5.06028 |
|  | Taxifolin | -87.204 | -9.642 | 1  2  3  4  5  6 | ASP150  TYR157  ASN165  TRP115  ASP150  ILE156 | H-Bond  H-Bond  H-Bond  H-Bond  H-Bond  H-Bond | Hydrogen Bond  Hydrogen Bond  Hydrogen Bond  Hydrogen Bond  Hydrogen Bond  Hydrogen Bond | Conventional Hydrogen Bond  Conventional Hydrogen Bond  Conventional Hydrogen Bond  Conventional Hydrogen Bond  Conventional Hydrogen Bond  Carbon Hydrogen Bond | 3.30034  2.15805  1.64095  1.62711  1.90008  2.70892 |
|  | Quercetin-3-glucoside | -85.571 | -13.921 | 1  2  3  4  5  6  7  8 | HIS146  HIS146  ASP150  LEU155  ASN227  ASN116  ASP150  ASN116 | H-Bond  H-Bond  H-Bond  H-Bond  H-Bond  H-Bond  H-Bond  Van der waal forces | Hydrogen Bond  Hydrogen Bond  Hydrogen Bond  Hydrogen Bond  Hydrogen Bond  Hydrogen Bond  Hydrogen Bond  Hydrophobic | Conventional Hydrogen Bond  Conventional Hydrogen Bond  Conventional Hydrogen Bond  Conventional Hydrogen Bond  Conventional Hydrogen Bond  Conventional Hydrogen Bond  Pi-Donor Hydrogen Bond  Pi-Sigma | 2.29948  1.98942  2.6484  1.9964  1.87601  2.05774  3.39351  2.8888 |

**Table S3: Eigenvalue of docked complexes by iMOD**

| **Complexes** | **Eigenvalue** |
| --- | --- |
| 1GWR- Corilagin | 3.531428e-04 |
| 4AA6- Hesperidine | 8.644934e-04 |
| 5ONP- Hesperidine | 2.302871e-04 |
| 7KOQ- Hesperidine | 1.292240e-05 |

| Table S4: Parameter values of B25 and B35 compound calculated by DFT (B3LYP/6-311G) | | | |
| --- | --- | --- | --- |
| Parameters | Hesperidine  (**B25**) | Corilagin  (**B35**) | |
| **E_HOMO_** | -6.2276 | -6.2256 | |
| **E_LUMO_** | -2.2996 | -1.8634 | |
| **Ionization potential (I)** | 6.2276 | 6.2256 | |
| **Electron Affinity (A)** | 2.2996 | 1.8634 | |
| **Energy gap (ΔE)** | 3.928 | 4.3622 | |
| **Absolute hardness (η)** | 1.964 | 2.1811 | |
| **Absolute softness (σ)** | 0.5091 | 0.4584 | |
| **Electronegativity (χ)** | 4.2636 | 4.0445 | |
| **chemical potential (CP)** | -4.2636 | -4.0445 | |
| **Electrophilicity index (ω)** | 4.6278 | 3.7499 | |
| **Nucleophilicity Index (N)** | 0.2160 | 0.2666 | |
| **Additional electronic charges (ΔNmax)** | 2.1708 | 1.8543 | |
| **Global softness (S)** | 0.2545 | 0.2292 | |
| **Dipole moment (Debye)** | 5.103929 | 4.644186 | |


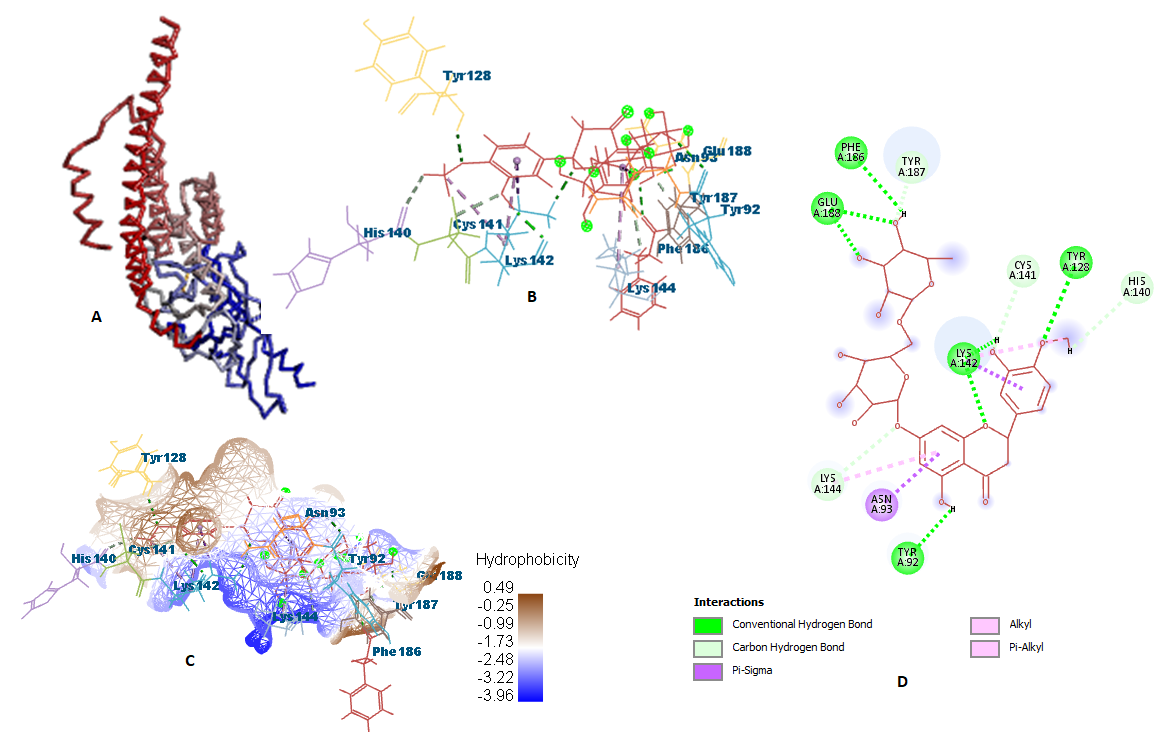


*Figure S1:* ***Protein ligand interaction of B25 with 7KOQ. (A)-Protein; (B)-Ligand interaction; (C)-Hydrophobicity; (D)-2D diagram***


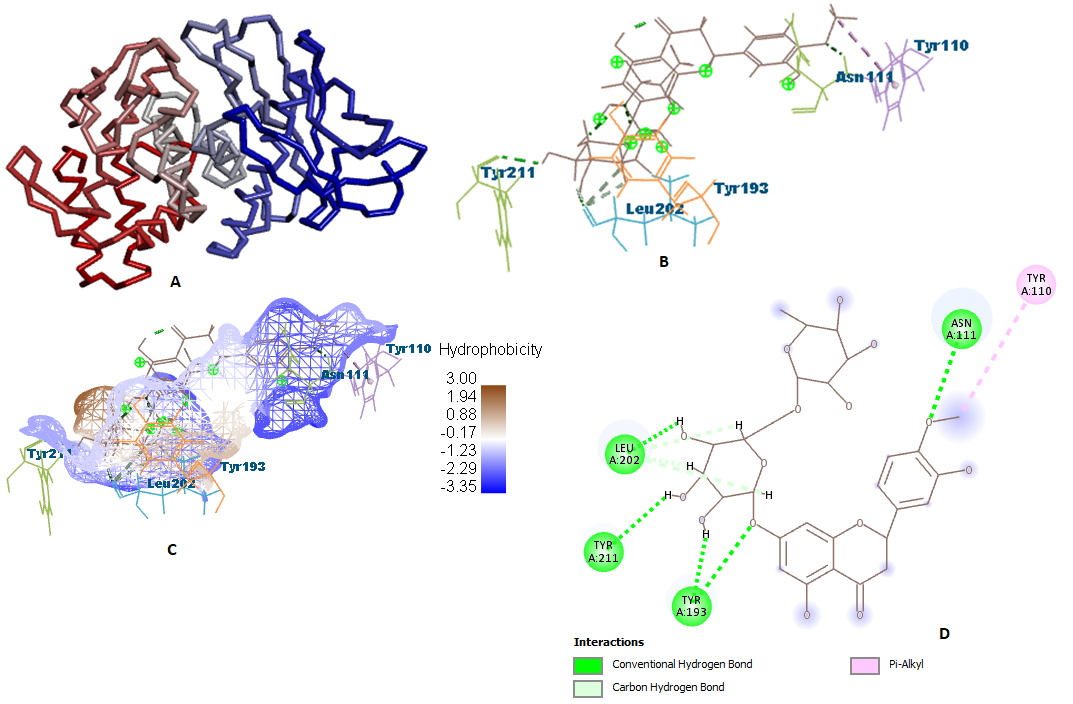


*FigureS2:* ***Protein-ligand interaction of B25 with 5ONP. (A)-Protein; (B)-Ligand interaction; (C)-Hydrophobicity; (D)-2D diagram***


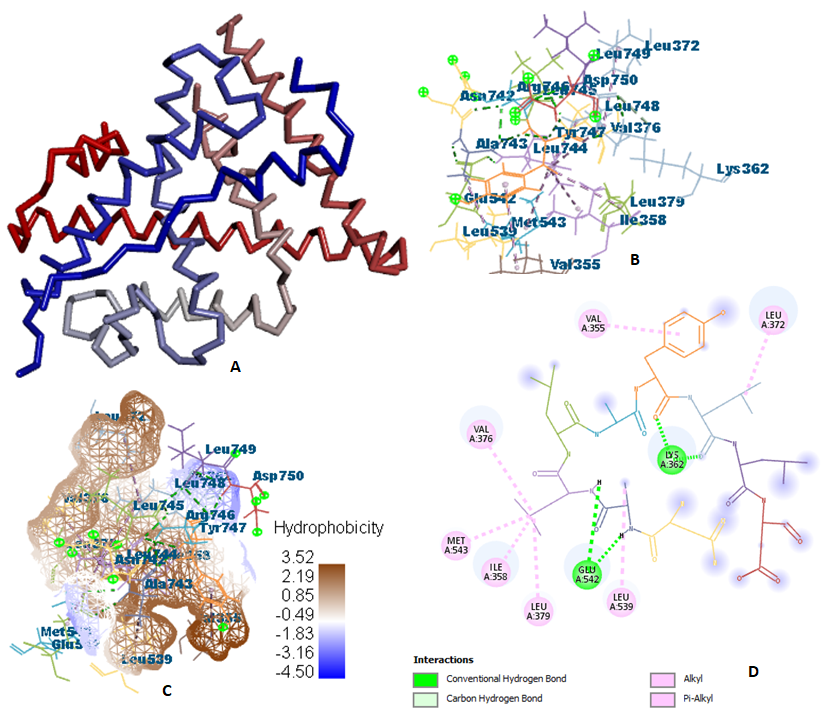


*Figure S3****: Protein-ligand interaction of B35 with 1GWR. (A)-Protein; (B)-Ligand interaction; (C)-Hydrophobicity; (D)-2D diagram***


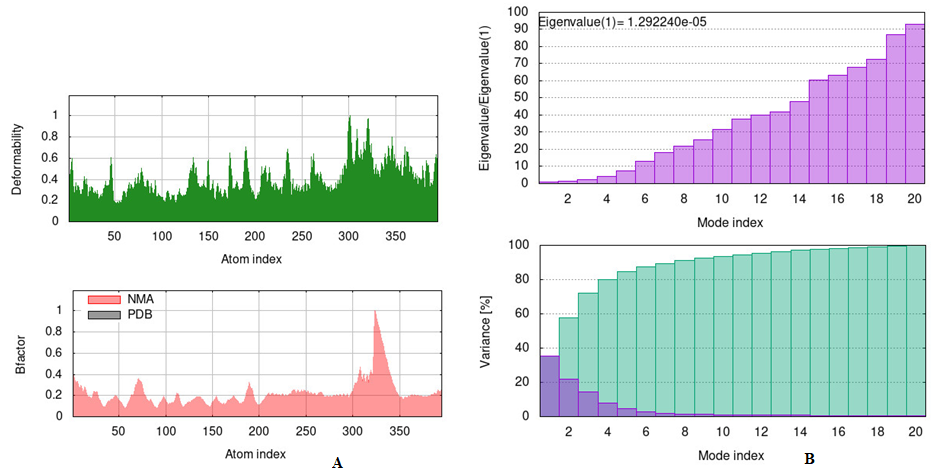


*Figure S4*: Outputs by MD simulation for 7KOQ (A) B-factor and deformability (B) Variance and eigenvalue graph


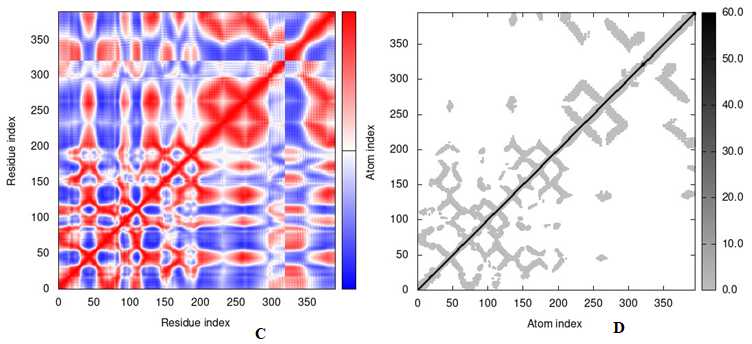


*Figure S5*: Outputs by MD simulation for 7KOQ (C) Network model; (D) Co-variance map


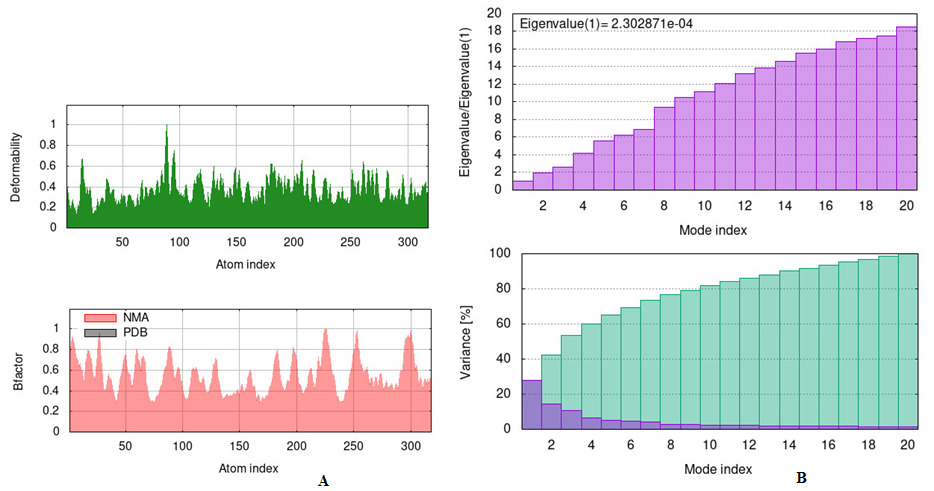


*Figure S6*: Outputs by MD simulation for 5ONP (A) B-factor and deformability (B) Variance and eigenvalue graph


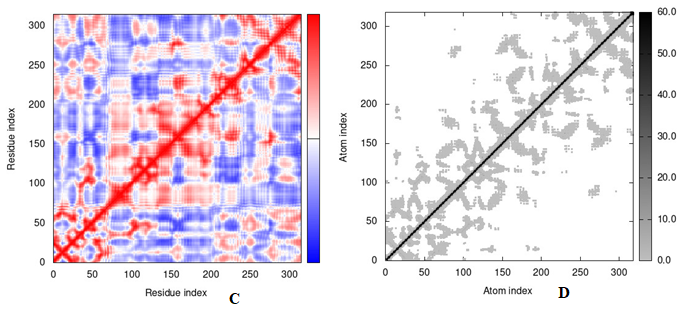


*Figure S7*: Outputs by MD simulation for 5ONP (C) Network model (D) Co-variance map.


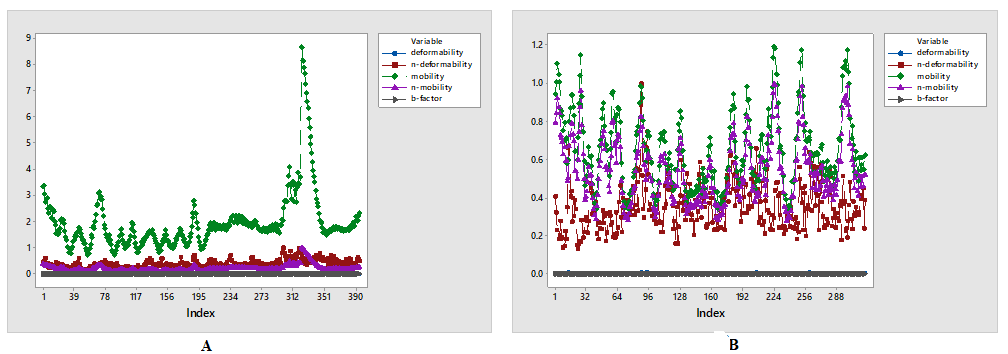


*Figure S8*: RMSF overlapping graph of (A) 7KOQ (B) 5ONP


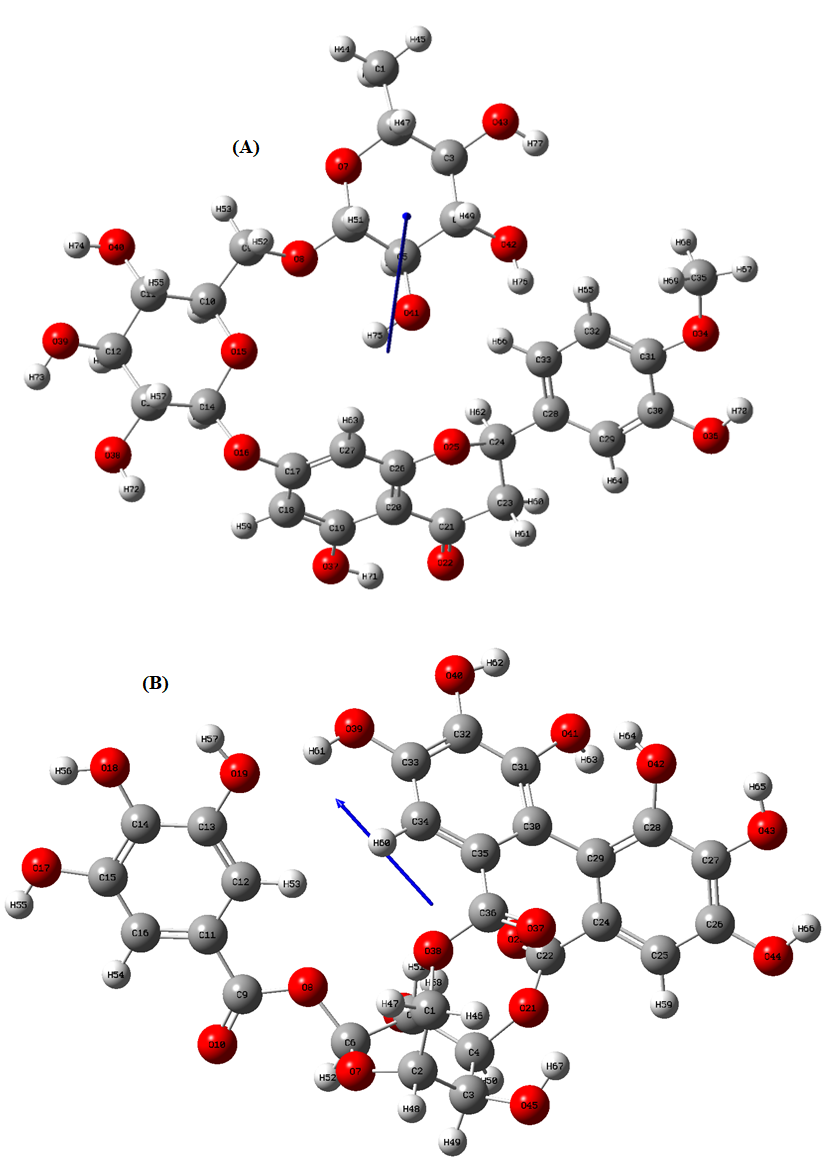


*Figure S9****: Optimization structure of (A) Hesperidine, (B) Corilagin***

**
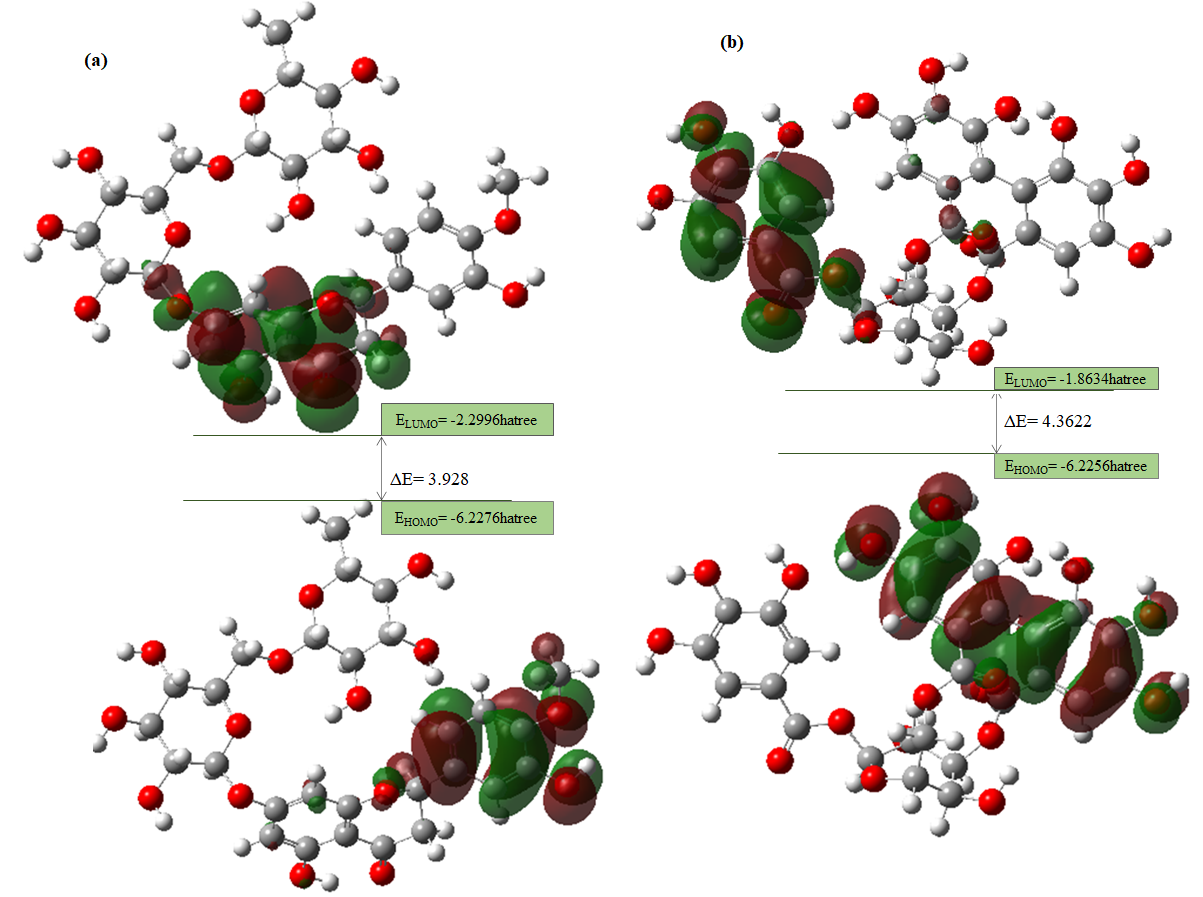
**

*Figure S10****: FMOs diagram along with energy gap (A) Hesperidine (B) Corilagin***


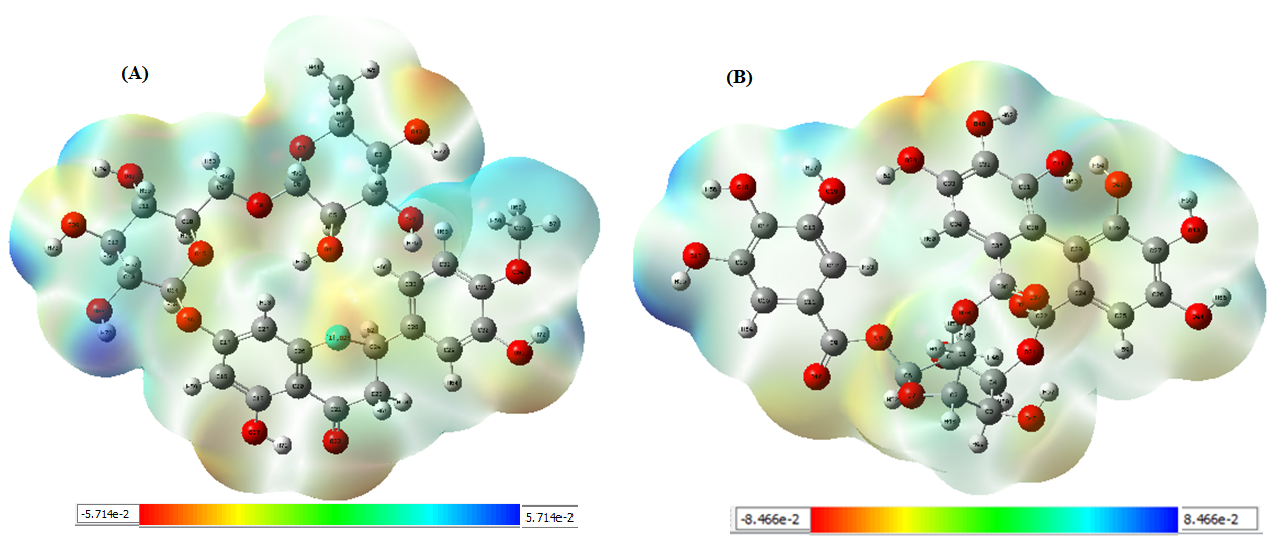


***Figure S11****: MEP structure and scale (A) Hesperidine (B) Corilagin*

Adomako-Bonsu, A. G., S. L. Chan, M. Pratten & J. R. Fry. 2017. Antioxidant activity of rosmarinic acid and its principal metabolites in chemical and cellular systems: Importance of physico-chemical characteristics. Toxicol In Vitro. 40: 248-255.

Ahmadi, S. M., R. Farhoosh, A. Sharif & M. Rezaie. 2020. Structure‐antioxidant activity relationships of luteolin and catechin. Journal of food science. 85 (2): 298-305.

Akberova, S. I., P. I. M. Galbinur, O. G. Stroeva, N. M. Magomedov, N. F. Babaev & A. P. Galbinur. 201. Comparative evaluation of the antioxidant activity of para-aminobenzoic acid and emoxipin in the cornea and crystalline lens (an experimental study. Comparative Study Vestn Oftalmol. 117 (4): 25-29.

Akkarachiyasit, S., P. Charoenlertkul, S. Yibchok-Anun & S. Adisakwattana. 2010. Inhibitory activities of cyanidin and its glycosides and synergistic effect with acarbose against intestinal α-glucosidase and pancreatic α-amylase. International journal of molecular sciences. 11 (9): 3387-3396.

Alrashdi, A. M., A. D. Al-Qurashi, M. A. Awad, S. A. Mohamed & A. A. Al-Rashdi. 2017. Quality, antioxidant compounds, antioxidant capacity and enzymes activity of ‘El-Bayadi’table grapes at harvest as affected by preharvest salicylic acid and gibberellic acid spray. Scientia Horticulturae. 220: 243-249.

Arts, M. J., J. S. Dallinga, H.-P. Voss, G. R. Haenen & A. Bast. 2003. A critical appraisal of the use of the antioxidant capacity (TEAC) assay in defining optimal antioxidant structures. Food chemistry. 80 (3): 409-414.

Asnaashari, M., R. Farhoosh & A. Sharif. 2014. Antioxidant activity of gallic acid and methyl gallate in triacylglycerols of Kilka fish oil and its oil-in-water emulsion. Food chemistry. 159: 439-444.

Badhani, B., N. Sharmaa & R. Kakkar. 2015. Gallic acid: a versatile antioxidant with promising therapeutic and industrial applications. RSC Adv.,. 5: 27540-27557.

Bellesia, A., E. Verzelloni & D. Tagliazucchi. 2015. Pomegranate ellagitannins inhibit α-glucosidase activity in vitro and reduce starch digestibility under simulated gastro-intestinal conditions. International Journal of Food Sciences and Nutrition. 66 (1): 85-92.

Boudoukha, C., H. Bouriche, M. Elmastas, H. Aksit, O. Kayir & N. Genc. 2018. Antioxidant activity of polyphenolic leaf extract from Santolina chamaecyparissus L.(Asteraceae) and the isolated luteolin-7-O-glucoside. Journal of Pharmaceutical Research International. 22 (3).

Brodowska, K., A. Sykuła, E. Garribba, E. Łodyga-Chruścińska & M. Sójka. 2016. Naringenin Schiff base: antioxidant activity, acid–base profile, and interactions with DNA. Transition Metal Chemistry. 41: 179-189.

Cavia-Saiz, M., M. D. Busto, M. C. Pilar-Izquierdo, N. Ortega, M. Perez-Mateos & P. Muñiz. 2010. Antioxidant properties, radical scavenging activity and biomolecule protection capacity of flavonoid naringenin and its glycoside naringin: a comparative study. J Sci Food Agric. 90 (7): 1238-1244.

Cavia‐Saiz, M., M. D. Busto, M. C. Pilar‐Izquierdo, N. Ortega, M. Perez‐Mateos & P. Muñiz. 2010. Antioxidant properties, radical scavenging activity and biomolecule protection capacity of flavonoid naringenin and its glycoside naringin: a comparative study. Journal of the Science of Food and Agriculture. 90 (7): 1238-1244.

Chen, Y., Y. Miao, L. Huang, J. Li, H. Sun, Y. Zhao, J. Yang & W. Zhou. 2014. Antioxidant activities of saponins extracted from Radix Trichosanthis: an in vivo and in vitro evaluation. BMC Complement Altern Med. 14: 86-64.

Das, A. K., P. K. Nanda, N. R. Chowdhury, P. Dandapat, M. Gagaoua, P. Chauhan, M. Pateiro & J. M. Lorenzo. 2021. Application of pomegranate by-products in muscle foods: Oxidative indices, colour stability, shelf life and health benefits. Molecules. 26 (2): 467.

Dawi, F., H. S. El-Beltagi, Y. E. Abdel-Mobdy, S. M. Salah, I. S. Ghaly, E. A. Abdel-Rahim, H. I. Mohamed & A. M. Soliman. 2021. Synergistic impact of the pomegranate peels and its nanoparticles against the infection of tobacco mosaic virus (TMV). Fresenius Environmental Bulletin. 30 (1): 731-746.

Ergün, B. Ç., T. Çoban, F. K. Onurdag & E. Banoglu. 2011. Synthesis, antioxidant and antimicrobial evaluation of simple aromatic esters of ferulic acid. Archives of Pharmacal Research. 34: 1251-1261.

Farag, R. S., M. S. Abdel-Latif, H. H. Abd El Baky & L. S. Tawfeek. 2020. Phytochemical screening and antioxidant activity of some medicinal plants’ crude juices. Biotechnology Reports. 28: e00536.

Fonseca, S. F., D. B. Lima, D. Alves, R. G. Jacob, G. Perin, E. J. Lenardao & L. Savegnago. 2015. Synthesis, characterization and antioxidant activity of organoselenium and organotellurium compound derivatives of chrysin. New Journal of Chemistry. 39 (4): 3043-3050.

Garcia, C. & C. N. Blesso. 2021. Antioxidant properties of anthocyanins and their mechanism of action in atherosclerosis. Free Radical Biology and Medicine. 172 (20): 152-166.

Gaspar, A., M. Martins, P. Silva, E. M. Garrido, J. Garrido, O. Firuzi, R. Miri, L. Saso & F. Borges. 2010. Dietary phenolic acids and derivatives. Evaluation of the antioxidant activity of sinapic acid and its alkyl esters. Journal of agricultural and food chemistry. 58 (21): 11273-11280.

Gęgotek, A. & E. Skrzydlewska. 2022. Antioxidative and Anti-Inflammatory Activity of Ascorbic Acid. Antioxidants. 11 (10): 1993.

Girsang, E., I. N. E. Lister, C. N. Ginting, I. A. Sholihah, M. A. Raif, S. Kunardi, H. Million & W. Widowati. 2020. Antioxidant and antiaging activity of rutin and caffeic acid. Pharmaciana. 10 (2): 147-56.

Han, D. H., M. J. Lee & J. H. Kim. 2006. Antioxidant and apoptosis-inducing activities of ellagic acid. Anticancer research. 26 (5A): 3601-3606.

Islam, M. Z., B.-J. Park & Y.-T. Lee. 2021. Bioactive Phytochemicals and Antioxidant Capacity of Wheatgrass Treated with Salicylic Acid under Organic Soil Cultivation. Chemistry and Biodiversity. 18: 1-9.

Jug, U., K. Naumoska & I. Vovk. 2021. (−)-Epicatechin—An Important Contributor to the Antioxidant Activity of Japanese Knotweed Rhizome Bark Extract as Determined by Antioxidant Activity-Guided Fractionation. Antioxidants. 10 (1): 133-153.

Kalpana, K., M. Srinivasan & V. P. Menon. 2009. Evaluation of antioxidant activity of hesperidin and its protective effect on H 2 O 2 induced oxidative damage on pBR322 DNA and RBC cellular membrane. Molecular and cellular biochemistry. 323: 21-29.

Kamiyama, O., F. Sanae, K. Ikeda, Y. Higashi, Y. Minami, N. Asano, I. Adachi & A. Kato. 2010. In vitro inhibition of α-glucosidases and glycogen phosphorylase by catechin gallates in green tea. Food chemistry. 122 (4): 1061-1066.

Kinoshita, S., Y. Inoue, S. Nakama, T. Ichiba & Y. Aniya. 2007. Antioxidant and hepatoprotective actions of medicinal herb, Terminalia catappa L. from Okinawa Island and its tannin corilagin. Phytomedicine. 14 (11): 755-762.

Kumar, S., P. Prahalathan & B. Raja. 2011. Antihypertensive and antioxidant potential of vanillic acid, a phenolic compound in L-NAME-induced hypertensive rats: a dose-dependence study. Redox Rep. 16 (5): 208-215.

Kweon, M.-H., H.-J. Hwang & H.-C. Sung. 2001. Identification and antioxidant activity of novel chlorogenic acid derivatives from bamboo (Phyllostachys edulis). Journal of agricultural and food chemistry. 49 (10): 4646-4655.

Lansky, E. P. & R. A. Newman. 2007. Punica granatum (pomegranate) and its potential for prevention and treatment of inflammation and cancer. Journal of ethnopharmacology. 109 (2): 177-206.

Magangana, T. P., N. P. Makunga, O. A. Fawole & U. L. Opara. 2020. Processing factors affecting the phytochemical and nutritional properties of pomegranate (Punica granatum L.) peel waste: A review. Molecules. 25 (20): 4690.

Munteanu, I. G. & C. Apetrei. 2022. Assessment of the Antioxidant Activity of Catechin in Nutraceuticals: Comparison between a Newly Developed Electrochemical Method and Spectrophotometric Methods. Int. J. Mol. Sci. 2022, 23(15), 8110. 23 (15): 8110-8130.

Natella, F., M. Nardini, M. D. Felice & C. Scaccini. 1999. Benzoic and cinnamic acid derivatives as antioxidants: structure-activity relation. Comparative Study J Agric Food Chem. 47 (7): 1453-1459.

Nzogong, R. T., F. S. T. Ndjateu, S. E. Ekom, J.-a. M. Fosso, M. D. Awouafack, M. Tene, P. Tane, H. Morita, M. I. Choudhary & J.-D.-D. Tamokou. 2018. Antimicrobial and antioxidant activities of triterpenoid and phenolic derivatives from two Cameroonian Melastomataceae plants: Dissotis senegambiensis and Amphiblemma monticola. BMC Complementary and Alternative Medicine. 18 (159): 1-11.

Oudane, B., D. Boudemagh, M. Bounekhel, W. Sobhi, M. Vidal & S. Broussy. 2018. Isolation, characterization, antioxidant activity, and protein-precipitating capacity of the hydrolyzable tannin punicalagin from pomegranate yellow peel (Punica granatum). Journal of Molecular Structure. 1156: 390-396.

Padumadasa, C., D. Dharmadana, A. Abeysekera & M. Thammitiyagodage. 2016. In vitro antioxidant, anti-inflammatory and anticancer activities of ethyl acetate soluble proanthocyanidins of the inflorescence of Cocos nucifera L. BMC complementary and alternative medicine. 16: 1-6.

Parhiz, H., A. Roohbakhsh, F. Soltani, R. Rezaee & M. Iranshahi. 2015. Antioxidant and anti-inflammatory properties of the citrus flavonoids hesperidin and hesperetin: an updated review of their molecular mechanisms and experimental models. Phytother Res. 29 (3): 223-231.

Park, E.-S., J. C. Kang, Y. C. Jang, J. S. Park, S. Y. Jang, D.-E. Kim, B. Kim & H.-S. Shin. 2014. Cardioprotective effects of rhamnetin in H9c2 cardiomyoblast cells under H2O2-induced apoptosis. Journal of ethnopharmacology. 153 (3): 552-560.

Peperidou, A., E. Pontiki, D. Hadjipavlou-Litina, E. Voulgari & K. Avgoustakis. 2017. Multifunctional cinnamic acid derivatives. Molecules. 22 (8): 1247.

Pietta, P. 2000. Flavonoids as antioxidants. J Nat Prod. 63 (7): 1035-42.

Rani, R., S. Arora, J. Kaur & R. K. Manhas. 2018. Phenolic compounds as antioxidants and chemopreventive drugs from Streptomyces cellulosae strain TES17 isolated from rhizosphere of Camellia sinensis. BMC Complementary and Alternative Medicine. 18 (82): 1-8.

Sarikaya, S. B. O. 2015. Acethylcholinesterase inhibitory potential and antioxidant properties of pyrogallol. Journal of Enzyme Inhibition and Medicinal Chemistry. 30 (5): 761-766.

Sauer, R.-S., I. Krummenacher, E. E. Bankoglu, S. Yang, B. Oehler, F. Schöppler, M. Mohammadi, P. Güntzel, A. Ben-Kraiem & U. Holzgrabe. 2021. Stabilization of delphinidin in complex with sulfobutylether-β-cyclodextrin allows for antinociception in inflammatory pain. Antioxidants & Redox Signaling. 34 (16): 1260-1279.

Shalaby, M., D. Dawood, M. Hefni & B. M. Murad. 2019. Phytochemical Constituents, Antimicrobial and Antitumor Effects of Pomegranate Fruit (Punica granatum L). Journal of food and dairy Sciences. 10 (10): 373-380.

Sharma, J. & A. Maity. 2010. Pomegranate phytochemicals: Nutraceutical and therapeutic values. Fruit Veg Cereal Sci Biotech. 4 (2): 56-76.

Singh, B., J. P. Singh, A. Kaur & N. Singh. 2018. Phenolic compounds as beneficial phytochemicals in pomegranate (Punica granatum L.) peel: A review. Food chemistry. 261: 75-86.

Smolyaninov, I. V., D. A. Burmistrova, M. V. Arsenyev, M. A. Polovinkina, N. P. Pomortseva, G. K. Fukin, A. I. Poddel'sky & N. T. Berberova. 2022. Synthesis and Antioxidant Activity of New Catechol Thioethers with the Methylene Linker. Molecules. 27 (10): 3196-3204.

Sova, M. 2012. Antioxidant and antimicrobial activities of cinnamic acid derivatives. Mini Rev Med Chem. 12 (8): 749-767.

Tian, C., X. Liu, Y. Chang, R. Wang, T. Lv, C. Cui & M. Liu. 2021. Investigation of the anti-inflammatory and antioxidant activities of luteolin, kaempferol, apigenin and quercetin. South African Journal of Botany. 137: 257-264.

Topal, F., M. Nar, H. Gocer, P. Kalin, U. M. Kocyigit, İ. Gülçin & S. H. Alwasel. 2016. Antioxidant activity of taxifolin: an activity–structure relationship. Journal of enzyme inhibition and medicinal chemistry. 31 (4): 674-683.

Tshane, L. S., S. S. Mashele, G. R. Matowane, S. L. Bonnet, T. J. Makhafola, A. E. Noreljaleel, S. S. Swain, M. Sekhoacha & C. I. Chukwuma. 2021. Zinc (II) mineral increased the in vitro, cellular and ex vivo antihyperglycemic and antioxidative pharmacological profile of p‐hydroxybenzoic acid upon complexation. Journal of Food Biochemistry. 45 (2): e13609.

Usha, T., A. K. Goyal, S. Lubna, H. Prashanth, T. M. Mohan, V. Pande & S. K. Middha. 2015. Identification of anti-cancer targets of eco-friendly waste Punica granatum peel by dual reverse virtual screening and binding analysis. Asian Pacific Journal of Cancer Prevention. 15 (23): 10345-10350.

Vučić, V., M. Grabež, A. Trchounian & A. Arsić. 2019. Composition and potential health benefits of pomegranate: a review. Current pharmaceutical design. 25 (16): 1817-1827.

Wang, X., X. Li & D. Chen. 2011. Evaluation of antioxidant activity of isoferulic acid in vitro. Nat Prod Commun. 6 (9): 1285-1288.

Xu, D., M.-J. Hu, Y.-Q. Wang & Y.-L. Cui. 2019. Antioxidant Activities of Quercetin and Its Complexes for Medicinal Application. Molecules. 24 (6): 1123-1143.

Yang, J., J. Guo & J. Yuan. 2008. In vitro antioxidant properties of rutin. LWT - Food Science and Technology. 41 (6): 1060-1066.

Yang, Y.-J., X. Liu, H.-R. Wu, X.-F. He, Y.-R. Bi, Y. Zhu & Z.-L. Liu. 2013. Radical scavenging activity and cytotoxicity of active quinic acid derivatives from Scorzonera divaricata roots. Food chemistry. 138 (2-3): 2057-2063.

Yassin, M. T., A. a.-F. Mostafa & A. A. Al Askar. 2021. In Vitro Evaluation of Biological Activities and Phytochemical Analysis of Different Solvent Extracts of Punica granatum L.(Pomegranate) Peels. Plants. 10 (12): 2742.

Zang, L.-Y., G. Cosma, H. Gardner, X. Shi, V. Castranova & V. Vallyathan. 2000. Effect of antioxidant protection by p-coumaric acid on low-density lipoprotein cholesterol oxidation. American Journal of Physiology-Cell Physiology. 279 (4): C954-C960.

Zhang, S., Z. Gai, T. Gui, J. Chen, Q. Chen & Y. Li. 2021. Antioxidant Effects of Protocatechuic Acid and Protocatechuic Aldehyde: Old Wine in a New Bottle. Evid Based Complement Alternat Med. 2021: 1-10.

Zhu, F., T. Asada, A. Sato, Y. Koi, H. Nishiwaki & H. Tamura. 2014. Rosmarinic acid extract for antioxidant, antiallergic, and α-glucosidase inhibitory activities, isolated by supramolecular technique and solvent extraction from Perilla leaves. Journal of agricultural and food chemistry. 62 (4): 885-892.

Zuo, A., Y. Yanying, J. Li, X. Binbin, Y. Xiongying, Q. Yan & C. Shuwen. 2011. Study on the relation of structure and antioxidant activity of isorhamnetin, quercetin, phloretin, silybin and phloretin isonicotinyl hydrazone. Free Radicals and Antioxidants. 1 (4): 39-47.
